# *In Cellulo* Cysteine Umpolung for Protein Structure Probing

**DOI:** 10.1021/jacs.5c10259

**Published:** 2025-09-19

**Authors:** Philipp Hartmann, Kostiantyn Bohdan, Lara Vogelsang, Dario Marchionni, Christian Preisinger, Alessandro Vetere, Karl-Josef Dietz, Tobias Ritter

**Affiliations:** † 28314 Max-Planck-Institut für Kohlenforschung, Kaiser-Wilhelm-Platz 1, 45470 Mülheim an der Ruhr, Germany; ‡ Institute of Organic Chemistry, RWTH Aachen University, Landoltweg 1, 52074 Aachen, Germany; § Biochemistry and Physiology of Plants, Faculty of Biology, Bielefeld University, Universitätsstraße 25, 33615 Bielefeld, Germany; ∥ Proteomics Facility, Interdisciplinary Centre for Clinical Research (IZKF), RWTH Aachen University, 52074 Aachen, Germany

## Abstract

Proteome-wide intramolecular cross-linking of amino acids allows for protein structure probing and is often achieved by reactive intermediates generated *in situ*. For example, carbene insertion chemistry forms versatile products but relies on two-step protocols that first introduce a photoreactive group and then generate a reactive intermediate via UV light irradiation. Alternatively, direct cross-linking with bifunctional electrophilic reagents enables modification of natural amino acids in a single transformation but proceeds via intermediates with a sufficiently long half-life in solution to capture unfavorable protein conformations. Herein, we report the use of commercially available vinyl thianthrenium tetrafluoroborate (**VTT**) for a single-step cross-linking strategy based on the umpolung of native cysteinyl thiols to electrophilic episulfonium ions in cells. We demonstrate that the umpolung reaction proceeds within minutes in various cell types. Even when competing with cysteine-reactive maleimide or iodoacetamide reagents, the fast cellular uptake of **VTT** enables efficient intracellular labeling and ensures the formation of products with a short and stable ethylene linker between cysteine and nucleophilic natural amino acids. We anticipate that the fast generation of episulfonium ions, which enable cross-linking without exogenous activation, will assist researchers in predicting protein structures based on quantitative proteome-wide information generated *in cellulo*.

## Graphical abstract

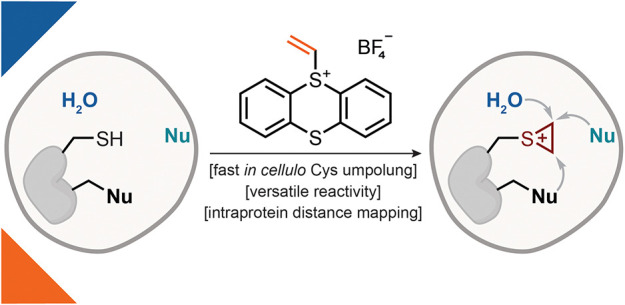

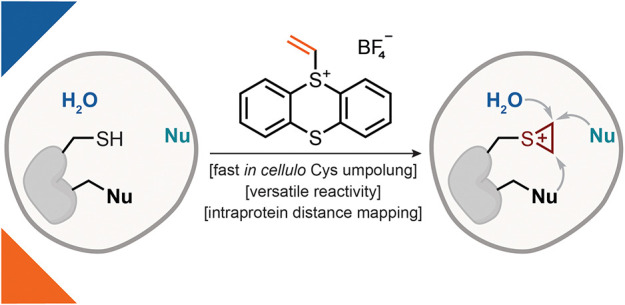

## Introduction

Protein structure prediction is an important tool for drug design and protein interaction simulations when experimental structures are not easily accessible.^1^ When no accurate structure prediction can be achieved, distance restraints between neighboring amino acids can be obtained from cross-linking studies to improve the quality of the protein structure modeling.^2^ Intermediates with short half-lives, such as carbenes, can be generated on protein surfaces upon UV-irradiation of suitable precursors like diazirines to provide tight distance restraints for cross-linking studies.^3^ The incorporation of the required precursors can be achieved by genetic code expansion, metabolic labeling experiments, or the employment of heterobifunctional small molecules. Therefore, an efficient cross-linking protocol is a two-step process that requires optimization of precursor incorporation and the need for UV-light to yield the active species. Herein, we report the use of cell-permeable vinyl thianthrenium salts that form reactive episulfonium intermediates upon reaction with native cysteine (Cys) residues in various cell types. The episulfonium ion traps neighboring amino acids or exogenous nucleophiles in a single step without the need for extracellular activation. The experimental strategy may be the starting point for *in cellulo* cross-linking studies that potentially expand knowledge of the interactome in cells. Based on the known reactivity of episulfoniums generated through thiol addition to **VTT** ^4^, the fundamental advance described here is that **VTT** is at least as efficient as *N*-ethyl maleimide (**NEM**) and superior compared to *N*-hex-5-ynyl-2-iodoacetamide (**IAA** alkyne) in penetrating cells to enable versatile modifications *in cellulo*. The suitability of **VTT** is demonstrated for several different nonhuman and human cell types, and the reactivity of the formed episulfonium ions to obtain short distance restraints for proteins *in cellulo* is highlighted.

Chemoproteomic methods rely on site-selective reactions between electrophilic reagents and nucleophilic amino acids such as cysteine and lysine (Lys).^5^ For example, tagging of hyper-reactive amino acids can be used to identify new potential drug targets in living cells^6,7^ and elucidate protein functions.^8^ The concept of chemical protein conjugation has been extended to the extraction of distances between residues to enable studies of protein conformations in cells.^9,10^ Most of the protocols utilize bifunctional cross-linkers to target lysine residues because of their high abundance and solvent accessibility.^11^ The obtained cross-linked products can then be identified and characterized to deliver information on the distances between the cross-linked amino acids within the protein structure.^9^ However, the long lifetime of the lysine-selective electrophiles can lead to formation of cross-links after adopting unfavorable conformations and thereby provide distance information on non-native conformations.^12,13^ Subsequent structure modeling is further complicated by the typical 8–12 Å linker length that, combined with the high flexibility of the lysine side chain, produces geometrical restraints that exceed 30 Å.^11,14,15^ More reliable results can be obtained by using more reactive intermediates that possess only a short half-life in solution.^3^ Photoreactive amino acids, for example, produce short cross-links with various neighboring amino acids upon light irradiation and are less prone to capturing artificial conformations.^12^ The protocols to incorporate unnatural amino acids apply techniques like metabolic labeling or amber codon suppression, which often require optimization for a particular cell type.^16^ Optimization is also necessary to ensure high incorporation rates and low structural perturbations of the photoreactive amino acid of interest. Moreover, selective formation of short-living diazo intermediates for residue-specific cross-linking requires careful control of light intensity and exposure time with specialized setups.^17^ The use of exogenous chemical cross-linkers would enable a more general approach that can be directly translated to different cell types. Sites of interest could be mapped by site-specific incorporation of canonical amino acids without the need for the utilization of unnatural amino acids. However, no method can currently achieve a fast and site-selective *in situ* formation of highly reactive intermediates *in cellulo* in a single step and without the need of external stimuli, like UV light. Herein, we demonstrate the usage of the cysteine-selective vinyl thianthrenium reagent **VTT** for *in cellulo* formation of electrophilic episulfonium ions that provide quantitative data on protein abundances and topologies without the need of exogenous stimuli and solely based on natural amino acids.

Vinyl thianthrenium tetrafluoroborate^18^ (**VTT**) is a water-soluble Michael acceptor that enables umpolung of cysteinyl thiols into reactive episulfonium ions in water.^4^ The episulfonium intermediate can then trap nucleophiles such as azide, even electron-deficient anilines, or neighboring nucleophilic amino acids, to yield bioconjugates or macrocyclic peptides with a short and stable ethylene linkage. If no suitable nucleophile is accessible, the fleeting episulfonium intermediates undergo rapid hydrolysis in an aqueous setup.^4,19^ We hypothesized that episulfonium-mediated cross-linking could be a general alternative to methods that utilize photoreactive groups for protein structure probing in various cell types without reliance on unnatural amino acids and UV light exposure (Figure 1a).

**Figure 1:**
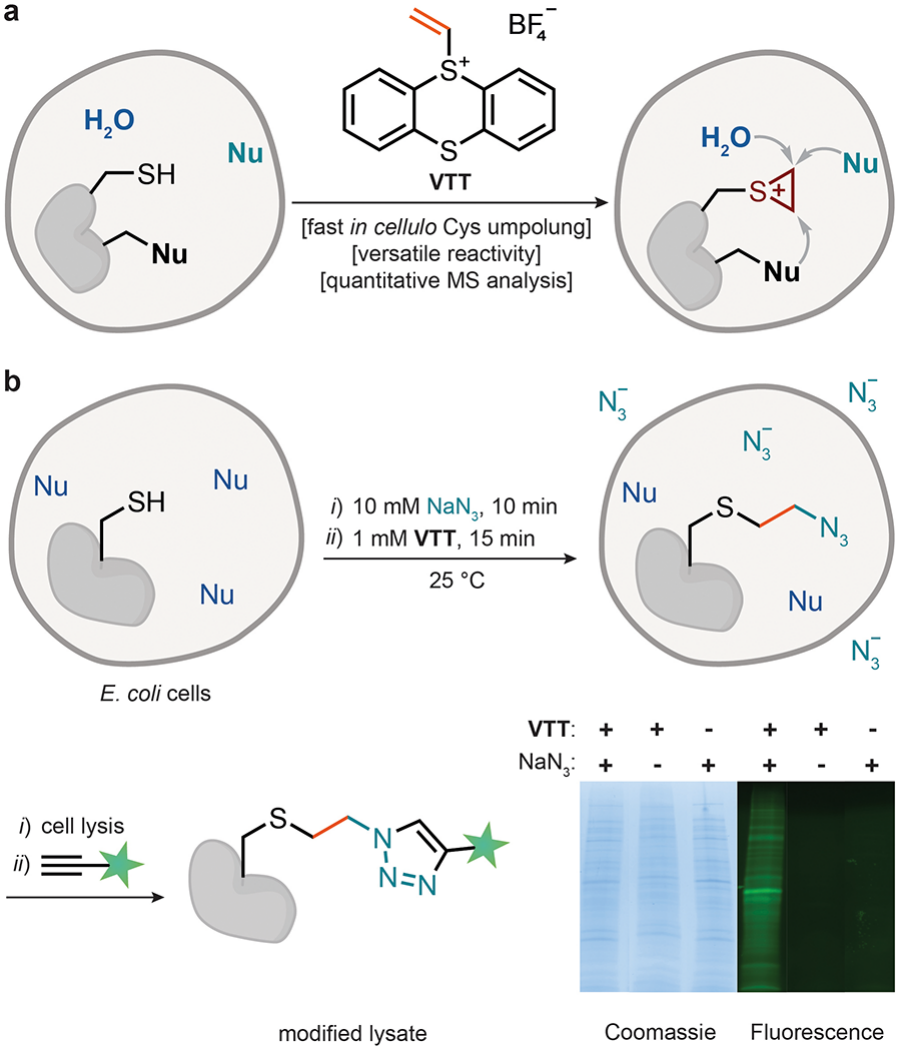
Intracellular cysteine umpolung with **VTT**. (a) Intracellular episulfonium generation via cysteine umpolung with **VTT**. (b) **VTT**-mediated incorporation of exogenous azide labels in *E. coli* cells and SDS-PAGE analysis of the respective cell lysates after CuAAC reaction to incorporate a fluorescein-based fluorescence marker.

## Results and Discussion

We have previously established that **VTT** can be used for a Cys-selective labeling in lysates with high Cys coverage.^4^ We hypothesized that the putative cell permeability of **VTT** could extend the strategy into a cellular context and enable episulfonium formation *in cellulo*. This would allow for the capturing of cell-permeable exogenous nucleophiles added to the growth medium. We incubated *E. coli* cells in the presence of 10 mM NaN_3_ for 15 min, followed by incubation with **VTT**. The cells were washed, lysed, and the putative alkyl azide-substituted fragments in the obtained lysates were then linked to a fluorescein tag via a copper-catalyzed azide–alkyne cycloaddition (CuAAC) reaction (Figure 1b). The sodium dodecyl sulfate-polyacrylamide gel electrophoresis (SDS-PAGE) analysis of the modified lysate indicates a proteome-wide incorporation of the azide group only when the cells were exposed to both **VTT** and NaN_3_. LC–MS/MS analysis of the digested samples further revealed that products originating from nucleophilic ring-opening by either water or azide can be identified in all samples treated with **VTT** (see Supporting Information, Figures S29 and S30). The experiment shows that **VTT** can penetrate cells, form electrophilic episulfonium intermediates on protein surfaces, and selectively trap exogenous nucleophiles *in cellulo*. The use of sodium azide further demonstrates that the formed organic azides can be readily diversified to obtain readouts for downstream analysis.

The cellular membrane poses a challenge for protein modifications in cells, and slow cellular uptake can therefore become the rate-determining step of the overall labeling process. To evaluate the efficiency of **VTT** for *in cellulo* protein labeling, we performed competition experiments between **VTT** and **NEM**, as well as between **VTT** and **IAA** alkyne, to determine the kinetic profile of the overall modification process (see Figure 2). Maleimides are among the reagents with the highest second-order rate constants known for cysteine labeling and are typically employed if fast cysteine alkylation is required,^20^ whereas iodoacetamide-based reagents such as **IAA** alkyne are considered the gold standard in cysteine conjugation and have been used in various proteome-wide studies.^8,21,22^

**Figure 2:**
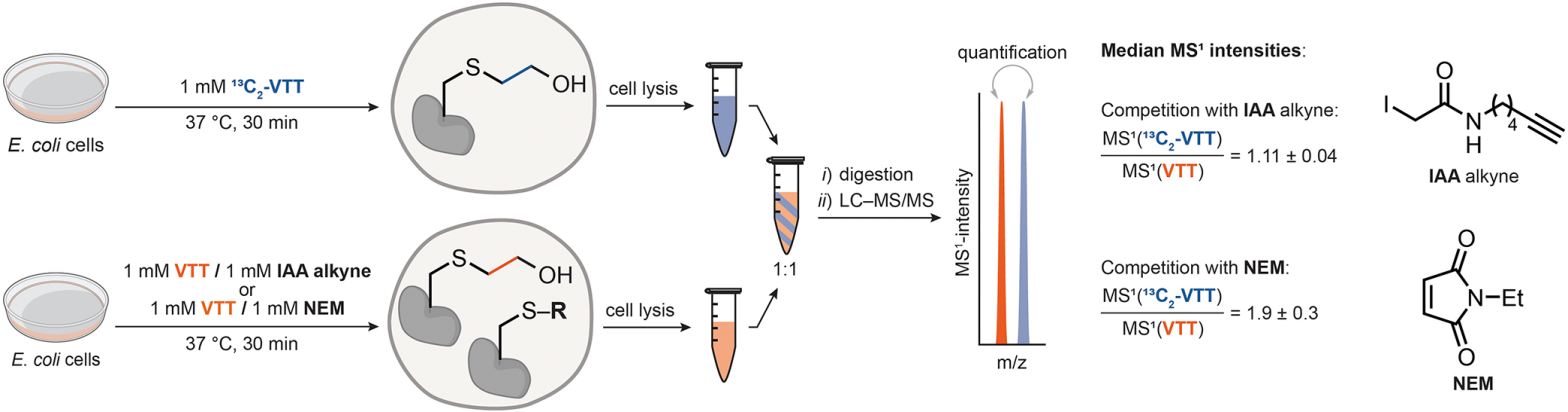
Competition experiments between **VTT** and **IAA** alkyne or **NEM**, respectively, in *E. coli* cells. S–R, Cys residues that were alkylated with **IAA** alkyne or **NEM**, respectively. The obtained median ratios are shown as mean ± SE of *n* = 3 independent experiments. Adapted from OpenScienceArt. Petri Dish, openscienceart.com/item/petri-dish-free-illustration.

Because quantitative and proteome-wide analysis of the labeling efficiency is desired, we employed the isotope-coded affinity tagging strategy.^23^ Isotopologues of **VTT** can be readily accessed from isotope-labeled ethylene gas to provide means of performing quantitative proteomics studies.^4^ We used the **^13^C_2_-** **VTT** isotopologue for treatment of *E. coli* cells in the absence of competing electrophiles to obtain the highest labeling degree achievable within 30 min of incubation time. In the competition experiment, **VTT** was added as an equimolar mixture with **IAA** alkyne or **NEM**, respectively, and the cells were incubated for 30 min at 37 °C, washed, and lysed. The LC–MS/MS analysis of 1:1 mixtures of the **IAA** alkyne/**VTT** lysate with the ^**13**^ **C**_**2**_ **-VTT** control lysate revealed a ratio of 1.11 ± 0.04 calculated from distinct MS^1^ intensities caused by the altered isotopic composition of the formed products. This result indicates no decrease in the level of **VTT**-mediated labeling caused by the competing **IAA** alkyne. In the competition experiment with **NEM**, the observed heavy-to-light ratio was 1.9 ± 0.3 and is in line with similar rates for **VTT** and **NEM** for the rate-determining step of the intracellular labeling process (Figure 2).

The same rate constant for labeling with **VTT** and **NEM** is noteworthy because **NEM** outcompetes **VTT** when the reaction is carried out *in vitro* (see the Supporting Information, Figure S4). Therefore, the data exclude the rate-determining Michael addition of the cysteinyl thiol and suggest the cell penetration of the electrophiles as the rate limiting step of the *in cellulo* modification process. To exclude the possibility that the fast cellular uptake of **VTT** is only restricted to bacterial cells, a competition reaction with **NEM** was conducted with mammalian K562 cells. The cells used in the experiment were preincubated with NaN_3_ prior to the addition of the reagents to enhance the sensitivity of the analysis. This approach enabled enrichment of the modified peptides based on the introduced azide moiety. After cell lysis, the protein concentrations in the samples were normalized, and the cell lysates labeled with the **VTT**-**NEM** mixture were combined in a 1:1 ratio with the control lysate that was labeled with ^**2**^ **H**_**3**_ **-VTT**. Next, a biotin moiety was introduced via CuAAC reaction, followed by tryptic digestion, and enrichment of the modified peptides via NeutrAvidin capturing. The analysis showed a ratio of 2.0 ± 0.1 for products originating from heavy-labeled ^**2**^ **H**_**3**_ **-VTT** compared to nonlabeled **VTT**. This result is in accordance with the data generated for *E. coli* and further supports similar rate constants for both **VTT** and **NEM** in the rate-limiting step of the transformation. The results demonstrate that **VTT** outcompetes **IAA** alkyne and provides similar labeling kinetics as **NEM**, one of the fastest cysteine-selective reagents in an *in cellulo* setup.^24^

To produce data that originate from proteins *in cellulo*, the utilized reagent must not lead to membrane damage in the cells. We performed a fluorometric cytotoxicity assay to exclude protein labeling outside of the cells and to evaluate potential membrane damage during the labeling reaction. The K562 cells were treated with the cell-impermeable CellTox dye that binds to dead cells’ DNA to produce an enhanced fluorescent signal. The cells were then treated with DMSO, **VTT**, or lysis buffer, and the fluorescence intensity was measured after 60 min of incubation at 37 °C. All concentrations as high as 1 mM in **VTT** did not cause any detectable membrane damage (Figure 3a), indicating the potential of the reagent for *in cellulo* studies and excluding extracellular labeling. Incubation with concentrations exceeding 1 mM **VTT** caused lysis of the treated mammalian cells and confirmed membrane degradation at such concentrations (Supporting Information, Figures S7 and S8).

**Figure 3:**
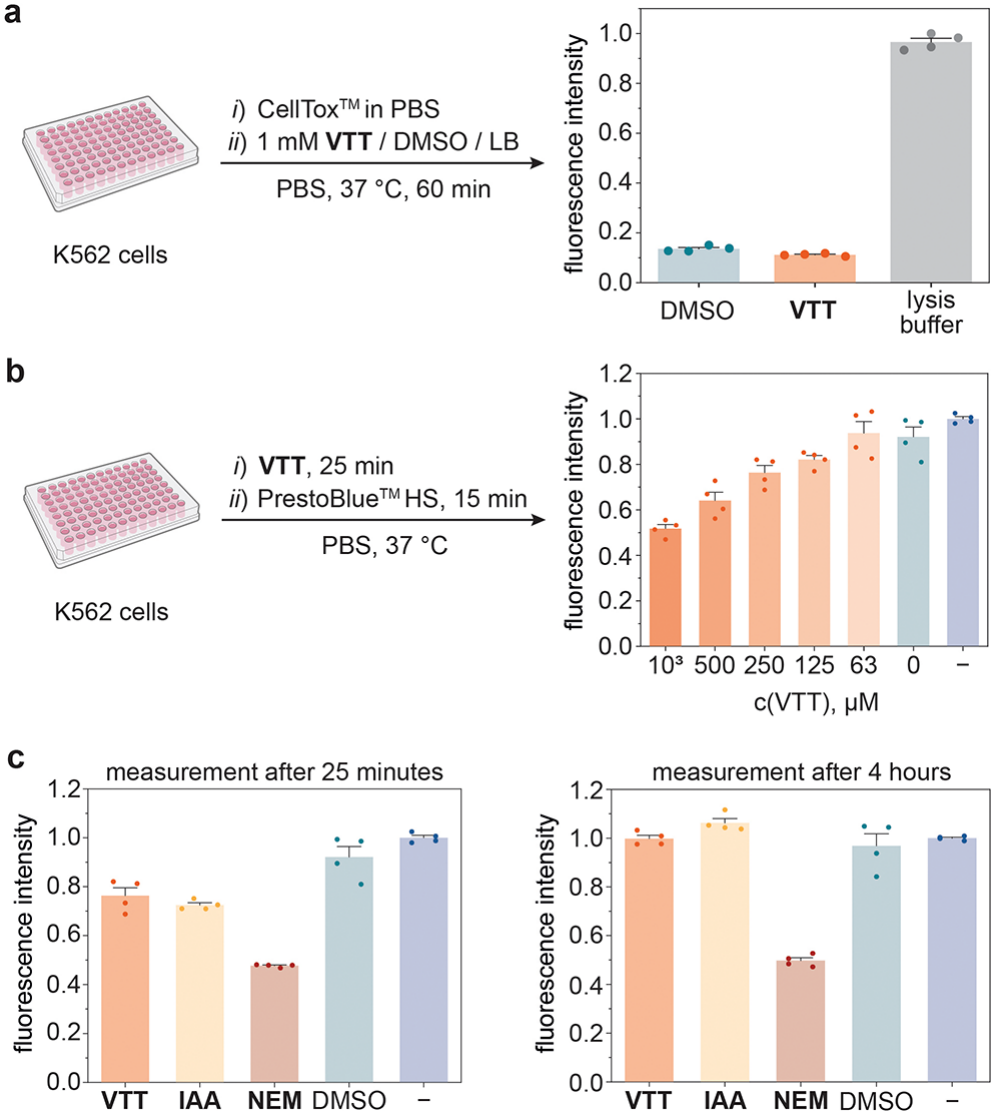
Cytotoxicity (membrane integrity) and cell viability (redox stress) assays. (a) Normalized fluorescence intensity values from CellTox membrane integrity assay performed with K562 cells after incubation with **VTT**, DMSO (1%, v/v), or lysis buffer (LB). Data are means ± SE of *n* = 4 independent experiments. Adapted from NIAID Visual & Medical Arts. 96 Well Plate, bioart.niaid.nih.gov/bioart/7. (b) Resazurin-based cell viability (redox stress) assay performed with K562 cells after incubation with **VTT** at various concentrations, DMSO (1%, v/v), or PBS (−) for 25 min. Data are means ± SE of *n* = 4 independent experiments. (c) Resazurin-based cell viability (redox stress) assay performed with K562 cells after incubation with 250 μM **VTT**, **NEM**, **IAA** alkyne **(IAA**), DMSO (1%, v/v), or PBS (−) for 25 min (left) or 4 h (right). Data are means ± SE of *n* = 4 independent experiments.

To further investigate the influence of the reagent on cellular metabolism, we conducted an additional assay that requires metabolically active cells for the intracellular reduction of nonfluorescent resazurin into fluorescent resorufin.^25^ The assay revealed decreased oxidoreductase activity at concentrations higher than 63 μM (Figure 3b). At 1 mM **VTT**, the cells still possessed 52 ± 2% of their initial metabolic activity. A further increase in fluorescence, measured 4 h after the initial reading, revealed sustained metabolic activity and indicated the presence of viable cells even with prolonged treatment times. To compare **VTT** with other commonly used alkylating reagents, we repeated the same experiment with **IAA** alkyne and **NEM** as representative thiol-selective probes. While treatment with 0.25 mM **IAA** alkyne caused a similar decline in metabolic activity to 72 ± 1% as induced by treatment with 0.25 mM **VTT** (76 ± 3%), treatment with **NEM** at the same concentration caused a stronger decrease to only 47.7 ± 0.3% of the initial activity (Figure 3c, left). Furthermore, samples treated with 0.25 mM or higher concentrations of **NEM** showed a 25% lower increase in fluorescence compared to **IAA** alkyne or **VTT**, as determined after 4 h of incubation with resazurin (Figure 3c, right). This result indicates a higher toxicity of **NEM** compared to **VTT** and **IAA** alkyne. Despite its detrimental effect on intracellular redox homeostasis, the fast and nondisruptive reactivity of **VTT** allows for *in cellulo* labeling of cysteine residues within minutes and ensures similar or higher levels of intracellular metabolism compared to the reagents commonly used for proteome-wide cysteine studies.

We then determined the number of modified peptides identified after the treatment of various cell types with **VTT** to evaluate the general applicability of **VTT** for *in cellulo* cysteine umpolung. A diverse set of different cell types including mammalian, bacterial, fungal, and plant cells were treated with **VTT**, digested, and analyzed by LC–MS/MS. The analysis revealed that labeling efficiency in the range of 19–28%, with an average of 24%, was achieved across all studied cell types (Figure 4). This result demonstrates the broad applicability of **VTT** for cysteine umpolung in various cell types. To compare the efficiency of the *in cellulo* labeling to an *in vitro* modification of lysates, we treated mammalian K562 cells and lysates with **VTT** under identical conditions. The numbers of modified peptides identified in both experiments are of the same order of magnitude and therefore indicate that similar labeling efficiency can be achieved even in the presence of the cell membrane in the *in cellulo* setup (see the Supporting Information, Figures S27 and S28). The gene-ontology analysis revealed that **VTT** modified proteins in all major cell compartments (see the Supporting Information, Figure S23). It is noteworthy that the reaction proceeds despite the high concentration of intracellular glutathione (GSH) that possesses a reactive thiol group and has an important role in the intracellular redox homeostasis.^26,27^ We hypothesize that the efficient labeling can be explained by a fast cellular uptake, the available excess of the reagent in relation to glutathione and other reduced cysteinyl thiols, and higher nucleophilicity of certain protein thiols compared to glutathione^8^ (see Supporting Information, page S9).

**Figure 4:**
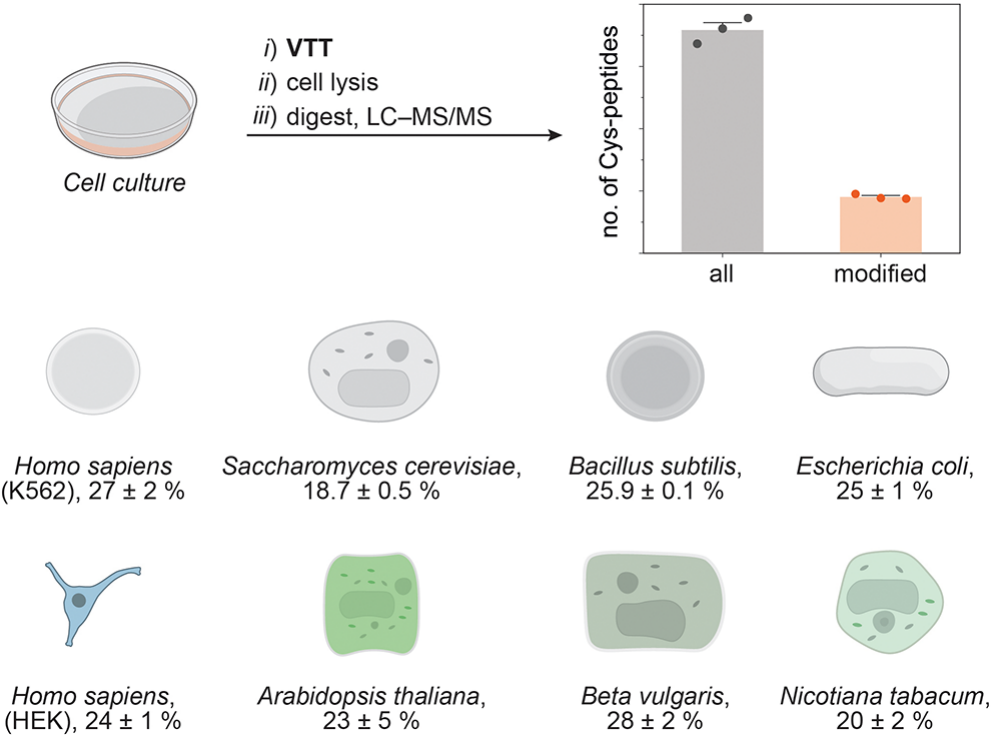
Evaluation of the labeling efficiency after *in cellulo* protein modification with **VTT** in various cell types. The modified (Cys–S–C_2_H_4_OH) and Cys-containing peptides were detected via LC–MS/MS. The values are means ± SE of *n* = 3 independent experiments. Adapted from OpenScienceArt. Petri Dish, openscienceart.com/item/petri-dish-free-illustration.

To rationalize the fast cellular uptake of **VTT**, we calculated the topological polar surface area (TPSA) and lipophilicity index (logP) for **VTT** and other Cys-reactive cross-linking reagents. Low calculated TPSA and high logP values have been correlated to high cell permeability and can be used to estimate the relative cell-permeability of compounds.^28,29^ The calculated TPSA of 50.6 Å^2^ and logP of 4.64 are in line with the fast cell permeability of **VTT** and compare favorably to the values obtained for other Cys-selective cross-linking reagents (see Supporting Information, Figure S41).

The episulfonium intermediates are efficient electrophilic traps for different oxygen-, sulfur-, and nitrogen-based nucleophiles that give short ethylene linkages between the sulfur atom of cysteine and the nucleophile. Generation of episulfonium ions on protein surfaces could therefore serve as an alternative to the currently applied photo-cross-linkers. We have previously demonstrated that episulfoniums can be used to form cross-links with Cys, Lys, and Glu.^4^ We hypothesized that other nucleophilic amino acid side chains should also be able to open the episulfonium intermediate to form various cross-links on protein surfaces. To determine the applicability of **VTT** for protein structure probing, we first established the reactivity profile of the episulfonium with all canonical nucleophilic amino acid side chains. The lysate samples obtained from the K562 cells after incubation with **VTT** were digested and analyzed by cross-linking LC–MS/MS. The cross-link search revealed cross-links between Cys and all nucleophilic amino acids including Cys, Asp, Glu, His, Lys, Arg, Ser, Thr, and Tyr (Figure 5a). While it is unsurprising that nucleophilic side chains like Cys can open the episulfonium ion, the fact that even hindered alcohols like Thr react in an aqueous environment is noteworthy. When the amino acid frequency within the identified cross-links is compared to the relative frequency in the proteome, the analysis reveals that amino acids with an oxygen- or sulfur-centered nucleophilic group react more frequently than amino acids with nitrogen-centered nucleophiles (see Supporting Information, Figures S32 and S33). One exception is the imidazole moiety of histidine residues. This effect is likely caused by the average p*K*_a_ value of 6.6,^30^ rendering it nucleophilic under the physiological pH value of 7.0–7.4 in intact cells.^31^ The results are in accordance with the short half-life and high reactivity of the formed episulfonium intermediate that reacts with all amino acids that possess a nucleophilic functional group at the intracellular pH value.

**Figure 5:**
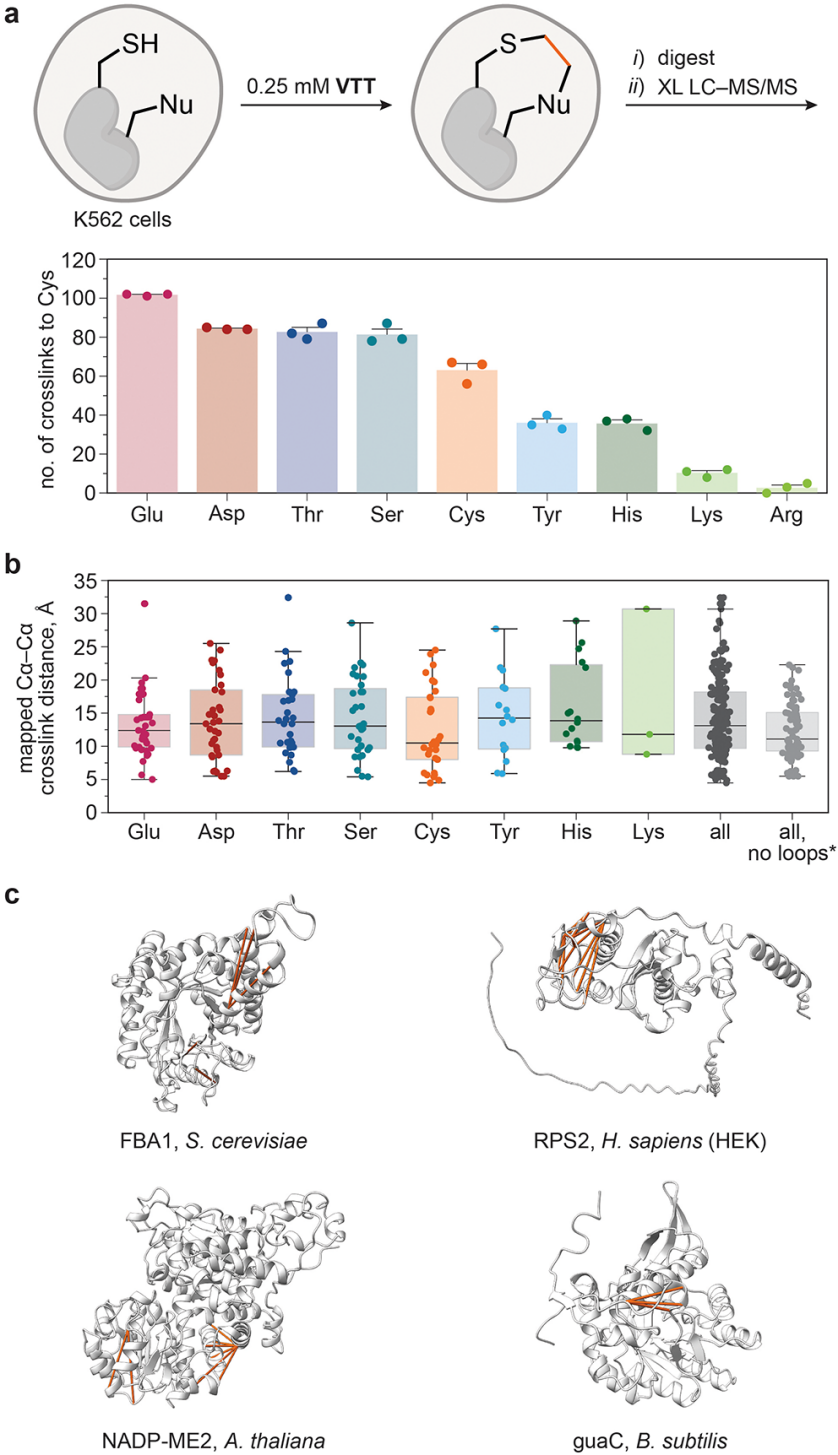
*In cellulo* intramolecular cross-linking with **VTT**. (a) The number of the detected cross-links from Cys to each of the searched amino acids detected after incubation of K562 cells with **VTT**. Data are means ± SE of *n* = 3 independent experiments. (b) Distribution of Cα-Cα distances for the mapped cross-links that were identified in at least two biological replicates. Each box spans from the first quartile (the 25th percentile) to the third quartile (the 75th percentile). Horizontal line indicates the median, whiskers extend to 1.5 × interquartile range from box boundaries. *Data were obtained after excluding mapped cross-links in nonordered protein structural motifs. (c) Representative proteins and detected cross-links from different organisms. The protein structures were predicted using AlphaLink2 ^32^ and visualized with ChimeraX.^33^

To evaluate and quantify the distance restraints obtained by cross-linking with **VTT**, cross-links that were detected in at least two biological replicates were mapped to high-resolution X-ray structures or high-quality AlphaFold^34^ predictions. The obtained distances between the two carbon atoms in the amide backbone (Cα–Cα) of the cross-linked side chains revealed that the episulfonium was most commonly trapped by amino acids that were 8–16 Å away from the Cys residue (see Supporting Information, Figure S37). The determined median Cα–Cα distance is in the range from 10 to 14 Å for each amino acid and therefore consistent with fast episulfonium reactivity with the residues in close proximity (Figure 5b). The median distance of 13.5 Å for all cross-linked amino acids is only slightly above the previously reported median distance of 11.1 Å for photoleucine-mediated cross-linking^2^ that requires the incorporation of an unnatural amino acid and shorter than the Cα–Cα cross-link distances of 15.9 Å obtained for Lys-selective disuccinimidyl sulfoxide cross-linker.^2^ To achieve greater accuracy in the cross-linking analysis of **VTT**, loops and other disordered protein structural motifs were excluded from the cross-linking data set, leaving only well-resolved and ordered motifs within the analyzed protein structures. This data set revealed a maximum Cα–Cα cross-link distance of 22.3 Å and a shift in the median distance to 11.1 Å (see Supporting Information, Figure S38; Figure 5b). The results demonstrate the broad applicability of episulfonium intermediate as a reactive electrophilic trap that can engage all nucleophilic amino acids to yield cross-links with distances comparable to the state-of-the-art photo-cross-linking methods and shorter than data obtained with lysine cross-linkers. Cross-linking with **VTT** can be performed in mammalian, bacterial, fungal, and plant cells (Figure 5c), providing a general and versatile strategy for an *in cellulo* protein structure probing.

MS analysis of cross-linking experiments often suffers from low signal intensity in samples obtained from *in cellulo* experiments and from the absence of cross-link peaks originating from proteins with low abundance.^35^ To extend our cross-linking protocol to naturally low-abundant proteins, we overexpressed the model protein glutaredoxin GrxC5 from *A. thaliana* in *E. coli* cells because we were not able to detect the protein within the full lysate *A. thaliana* sample. *In cellulo* protein cross-linking is preferred over cross-linking of purified proteins, as the cross-linking conditions should be as close as possible to the native environment.^36,37^ The cells with the overexpressed protein were incubated with **VTT** and washed prior to lysis, and the protein was separated by affinity chromatography. To further enhance the sensitivity and to prevent false-positive cross-links between homodimers, we isolated the monomer protein band via SDS-PAGE, performed an in-gel trypsin digest, and analyzed the samples by cross-linking LC–MS/MS. The analysis revealed four cross-links that were detected in two out of three biological replicates (Figure 6). These results demonstrate the potential of **VTT** for the extraction of geometrical restraints from low abundant proteins of interest in an *in cellulo* setup using basic molecular biology techniques.

**Figure 6:**
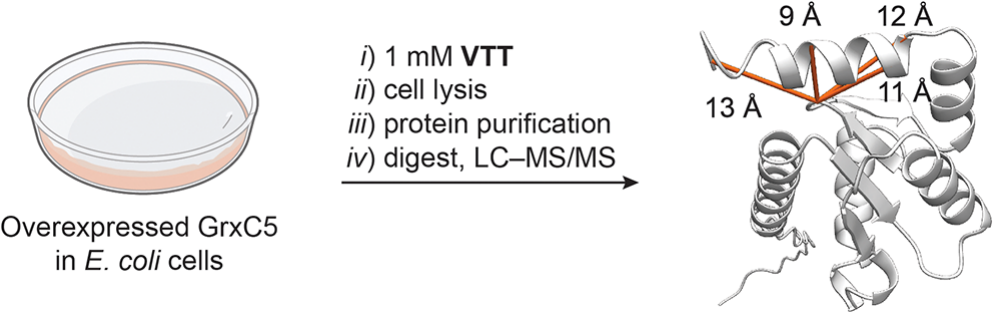
*In cellulo* cross-linking of overexpressed GrxC5 and detected cross-links. The protein structure was predicted using AlphaLink2 ^32^ and visualized with ChimeraX.^33^ Adapted from OpenScienceArt. Petri Dish, openscienceart.com/item/petri-dish-free-illustration.

Table 1 illustrates the comparison between **VTT** and other reagents used for chemical cross-linking. Generation of reactive carbene intermediates can be achieved after the initial protein modification with maleimide or *N*-hydroxysuccinimide esters that carry a diazirine group. However, these bifunctional reagents generate longer distance restraints than **VTT** and require exogenous UV-light activation in a separate step. Homobifunctional reagents such as disuccinimidyl glutarate (**DSG**) do not rely on exogenous activation, but form intermediates with sufficiently long half-lives to capture artificial protein conformations.^13^ Cross-linking with **VTT** introduces a short and stable ethylene linker via formation of a reactive intermediate and cross-linking in a single step. Moreover, isotopologues of **VTT** are accessible in one step from commercially available reagents and can be used for quantitative MS studies.

**Table 1:**
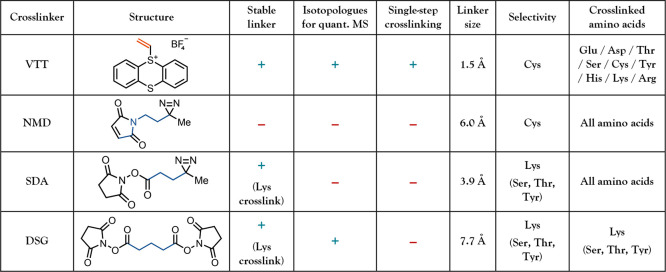
Comparison of **VTT**-Mediated Crosslinking to Other Approaches^a^

a **NMD**, *N*-maleimidodiazirine;^38^ **SDA**, succinimidyl azipentanoate;^39^ **DSG**, disuccinimidyl glutarate.^40^

## Conclusions

We have established a fast *in cellulo* protein labeling strategy via cysteine umpolung with commercially available **VTT**. The rapid cellular uptake ensures full membrane integrity for the treatment of mammalian cells at concentrations up to 1 mM, while achieving similar or higher cell viability compared to commonly used cysteine alkylating reagents. The generation of electrophilic episulfonium intermediates enables formation of short cross-links between Cys and native nucleophilic amino acids in various cell types. Furthermore, our strategy does not require unnatural amino acids, and sites of interest can simply be equipped with a cysteine residue to probe for the distance restraints in proteins of interest. We believe that the versatile chemistry of the episulfonium intermediates as well as easy synthetic access to the labeled vinylthianthrenium reagents will provide means for quantitative LC–MS/MS studies *in cellulo*. Subsequent chemical diversification of the **VTT** core structure and incorporation of an enrichment tag may expand the areas of application toward intracellular interactome studies.

## Supplementary Material



## Notes

Experimental procedures, analytical methods, and spectroscopic data (PDF)

## References

[1] Yang Z., Zeng X., Zhao Y., Chen R. (2023). AlphaFold2 And Its
Applications in the Fields of Biology And Medicine. Signal Transduction and Targeted Therapy.

[2] Stahl K., Graziadei A., Dau T., Brock O., Rappsilber J. (2023). Protein Structure
Prediction With In-cell Photo-crosslinking Mass Spectrometry and Deep
Learning. Nat. Biotechnol..

[3] Suchanek M., Radzikowska A., Thiele C. (2005). Photo-leucine and Photo-methionine
Allow Identification of Protein-protein Interactions in Living Cells. Nat. Methods.

[4] Hartmann P., Bohdan K., Hommrich M., Juliá F., Vogelsang L., Eirich J., Zangl R., Farès C., Jacobs J. B., Mukhopadhyay D., Mengeler J. M., Vetere A., Sterling M. S., Hinrichs H., Becker S., Morgner N., Schrader W., Finkemeier I., Dietz K.-J., Griesinger C., Ritter T. (2024). Chemoselective Umpolung of Thiols to Episulfoniums
for Cysteine Bioconjugation. Nat. Chem..

[5] Spradlin J. N., Zhang E., Nomura D. K. (2021). Reimagining Druggability Using Chemoproteomic
Platforms. Acc. Chem. Res..

[6] Backus K. M., Correia B. E., Lum K. M., Forli S., Horning B. D., González-Páez G. E., Chatterjee S., Lanning B. R., Teijaro J. R., Olson A. J., Wolan D. W., Cravatt B. F. (2016). Proteome-wide Covalent Ligand Discovery
in Native Biological
Systems. Nature.

[7] Abbasov M. E., Kavanagh M. E., Ichu T.-A., Lazear M. R., Tao Y., Crowley V. M., am Ende C. W., Hacker S. M., Ho J., Dix M. M., Suciu R., Hayward M. M., Kiessling L. L., Cravatt B. F. (2021). A Proteome-wide
Atlas of Lysine-Reactive chemistry. Nat. Chem..

[8] Weerapana E., Wang C., Simon G. M., Richter F., Khare S., Dillon M. B. D., Bachovchin D. A., Mowen K., Baker D., Cravatt B. F. (2010). Quantitative Reactivity Profiling Predicts Functional
Cysteines in Proteomes. Nature.

[9] Graziadei A., Rappsilber J. (2022). Leveraging
Crosslinking Mass Spectrometry in Structural
and Cell Biology. Structure.

[10] Chen Z. A., Rappsilber J. (2023). Protein Structure
Dynamics by Crosslinking Mass Spectrometry. Curr. Opin. Struct. Biol..

[11] Steigenberger B., Albanese P., Heck A. J. R., Scheltema R. A. (2020). To Cleave
or Not To Cleave in XL-MS?. J. Am. Soc. Mass
Spectrom..

[12] Belsom A., Rappsilber J. (2021). Anatomy of a Crosslinker. Curr.
Opin. Chem. Biol..

[13] Fabris D., Yu E. T. (2010). Elucidating the higher-order structure
of biopolymers by structural
probing and mass spectrometry: MS3D. Journal
of Mass Spectrometry.

[14] Leitner A., Walzthoeni T., Kahraman A., Herzog F., Rinner O., Beck M., Aebersold R. (2010). Probing Native Protein Structures
by Chemical Cross-linking, Mass Spectrometry, and Bioinformatics. Molecular & Cellular Proteomics.

[15] Merkley E. D., Rysavy S., Kahraman A., Hafen R. P., Daggett V., Adkins J. N. (2014). Distance Restraints
From Crosslinking Mass Spectrometry:
Mining a Molecular Dynamics Simulation Database to Evaluate Lysine-Lysine
Distances. Protein Sci..

[16] Kohl B., Brüderlin M., Ritz D., Schmidt A., Hiller S. (2020). Protocol for
High-Yield Production of Photo-Leucine-Labeled Proteins in Escherichia
coli. J. Proteome Res..

[17] Jiang Y., Zhang X., Nie H., Fan J., Di S., Fu H., Zhang X., Wang L., Tang C. (2024). Dissecting Diazirine
Photo-reaction Mechanism for Protein Residue-Specific Cross-Linking
and Distance Mapping. Nat. Commun..

[18] Juliá F., Yan J., Paulus F., Ritter T. (2021). Vinyl Thianthrenium Tetrafluoroborate:
A Practical and Versatile Vinylating Reagent Made from Ethylene. J. Am. Chem. Soc..

[19] Liu J., Powell K. L., Thames H. D., MacLeod M. C. (2010). Detoxication of
Sulfur Half-Mustards by Nucleophilic Scavengers: Robust Activity of
Thiopurines. Chem. Res. Toxicol..

[20] McConnell E. W., Smythers A. L., Hicks L. M. (2020). Maleimide-Based
Chemical Proteomics
for Quantitative Analysis of Cysteine Reactivity. J. Am. Soc. Mass Spectrom..

[21] Wang C., Weerapana E., Blewett M. M., Cravatt B. F. (2014). A chemoproteomic
platform to quantitatively map targets of lipid-derived electrophiles. Nat. Methods.

[22] Maurais A. J., Weerapana E. (2019). Reactive-cysteine profiling for drug discovery. Curr. Opin. Chem. Biol..

[23] Gygi S. P., Rist B., Gerber S. A., Turecek F., Gelb M. H., Aebersold R. (1999). Quantitative Analysis of Complex
Protein Mixtures Using
Isotope-coded Affinity Tags. Nat. Biotechnol..

[24] Schnitzer J. E., Allard J., Oh P. (1995). NEM inhibits transcytosis, endocytosis,
and capillary permeability: implication of caveolae fusion in endothelia. American Journal of Physiology-Heart and Circulatory Physiology.

[25] Anoopkumar-Dukie S., Carey J. B., Conere T., O’Sullivan E., van Pelt F. N., Allshire A. (2005). Resazurin assay of radiation response
in cultured cells. British Journal of Radiology.

[26] Georgiou-Siafis S. K., Samiotaki M. K., Demopoulos V. J., Panayotou G., Tsiftsoglou A. S. (2022). Glutathione-Hemin/Hematin Adduct Formation to Disintegrate
Cytotoxic Oxidant Hemin/Hematin in Human K562 Cells and Red Blood
Cells’ Hemolysates: Impact of Glutathione on the Hemolytic
Disorders and Homeostasis. Antioxidants.

[27] Gamcsik M. P., Kasibhatla M. S., Teeter S. D., Colvin O. M. (2012). Glutathione Levels
in Human Tumors. Biomarkers.

[28] Pajouhesh H., Lenz G. R. (2005). Medicinal chemical
properties of successful central
nervous system drugs. NeuroRX.

[29] Benet L. Z., Hosey C. M., Ursu O., Oprea T. I. (2016). BDDCS, the Rule
of 5 and drugability. Adv. Drug Delivery Rev..

[30] Edgcomb S. P., Murphy K. P. (2002). Variability in the
pKa of histidine side-chains correlates
with burial within proteins. Proteins: Struct.,
Funct., Bioinf..

[31] Madshus I. H. (1988). Regulation
of intracellular pH in eukaryotic cells. Biochem.
J..

[32] Stahl K., Warneke R., Demann L., Bremenkamp R., Hormes B., Brock O., Stülke J., Rappsilber J. (2024). Modelling Protein Complexes With Crosslinking Mass
Spectrometry and Deep Learning. Nat. Commun..

[33] Pettersen E. F., Goddard T. D., Huang C. C., Couch G. S., Greenblatt D. M., Meng E. C., Ferrin T. E. (2004). UCSF Chimera—A
Visualization
System for Exploratory Research and Analysis. J. Comput. Chem..

[34] Jumper J., Evans R., Pritzel A., Green T., Figurnov M., Ronneberger O., Tunyasuvunakool K., Bates R., Žídek A., Potapenko A., Bridgland A., Meyer C., Kohl S. A. A., Ballard A. J., Cowie A., Romera-Paredes B., Nikolov S., Jain R., Adler J., Back T., Petersen S., Reiman D., Clancy E., Zielinski M., Steinegger M., Pacholska M., Berghammer T., Bodenstein S., Silver D., Vinyals O., Senior A. W., Kavukcuoglu K., Kohli P., Hassabis D. (2021). Highly Accurate Protein
Structure Prediction With AlphaFold. Nature.

[35] Fürsch J., Kammer K.-M., Kreft S. G., Beck M., Stengel F. (2020). Proteome-Wide
Structural Probing of Low-Abundant Protein Interactions by Cross-Linking
Mass Spectrometry. Anal. Chem..

[36] Chavez J. D., Mohr J. P., Mathay M., Zhong X., Keller A., Bruce J. E. (2019). Systems structural biology measurements by in vivo
cross-linking with mass spectrometry. Nat. Protoc..

[37] Zhang B., Gong Z., Zhao L., An Y., Gao H., Chen J., Liang Z., Liu M., Zhang Y., Zhao Q., Zhang L. (2023). Decoding Protein Dynamics in Cells
Using Chemical Cross-Linking and Hierarchical Analysis. Angew. Chem., Int. Ed..

[38] Walko M., Hewitt E., Radford S. E., Wilson A. J. (2019). Design and synthesis
of cysteine-specific labels for photo-crosslinking studies. RSC Adv..

[39] Raphel J., Parisi-Amon A., Heilshorn S. C. (2012). Photoreactive elastin-like proteins
for use as versatile bioactive materials and surface coatings. J. Mater. Chem..

[40] Leitner A., Walzthoeni T., Aebersold R. (2014). Lysine-specific chemical cross-linking
of protein complexes and identification of cross-linking sites using
LC-MS/MS and the xQuest/xProphet software pipeline. Nat. Protoc..

