# Supplementary material for: In Cellulo Cysteine Umpolung for Protein Structure Probing
Source: J Am Chem Soc. 2025 Sep 19;147(39):35587–94. doi: 10.1021/jacs.5c10259 (PMC12498408; doi:10.1021/jacs.5c10259)
Supplement: Supplementary file 1 [file ja5c10259_si_001.pdf]

SUPPORTING INFORMATION

In cellulo cysteine umpolung for protein structure probing

Philipp Hartmann[1,2,‡], Kostiantyn Bohdan[1,2,‡], Lara Vogelsang[3], Dario Marchionni[1], Christian Preisinger[4], Alessandro Vetere[1], Karl-Josef Dietz[3], and Tobias Ritter[*,1]

[1]Max-Planck-Institut für Kohlenforschung, Kaiser-Wilhelm-Platz 1, 45470 Mülheim an der Ruhr, Germany.

[2]Institute of Organic Chemistry, RWTH Aachen University, Landoltweg 1, 52074 Aachen, Germany.

[3]Biochemistry and Physiology of Plants, Faculty of Biology, Bielefeld University, Universitätsstraße 25, 33615 Bielefeld, Germany.

[4]Proteomics Facility, Interdisciplinary Centre for Clinical Research (IZKF), RWTH Aachen University, 52074 Aachen, Germany.

[‡]These authors contributed equally.

*E-mail: ritter@kofo.mpg.de

TABLE OF CONTENTS

TABLE OF CONTENTS 1

ABBREVIATIONS 3

MATERIALS AND METHODS 4

Starting Materials 4

NMR Spectroscopy 4

LC–MS/MS 4

SDS PAGE 7

Miscellaneous 7

EXPERIMENTAL DATA 9

In cellulo protein labeling 9

K562 cells 9

HEK cells 9

Arabidopsis thaliana 10

Bacillus subtilis 10

Beta vulgaris 11

Saccharomyces cerevisiae 11

Nicotiana tabacum 12

Escherichia coli 12

In vitro protein labeling 14

Generation of K562 lysates 14

Labeling of K562 lysates with **VTT** 14

Competition experiments 15

In cellulo competition experiment with *E.coli* cells 15

In cellulo competition experiment with K562 cells 16

In vitro competition experiment 18

Sample preparation for LC–MS/MS measurements 21

SP3 protocol 21

Protocol for biotin enrichment 21

In cellulo protein modification in *E. coli* cells for SDS-PAGE analysis 23

Membrane integrity studies 26

K562 cells 26

HEK cells 26

Influence of **VTT** concentration on protein recoveries 27

Viability studies 28

Assay with a delayed addition of resazurin reagent 28

Assay with direct addition of resazurin reagent 32

In cellulo crosslinking of GrxC5 36

Plasmid generation and amplification 36

In cellulo crosslinking with **VTT** and protein purification 36

In-gel protein digestion 37

DATA ANALYSIS 38

Quantification of labeling efficiency 38

Modification search 38

Labeling efficiencies 38

Gene ontology analysis 39

Quantitative LC–MS/MS analysis 41

Competition experiments with *E. coli* cells 41

Competition experiments with K562 cells 43

Intersection analysis of identified peptides 44

Labeling of K562 cells and K562 lysates 44

Labeling of *E. coli* cells with and without sodium azide 45

Crosslink searches 46

In cellulo crosslinking with K562 cells 46

In cellulo crosslinking with *S. cerevisiae* cells 57

In cellulo crosslinking with HEK cells 58

In cellulo crosslinking with *A. thaliana* cells 58

In cellulo crosslinking with *B. subtilis* cells 59

In cellulo crosslinked GrxC5 60

Crosslinker comparison 61

Crosslinking reagents used for comparison 61

Calculated TPSA and logP 61

Avogardo outputs 63

Data availability 67

REFERENCES 68

ABBREVIATIONS

**Abbreviation**	**Full Name**
BCA	Bicinchoninic Acid
CuAAC	Copper-catalyzed azide-alkyne cycloaddition
DMEM	Dulbecco's Modified Eagle Medium
DMF	*N*,*N*-Dimethylformamide
DMSO	Dimethyl Sulfoxide
DPBS	Dulbecco's Phosphate-Buffered Saline
DTT	Dithiothreitol
EDTA	Ethylenediaminetetraacetic acid
6-FAM	6-Carboxyfluorescein
FCS	Fetal Calf Serum
FDR	False discovery rate
FWHM	Full Width at Half Maximum
HCD	Higher Energy C Trap Dissociation
IAA	Iodoacetamide
LB	Luria-Bertani (Medium)
MQ	Milli-Q
MS	Murashige and Skoog (Medium)
NEM	*N*-Ethylmaleimide
NP-40	Nonidet P-40
PBS	Phosphate-Buffered Saline
PEG	Polyethylene Glycol
PMSF	Phenylmethylsulfonyl Fluoride
RIPA	Radioimmunoprecipitation Assay buffer
RPMI	Roswell Park Memorial Institute (Medium)
SDS	Sodium Dodecyl Sulfate
SP3	Single-pot, solid-phase-enhanced sample preparation
TCEP	Tris(2-carboxyethyl)phosphine
THPTA	Tris(3-hydroxypropyltriazolylmethyl)amine
TPSA	Topological polar surface area
TSP	3-(Trimethylsilyl)propionic-2,2,3,3-d4 Acid Sodium Salt
VTFT	Tetrafluorovinylthianthrenium tetrafluoroborate
VTT	Vinylthianthrenium tetrafluoroborate
YPD	Yeast Extract Peptone Dextrose (Medium)

MATERIALS AND METHODS

Starting Materials

Reagents

VTT, ^{13}**C**$_2$**-VTT**, ^{2}**H**$_3$**-VTT**, and **VTFT** were synthesized as described previously[1, 2]. Biotin-PEG3-alkyne was purchased from BroadPharm (#BP-40761). *N*-ethylmaleimide was purchased from ThermoFischer (#156100050). *N*-hex-5-ynyl-2-iodoacetamide (**IAA** alkyne) was purchased from BLD Pharmatech (#BD324491). 6-FAM alkyne was purchased from Lumiprobe (#C51B0). THPTA was purchased from TCI Chemicals (#T3171). TCEP•HCl was purchased from Carl Roth (#HN95.1). DTT was purchased from Chempur (#GC3928).

Solvents

Water used to prepare buffers and as a solvent was of ultra-high quality (UHQ) grade (18.2 MΩ·cm^{-1}). Dimethylformamide was purchased from Thermo Scientific (>99.8%). Acetonitrile (>99%) and dimethyl sulfoxide (>99%) were purchased from Fisher Scientific.

NMR Spectroscopy

Chemical shifts are reported in ppm (δ) relative to tetramethylsilane (TMS) with the solvent (residual) peak as the internal standard (HDO, δ 4.79). The spectra were obtained on AVANCE III 600 spectrometer equipped with a triple-channel “TCI” cryogenic probehead (Bruker GmbH, Rheinstetten) operating at 600 MHz for ^{1}H-acquisitions. All experiments used standard Bruker pulse sequence with standard parameter sets found in libraries of Topspin 3.6.

LC–MS/MS

LC–MS/MS Method A

Acquisition of mass spectra was performed on an Orbitrap Eclipse Tribrid Mass Spectrometer (Thermo Fisher Scientific) connected to a Vanquish Neo UHPLC system (Thermo Fisher Scientific). The samples were dissolved in 0.1% (v/v) aqueous formic acid, loaded onto a trapping column (Acclaim PepMap100, C18, 5 µm, 100 Å, 300 µm i.d. × 5 mm, Thermo Scientific) using a loading volume of 5 µL, and eluted onto the analytical column in reverse direction. Separation was achieved on a PepMap Neo capillary column (C-18, 50 cm, 75 µm internal diameter, 2 µm particle size, 100 Å pore size, Thermo Fisher Scientific). The peptide separation was performed with a binary mobile phase gradient using 0.1% (v/v) aqueous formic acid as mobile phase A and 0.1% (v/v) formic acid in acetonitrile as mobile phase B at a flow rate of 300 nL min^{-1}: 0–103 min, 1.5→24% B; 103–128 min, 24→32% B; 128–135 min, 32→8% B; and 135–139 min, 8→90% B. Electrospray ionization was achieved with a Nanospray Flex ESI source equipped with a stainless steel emitter.

The MS data were acquired in data-dependent mode with varying cycle settings. In each case, the full-scan mass spectrum was recorded in the Orbitrap at a resolution of R = 60,000 (FWHM at m/z 200) in the range of

m/z 375 to 1500. Ions with a precursor charge state between +2 and +5 were isolated using a 2 Da isolation window and fragmented using Higher-energy collisional dissociation at a normalized energy level of 30%. Dynamic exclusion was enabled after two repeats with 30 s exclusion duration.

Fragment spectra of intensity ordered parent ions (charge state +2 to +5) were recorded in the linear ion trap over a cycle time of 2 s using rapid resolution setting. A Real-Time-Search was employed, searching for modified target peptides against the corresponding FASTA database for the respective organism. Identified hits were analyzed using an additional MS^2 scan in the Orbitrap at a resolution of R = 15,000 (FWHM at m/z 200).

LC–MS/MS Method B

Acquisition of mass spectra was performed on an Orbitrap Eclipse Tribrid Mass Spectrometer (Thermo Fisher Scientific) connected to a Vanquish Neo UHPLC system (Thermo Fisher Scientific). The samples were dissolved in 0.1% (v/v) aqueous formic acid, loaded onto a trapping column (Acclaim PepMap100, C18, 5 µm, 100 Å, 300 µm i.d. × 5 mm, Thermo Scientific) using a loading volume of 5 µL, and eluted onto the analytical column in reverse direction. Separation was achieved on a PepMap Neo capillary column (C-18, 50 cm, 75 µm internal diameter, 2 µm particle size, 100 Å pore size, Thermo Fisher Scientific). The peptide separation was performed with a binary mobile phase gradient using 0.1% (v/v) aqueous formic acid as mobile phase A and 0.1% (v/v) formic acid in acetonitrile as mobile phase B at a flow rate of 300 nL min^{-1}: 0–103 min, 1.5→24% B; 103–128 min, 24→32% B; 128–135 min, 32→8% B; and 135–139 min, 8→90% B. Electrospray ionization was achieved with a Nanospray Flex ESI source equipped with a stainless steel emitter.

The MS data were acquired in data-dependent mode with varying cycle settings. In each case, the full-scan mass spectrum was recorded in the Orbitrap at a resolution of R = 60,000 (FWHM at m/z 200) in the range of m/z 375 to 1500. Ions with a precursor charge state between +2 and +5 were isolated using a 2 Da isolation window and fragmented using Higher-energy collisional dissociation at a normalized energy level of 30%. Dynamic exclusion was enabled after two repeats with 30 s exclusion duration.

Fragment spectra of the 20 most intense parent ions (charge state +2 to +5) from the full scan spectrum were recorded in the Orbitrap at a resolution of R = 15,000 (FWHM at m/z 200).

LC–MS/MS Method C

Acquisition of mass spectra was performed on an Orbitrap Eclipse Tribrid Mass Spectrometer (Thermo Fisher Scientific) connected to a Vanquish Neo UHPLC system (Thermo Fisher Scientific). The samples were dissolved in 0.1% (v/v) aqueous formic acid, loaded onto a trapping column (Acclaim PepMap100, C18, 5 µm, 100 Å, 300 µm i.d. × 5 mm, Thermo Scientific) using a loading volume of 5 µL, and eluted onto the analytical column in reverse direction. Separation was achieved on a PepMap Neo capillary column (C-18, 50 cm, 75 µm internal diameter, 2 µm particle size, 100 Å pore size, Thermo Fisher Scientific). The peptide separation was performed with a binary mobile phase gradient using 0.1% (v/v) aqueous formic acid as mobile phase A and 0.1% (v/v) formic acid in acetonitrile as mobile phase B at a flow rate of 300 nL min^{-1}: 0–

103 min, 1.5→24% B; 103–128 min, 24→32% B; 128–135 min, 32→8% B; and 135–139 min, 8→90% B. Electrospray ionization was achieved with a Nanospray Flex ESI source equipped with a stainless steel emitter.

The MS data were acquired in data-dependent mode with varying cycle settings. In each case, the full-scan mass spectrum was recorded in the Orbitrap at a resolution of R = 60,000 (FWHM at m/z 200) in the range of m/z 375 to 1500. Ions with a precursor charge state between +3 and +8 were isolated using a 2 Da isolation window and fragmented using higher-energy collisional dissociation at a normalized energy level of 30%. Dynamic exclusion was enabled after two repeats with 30 s exclusion duration.

Fragment spectra of the 10 most intense parent ions (charge state +3 to +8) from the full scan spectrum were recorded in the Orbitrap at a resolution of R = 15,000 (FWHM at m/z 200).

LC–MS/MS Method D

Acquisition of mass spectra was performed on an Exploris 480 mass spectrometer (Thermo Scientific) coupled to an RSLCnano UHPLC system (Thermo Scientific). The samples were dissolved in 3% (v/v) formic acid / 1% (v/v) acetonitrile, loaded onto a trapping column (Acclaim PepMap100, C18, 5 µm, 100 Å, 300 µm i.d. × 5 mm, Thermo Scientific) over 10 min, and then eluted onto the analytical column. Separation was achieved on an Aurora Ultimate column (C18, 25 cm, 75 µm internal diameter, IonOpticks) maintained at 45 °C. The peptide separation was performed with a binary mobile phase gradient using 0.1% (v/v) aqueous formic acid as mobile phase A and 80% (v/v) acetonitrile / 0.1% (v/v) formic acid as mobile phase B with a flow rate of 300 nL min^{-1}: 0–2 min, 2% B; 2–105 min, 2→30% B; 105–130 min, 30→40% B; 130–137 min, 40→99% B; 137–142 min, 99% B; 142–145 min, 95→2% B; and 145–160 min, 2% B. Electrospray ionization was achieved with a 2 kV spray voltage and a capillary temperature of 275 °C.

MS data were acquired in data-dependent mode based on the previously reported settings[3]. The MS^1 spectra were recorded over m/z 375–1500 at a resolution of 60,000 with an automated gain control (AGC) of 300% and a minimum intensity threshold of 5×10^3. Precursors with charge states from +3 to +8 were selected for fragmentation with dynamic exclusion set to automatic. Fragment spectra of the top 10 precursors were acquired at a resolution of 30,000, using a 1 Da isolation window, normalized stepped HCD collision energies of 21%, 27%, and 33%, normalized AGC of 100%, and a maximum injection time of 64 ms.

LC–MS/MS Method E

Acquisition of mass spectra was performed on an Exploris 480 mass spectrometer (Thermo Scientific) coupled to an RSLCnano UHPLC system (Thermo Scientific). The samples were dissolved in 3% (v/v) formic acid / 1% (v/v) acetonitrile, loaded onto a trapping column (Acclaim PepMap100, C18, 5 µm, 100 Å, 300 µm i.d. × 5 mm, Thermo Scientific) over 10 min, and then eluted onto the analytical column. Separation was achieved on an Aurora Ultimate column (C18, 25 cm, 75 µm internal diameter, IonOpticks) maintained at 45 °C. The peptide separation was performed with a binary mobile phase gradient using 0.1% (v/v) aqueous formic acid as mobile phase A and 80% (v/v) acetonitrile / 0.1% (v/v) formic acid as mobile phase B with a flow rate of 300 nL min^{-1}: 0–2 min, 2% B; 2–105 min, 2→30% B; 105–130 min, 30→40% B; 130–137 min,

40→99% B; 137–142 min, 99% B; 142–145 min, 95→2% B; and 145–160 min, 2% B. Electrospray ionization was achieved with a 2 kV spray voltage and a capillary temperature of 275 °C.

MS data were acquired in data-dependent mode. The MS^1 spectra were recorded over m/z 350–1250 at a resolution of 60,000 with an automated gain control (AGC) set to standard and a minimum intensity threshold of 8×10^3. Precursors with charge states from +2 to +5 were selected for fragmentation with dynamic exclusion set to automatic. Fragment spectra of the top 20 precursors were acquired at a resolution of 15,000, using a 2 m/z isolation window, normalized stepped HCD collision energy of 30%, normalized AGC of 100%, and a maximum injection time set to auto.

SDS PAGE

SDS-PAGE sample preparation and analysis was carried out as instructed in the manual of Bio-Rad[4]. Fluorescence measurements were carried out as instructed in the manual of Bio-Rad utilizing the "Fluorescein" analysis of the Bio-Rad Laboratories ChemiDocMP system. Coomassie Blue staining of the gels was performed as instructed by the manufacturer. Fixation was performed for 15 min and Coomassie Blue staining was performed for 20 h. All proteins were analyzed with Tris/Glycine/SDS gel electrophoresis.

Image Lab Version 6.1.0 build 7 software from Bio-Rad Laboratories Inc. was used for analysis and processing of SDS-PAGE data.

Reagents used in the SDS-PAGE workflow:

Bio-Rad Laboratories Any kD MP TGX Stain-Free 10W 30 µL

Bio-Rad Laboratories 10x Tris/Glycine/SDS buffer

Bio-Rad Laboratories 4x Laemmli sample buffer

Bio-Rad Laboratories QC Colloidal Coomassie stain

Bio-Rad Laboratories Precision Plus Protein™ Dual Xtra Prestained Protein Standards

Devices used in the SDS-PAGE workflow:

Bio-Rad Laboratories ChemiDocMP

Bio-Rad Laboratories Mini-PROTEAN Tetra Cell

Bio-Rad Laboratories PowerPac Basic

Miscellaneous

Reactions were conducted in an Eppendorf ThermoMixer® C equipped with an Eppendorf SmartBlock™ 1.5 mL. Sample preparation for LC–MS/MS analysis was performed in Protein LoBind® tubes from Eppendorf. Protein rebuffering was performed with AMICON® filters units from Sigma Aldrich. Lysate and protein concentrations were determined via BCA assay (ThermoFisher, #23225) using a Thermo Scientific™ NanoDrop™ One^{C}. Fluorescence measurements were performed on Plate reader SpectraMax iD3 (Molecular

Devices) and the results were analyzed with Soft Max Pro® 7 software. The Countess 3 from Invitrogen was used for determination of the number of mammalian cells. Cell lysis via sonication was performed with BANDELIN Sonoplus HD 2070 with Sonotrode MS 73. Precellys Evolution system from Bertin Technologies was used for cell homogenization.

Unless stated otherwise, Milli-Q water (MQ-H_2O) was used in all experiments.

EXPERIMENTAL DATA

In cellulo protein labeling

Unless stated otherwise, all experiments were performed with 3 biological replicates.

K562 cells

9 mL of K562 cells (1.4×10^6 cells / mL) in 1x Gibco RPMI 1640 Medium supplemented with 10% of heat-inactivated FCS was centrifuged for 5 min at 1,500 xg at 4 °C in a 15 mL Falcon tube, the supernatant was removed, the cells were resuspended in 20 mL of DPBS, centrifuged for 5 min at 1,500 xg at 4 °C, the supernatant was discarded, the cells were washed again by resuspending with 20 mL of DPBS and centrifuging for 5 min at 1,500 xg at 4 °C, and the supernatant was discarded. The cell pellet was resuspended in 3 mL of DPBS and transferred to a 6-well culture plate (1 mL suspension / experiment). To each well, 10 µL of a 25 mM stock solution of **VTT** in DMSO was added within 1 s and the cells were incubated for 30 min at 37 °C in 5 % CO_2 in a humidified atmosphere. Next, 10 µL of freshly prepared 25 mM DTT stock solution in H_2O was added and the cells were incubated for another 15 min at 37 °C.

Afterwards, 272 µL of RIPA lysis buffer was added to obtain final concentrations of 1% NP-40, 0.5% SDS, 0.5% sodium deoxycholate, and Tris (pH 8.0, 50 mM), followed by addition of 12.3 µL of protease inhibitor stock solution (Protease Inhibitor Mix M from Serva) in DMSO. The mixtures were mixed by pipetting and incubated at 4 °C for 30 min. Then, the mixtures were transferred to 1.5 mL tubes, centrifuged for 15 min at 4 °C at 21,000 xg. The mixtures were frozen and kept at –78 °C until further processing.

For LC–MS/MS sample preparation, 0.1 µL of 5 kU Pierce™ nuclease stock solution was added to the thawed samples, the mixtures were incubated at 25 °C for 15 min at 500 rpm, centrifuged for 15 min at 21,000 xg at 4 °C, the supernatants were transferred to separate tubes and protein concentration was determined via BCA assay. The protein digest was performed according to the SP3 protocol and analyzed via LC–MS/MS using method A for the modification search or method D for the crosslink search.

Based on the previously reported intracellular thiol concentration in K562 cells (230 pmol / 1×10^6 cells) [5], the thiol amount in each experiment with 4.2×10^6 cells was $< 1\times10^{-9}$ mol, whereas the amount of the added **VTT** was 250×10^{-9} mol.

HEK cells

Confluent (~95%) HEK cells in 1x Gibco DMEM + GlutaMAX Medium supplemented with 10% of heat-inactivated FCS in a 6-well (9.6 cm^2) culture plate were washed 2 times with 2 mL of DPBS and 1 mL of DPBS was added. Next, 10 µL of 25 mM stock solution of **VTT** in DMSO was added within 1 s and the cells were incubated for 30 min at 37 °C in 5 % CO_2 in a humidified atmosphere. Next, 10 µL of freshly prepared 25 mM DTT stock solution in H_2O was added and the cells were incubated for another 15 min at 37 °C.

Afterwards, 272 µL of RIPA lysis buffer was added to obtain final concentrations of 1% NP-40, 0.5% SDS, 0.5% sodium deoxycholate, and Tris (pH 8.0, 50 mM), followed by addition of 12.3 µL of protease inhibitor stock solution (Protease Inhibitor Mix M from Serva) in DMSO. The mixtures were mixed by

pipetting and incubated at 4 °C for 30 min. Then, the mixtures were transferred to 1.5 mL tubes, centrifuged for 15 min at 4 °C at 21,000 xg. The mixtures were frozen and kept at –78 °C until further processing.

For LC–MS/MS sample preparation, 0.1 µL of 5 kU Pierce™ nuclease stock solution was added to the thawed samples, the mixtures were incubated at 25 °C for 15 min at 500 rpm, centrifuged for 15 min at 21,000 xg at 4 °C, the supernatants were transferred to separate tubes and protein concentration was determined via BCA assay. The protein digest was performed according to the SP3 protocol and analyzed via LC–MS/MS using method A for the modification search or method D for the crosslink search.

Arabidopsis thaliana

3 mL of an *Arabidopsis thaliana* cell culture in MS-Medium (4.3 g/L MS salts (Sigma M5524); 1.0 mg/L 2,4-dichlorophenoxyacetic acid; 1:1000 (v/v) 1000×Gamborg's vitamin stock (Sigma G1019); 30 g/L sucrose; pH 5.7, adjusted with KOH) that was incubated for 7 days after cell passage were transferred into an autoclaved Erlenmeyer flask. Next, 30 µL of a 0.10 M **VTT** solution (c_{final} = 1.0 mM) or 30 µL of a 0.50 M **VTT** solution (c_{final} = 5.0 mM) in DMSO was added within 1 s and the cells were incubated under gentle agitation at 26 °C for 60 min. Afterwards, the cells were transferred into a centrifugation tube and centrifuged for 30 min with 4,600 xg at 4 °C. Next, the supernatant was discarded and the cells were resuspended in 8 mL HEPES (pH 7.0, 50 mM) buffer. The suspension was again centrifuged for 10 min with 4,600 xg at 4 °C. The supernatant was discarded, and the cells were frozen in liquid nitrogen and stored until further use. For complete lysis, the frozen pellet was resuspended in 750 µL HEPES (pH 7.0, 50 mM) buffer and homogenized in a Precellys system with 7,500 rpm, 3 × 50 s with 50 s breaks. Next, the lysates were centrifuged for 20 min with 20,000 xg at 4 °C and the clear supernatant was transferred into a new 1.5 mL reaction tube. The lysates were frozen in liquid nitrogen and stored at –78 °C for later LC–MS/MS sample preparation.

For LC–MS/MS sample preparation, the thawed samples were centrifuged for 15 min at 21,000 xg at 4 °C, the supernatants were transferred to separate tubes and protein concentration was determined via BCA assay. The protein digest was performed according to the SP3 protocol and analyzed via LC–MS/MS using method A for the modification search (samples treated with 1.0 mM and 5.0 mM **VTT**) or method D for the crosslink search (samples treated with 5.0 mM **VTT**).

Bacillus subtilis

3 mL of a *Bacillus subtilis* cell culture in Miller-LB medium with an OD_{600}-value of 1.9 were transferred into an autoclaved Erlenmeyer flask. Next, 30 µL of 0.10 M **VTT** solution in DMSO (c_{final} = 1.0 mM) was added within 1 s and the cells were incubated under gentle agitation at 37 °C for 30 min. Afterwards, the cells were transferred into a centrifugation tube and centrifuged for 30 min with 4,600 xg at 4°C. Then, the supernatant was discarded and the cells were resuspended in 5 mL HEPES (pH 7.0, 50 mM) buffer. The suspension was again centrifuged for 10 min with 4,600 xg at 4°C. The supernatant was discarded and the cells were frozen in liquid nitrogen and stored until lysis. For complete lysis, the frozen pellet was resuspended in 750 µL HEPES (pH 7.0, 50 mM) buffer. Next, the mixture was sonicated 2 × 20 s with 70 % power at 20-25 °C and subsequently 5 min with 100% power in an ice bath. Afterwards, the tube was centrifuged for 15 min with 21,000 xg at 4 °C and the clear supernatant was transferred into a new

1.5 mL reaction tube. The lysate was frozen in liquid nitrogen and stored at –78 °C for later LC–MS/MS sample preparation.

For LC–MS/MS sample preparation, the thawed samples were centrifuged for 15 min at 21,000 xg at 4 °C, the supernatants were transferred to separate tubes, and protein concentration was determined via BCA assay. The protein digest was performed according to the SP3 protocol and analyzed via LC–MS/MS using method A for the modification search or method D for the crosslink search.

Beta vulgaris

3 mL of a *Beta vulgaris* cell culture in MS-Medium (4.3 g/L MS salts (Sigma M5524); 120 g/L sucrose; 1.0 µg/L 1-naphthalene acetic acid; 0.5 µg/L 6-benzyladenine; 2.0 µg/L vitamin C; pH 5.8, adjusted with KOH) that was incubated for 7 days after cell passage were transferred into an autoclaved Erlenmeyer flask. Next, 30 µL of 0.10 M **VTT** solution in DMSO (c_{final} = 1.0 mM) was added within 1 s and the cells were incubated under gentle agitation at 26 °C for 60 min. Afterwards, the cells were transferred into a centrifugation tube and centrifuged for 30 min with 4,600 xg at 4°C. The cells did not pellet but remained in the supernatant after the centrifugation. The supernatant with the cells was carefully removed (approximately 2–3 mL), and the cells were resuspended in 6 mL of HEPES buffer (pH 7.0, 50 mM). The suspension was centrifuged for 30 min with 4,600 xg at 4°C, after which a pellet formed. The cells were resuspended in 8 mL HEPES (pH 7.0, 50 mM) buffer. The suspension was again centrifuged for 10 min with 4,600 xg at 4°C. The supernatant was discarded and the cells were frozen in liquid nitrogen and stored until further use. For complete lysis, the frozen pellet was resuspended in 750 µL HEPES (pH 7.0, 50 mM) buffer and homogenized in a Precellys system with 7,500 rpm, 3 × 50 s with 50 s breaks. Next, the lysates were centrifuged for 20 min with 20,000 xg at 4 °C and the clear supernatant was transferred into a new 1.5 mL reaction tube. The lysates were frozen in liquid nitrogen and stored at –78 °C for later LC–MS/MS sample preparation.

For LC–MS/MS sample preparation, the thawed samples were centrifuged for 15 min at 21,000 xg at 4 °C, the supernatants were transferred to separate tubes and protein concentration was determined via BCA assay. The protein digest was performed according to the SP3 protocol and analyzed via LC–MS/MS using method A.

Saccharomyces cerevisiae

3 mL of a *Saccharomyces cerevisiae* cell culture (BY4742, MATα; his3Δ1; leu2Δ0; lys2Δ0; ura3Δ0) with an OD_{600}-value of 2.9 in YPD-medium were transferred into an autoclaved Erlenmeyer flask. Next, 30 µL of a 0.10 M **VTT** solution (c_{final} = 1.0 mM) or 30 µL of a 0.50 M **VTT** solution (c_{final} = 5.0 mM) in DMSO was added within 1 s and the cells were incubated under gentle agitation at 28 °C for 60 min. Afterwards, the cells were transferred into a centrifugation tube and centrifuged for 30 min with 4,600 xg at 4°C. Afterwards, the supernatant was discarded and the cells were resuspended in 5 mL HEPES (pH 7.0, 50 mM) buffer. The suspension was again centrifuged for 10 min with 4,600 xg at 4°C. The supernatant was discarded and the cells were frozen in liquid nitrogen and stored until further use. For complete lysis, the frozen pellet was resuspended in 750 µL HEPES (pH 7.0, 50 mM) buffer and homogenized in a Precellys system with 7,500 rpm, 3 × 50 s with 50 s breaks. Next, the lysates were centrifuged for 20 min with 20,000 xg at 4 °C and the clear supernatant was transferred into a new 1.5 mL reaction tube. The lysates were frozen in liquid nitrogen and stored at –78 °C for later LC–MS/MS sample preparation.

For LC–MS/MS sample preparation, the thawed samples were centrifuged for 15 min at 21,000 xg at 4 °C, the supernatants were transferred to separate tubes and protein concentration was determined via BCA assay. The protein digest was performed according to the SP3 protocol and analyzed via LC–MS/MS using method A for the modification search (samples treated with 1.0 mM and 5.0 mM **VTT**) or method D for the crosslink search (samples treated with 5.0 mM **VTT**).

Nicotiana tabacum

3 mL of a *Nicotiana tabacum* BY2 cell culture in MS-Medium (4.3 g/L MS salts (Sigma M5524); 30 g/L sucrose; 0.2 µg/L 2,4-dichlorophenoxyacetic acid; 0.56 mM myo-inositol; 3.0 µM thiamine; 1.9 mM KH_2PO_4; pH 5.0, adjusted with KOH) that was incubated for 7 days after cell passage were transferred into an autoclaved Erlenmeyer flask. Next, 30 µL of a 0.10 M **VTT** solution (c_{final} = 1.0 mM) or 30 µL of a 0.50 M **VTT** solution (c_{final} = 5.0 mM) in DMSO was added within 1 s and the cells were incubated under gentle agitation at 26 °C for 60 min. Afterwards, the cells were transferred into a centrifugation tube and centrifuged for 30 min with 4,600 xg at 4°C. Afterwards, the supernatant was discarded and the cells were resuspended in 8 mL HEPES (pH 7.0, 50 mM) buffer. The suspension was again centrifuged for 10 min with 4,600 xg at 4°C. The supernatant was discarded and the cells were frozen in liquid nitrogen and stored until lysis. For complete lysis, the frozen pellet was resuspended in 750 µL HEPES (pH 7.0, 50 mM). The cells were lysed in a Precellys system with 7,500 rpm, 3 × 50 s with 50 s breaks. Next, the lysates were centrifuged for 20 min with 20,000 xg at 4 °C and the clear supernatant was transferred into a new 1.5 mL reaction tube. The lysates were frozen in liquid nitrogen and stored at –78 °C for later LC–MS/MS sample preparation.

For LC–MS/MS sample preparation, the thawed samples were centrifuged for 15 min at 21,000 xg at 4 °C, the supernatants were transferred to separate tubes and protein concentration was determined via BCA assay. The protein digest was performed according to the SP3 protocol and analyzed via LC–MS/MS using method A.

Escherichia coli

3 mL of an *E. coli* cell culture in Miller-LB medium with an OD_{600}-value of 2.5 were transferred into an autoclaved Erlenmeyer flask. Next, 30 µL of 0.10 M **VTT** solution in DMSO (c_{final} = 1.0 mM) was added within 1 s and the cells were incubated under gentle agitation at 37 °C for 30 min. Afterwards, the cells were transferred into a centrifugation tube and centrifuged for 30 min with 4,600 xg at 4°C. Then, the supernatant was discarded and the cells were resuspended in 5 mL HEPES (pH 7.0, 50 mM) buffer. The cells were again centrifuged for 10 min with 4,600 xg at 4°C. The supernatant was discarded and the cells were frozen in liquid nitrogen and stored until lysis. For complete lysis, the frozen pellet was resuspended in 320 µL of NEB Express lysis reagent mix. The mixture was incubated for 1 h at 20–25 °C. Next, the lysate was centrifuged for 15 min with 21,000 xg at 20–25 °C and the clear supernatant was transferred into a new 1.5 mL reaction tube. The lysates were frozen in liquid nitrogen and stored at –78 °C for later LC–MS/MS sample preparation.

For LC–MS/MS sample preparation, the thawed samples were centrifuged for 15 min at 21,000 xg at 4 °C, the supernatants were transferred to separate tubes and protein concentration was determined via BCA assay. The protein digest was performed according to the SP3 protocol and analyzed via LC–MS/MS using method A.

Labeling with and without NaN_3

Two 10 mL aliquots of an *E. coli* cell culture in Miller-LB medium with an OD_{600}-value of 2.1 were transferred into separate autoclaved Erlenmeyer flasks. To one flask, 150 µL of 1.0 M sodium azide in H_2O (c_{final} = 15 mM) was added within 1 s, while the other was supplemented with 150 µL H_2O. Both cultures were incubated under gentle agitation at 37 °C for 10 min. Subsequently, 100 µL of 0.1 M VTT in DMSO (c_{final} = 1.0 mM) was rapidly added to each flask, followed by an additional incubation at 37 °C for 15 min with gentle shaking. After this, 100 µL of 0.1 M DTT in H_2O (c_{final} = 1.0 mM) was added and incubation was continued for an additional 15 min under identical conditions. Afterwards, the cell suspensions were transferred into a centrifugation tube and centrifuged for 20 min at 4,600 xg at 4°C. Then, the supernatant was discarded and the cells were resuspended in 5 mL HEPES (pH 7.0, 50 mM) buffer. The cells were again centrifuged for 10 min at 4,600 xg at 4°C. The supernatant was discarded and the washing step was repeated. The cell pellet was frozen in liquid nitrogen and stored until lysis. For complete lysis, the frozen pellet was resuspended in 800 µL of NEB Express lysis reagent mix. The mixture was incubated for 40 min at 20–25 °C. The lysates were then centrifuged for 10 min with 21,000 xg at 20–25 °C and the clear supernatant was transferred into a new 1.5 mL reaction tube. The lysates were frozen in liquid nitrogen and stored at –78 °C for later LC–MS/MS sample preparation. The entire procedure was performed in three independent biological replicates per condition.

For LC–MS/MS sample preparation, the thawed samples were centrifuged for 15 min at 21,000 xg at 4 °C, the supernatants were transferred to separate tubes and protein concentration was determined via BCA assay. The protein digest was performed according to the SP3 protocol and analyzed via LC–MS/MS using method A.

In vitro protein labeling

Generation of K562 lysates

K562 cells (1×10^6 cells / mL) were washed 2 times with DPBS buffer, the supernatant was discarded, the cells were split in tubes (50 µL each) and the pellets were frozen and stored at –78 °C. 150 µL of HEPES (pH 7.0, 50 mM) buffer containing 1 × cOmplete™, Mini, EDTA-free Protease Inhibitor Cocktail was added to thawed K562 cell pellets and resuspended via pipetting. The tubes were frozen in liquid nitrogen, and after 1 min transferred to a Thermocycler pre-cooled to 4 °C. The mixtures were incubated for 13 min at 300 rpm, vortexed for 3 s, and frozen in liquid nitrogen. The freeze-thaw cycle was repeated 2 more times, the tubes were centrifuged for 15 min at 21,000 xg at 4 °C. Then, the supernatant was collected, transferred to new tubes, and protein concentration was determined via BCA assay.

Labeling of K562 lysates with VTT

The experiment was performed with three biological replicates. In each case, 4.1 µL of the K562 lysate (12.3 mg/mL) was added to a tube containing 45 µL of PBS buffer to obtain final protein concentration of 1.0 mg/mL and the mixture was vortexed for 1 s. Then, 0.5 µL of a 0.25 mM **VTT** stock solution in DMSO was added to the mixture, vortexed for 1 s, and the tube was transferred to a Thermocycler preheated at 37 °C. The mixture was incubated for 30 min at 600 rpm and 0.5 µL of a 0.25 mM DTT stock solution in H_2O was added to the mixture. The mixture was incubated for 15 min at 37 °C at 600 rpm, centrifuged for 10 min at 21,000 xg at 4 °C, the supernatant was collected and transferred to a new tube for the LC–MS/MS sample preparation. The protein digest was performed according to the SP3 protocol and analyzed via LC–MS/MS using method A.

Competition experiments

In cellulo competition experiment with *E.coli* cells

Control experiments with $^{13}C_2$-VTT and VTT

For each replicate, 1.5 mL of an *E. coli* cell culture in Miller-LB medium with an OD_{600} of 3.1 was transferred into a 2 mL reaction tube. The cultures were supplemented with one of the following compound combinations to reach a final concentration of 1.0 mM per reagent: (a) 30 µL of 50 mM **VTT** in DMSO; (b) 30 µL of 50 mM **$^{13}C_2$-VTT** in DMSO. The mixtures were incubated under gentle agitation at 37 °C for 30 min. Next, the cells were transferred into a centrifugation tube and centrifuged for 30 min with 4,600 xg at 4°C. Then, the supernatants were discarded and the resulting pellets were resuspended in 2 mL HEPES buffer (pH 7.0, 50 mM). The cells were again centrifuged for 10 min with 4,600 x g at 4°C. After discarding the supernatant, the pellets were frozen in liquid nitrogen and stored at –78 °C until lysis. For cell lysis, the frozen pellets were resuspended in 150 µL of NEB Express lysis reagent mix and incubated for 30 min at room temperature (21 °C). Lysates were then centrifuged at 20–25 °C for 15 min at 21,000 xg, and the clear supernatants were transferred into fresh 1.5 mL reaction tubes. The lysates were frozen in liquid nitrogen and stored at –78 °C for later LC–MS/MS sample preparation. The entire procedure was performed in three independent biological replicates per condition.

Competition experiment with VTT and IAA-alkyne

For each replicate, 1.5 mL of an *E. coli* cell culture in Miller-LB medium with an OD_{600} of 3.1 was transferred into a 2 mL reaction tube. Next, 30 µL of a freshly prepared DMSO solution containing 0.1 M **VTT** and 0.1 M **IAA** was added to each mixture. The mixtures were incubated under gentle agitation at 37 °C for 30 min. Next, the cells were transferred into a centrifugation tube and centrifuged for 30 min with 4,600 xg at 4°C. Then, the supernatants were discarded and the resulting pellets were resuspended in 2 mL HEPES buffer (pH 7.0, 50 mM). The cells were again centrifuged for 10 min with 4,600 x g at 4°C. After discarding the supernatant, the pellets were frozen in liquid nitrogen and stored at –78 °C until lysis. For cell lysis, the frozen pellets were resuspended in 150 µL of NEB Express lysis reagent mix and incubated for 30 min at room temperature (21 °C). Lysates were then centrifuged at 20–25 °C for 15 min at 21,000 xg, and the clear supernatants were transferred into fresh 1.5 mL reaction tubes. The lysates were frozen in liquid nitrogen and stored at –78 °C for later LC–MS/MS sample preparation. The entire procedure was performed in three independent biological replicates per condition.

Competition experiment with $^{13}C_2$-VTT and IAA-alkyne

For each replicate, 1.5 mL of an *E. coli* cell culture in Miller-LB medium with an OD_{600} of 3.1 was transferred into a 2 mL reaction tube. Next, 30 µL of a freshly prepared DMSO solution containing 0.1 M **$^{13}C_2$-VTT** and 0.1 M **IAA** was added to each mixture. The mixtures were incubated under gentle agitation at 37 °C for 30 min. Next, the cells were transferred into a centrifugation tube and centrifuged for 30 min with 4,600 xg at 4°C. Then, the supernatants were discarded and the resulting pellets were resuspended in 2 mL HEPES buffer (pH 7.0, 50 mM). The cells were again centrifuged for 10 min with 4,600 x g at 4°C. After discarding the supernatant, the pellets were frozen in liquid nitrogen and stored at –78 °C until lysis. For cell lysis, the frozen pellets were resuspended in 150 µL of NEB Express lysis reagent mix and incubated for 30 min at room temperature (21 °C). Lysates were then centrifuged at 20–25 °C for 15 min at 21,000 xg, and the clear supernatants were transferred into fresh 1.5 mL reaction tubes. The lysates

were frozen in liquid nitrogen and stored at –78 °C for later LC–MS/MS sample preparation. The entire procedure was performed in three independent biological replicates per condition.

Competition experiment with VTT and NEM

For each replicate, 1.5 mL of an *E. coli* cell culture in Miller-LB medium with an OD_{600} of 3.1 was transferred into a 2 mL reaction tube. Next, 30 µL of a freshly prepared DMSO solution containing 0.1 M **VTT** and 0.1 M **NEM** was added to each mixture. The mixtures were incubated under gentle agitation at 37 °C for 30 min. Next, the cells were transferred into a centrifugation tube and centrifuged for 30 min with 4,600 xg at 4°C. Then, the supernatants were discarded and the resulting pellets were resuspended in 2 mL HEPES buffer (pH 7.0, 50 mM). The cells were again centrifuged for 10 min with 4,600 x g at 4°C. After discarding the supernatant, the pellets were frozen in liquid nitrogen and stored at –78 °C until lysis. For cell lysis, the frozen pellets were resuspended in 150 µL of NEB Express lysis reagent mix and incubated for 30 min at room temperature (21 °C). Lysates were then centrifuged at 20–25 °C for 15 min at 21,000 xg, and the clear supernatants were transferred into fresh 1.5 mL reaction tubes. The lysates were frozen in liquid nitrogen and stored at –78 °C for later LC–MS/MS sample preparation. The entire procedure was performed in three independent biological replicates per condition.

Sample preparation for LC–MS/MS analysis

The protein concentration of thawed samples was determined via BCA assay, each of the three lysates from the control experiments was combined with a lysate from the competition experiment as follows:

- **$^{13}C_2$-VTT** control experiment samples and samples from **VTT**/**IAA** competition experiment
- **VTT** control experiment samples and samples from **$^{13}C_2$-VTT**/**IAA** competition experiment
- **$^{13}C_2$-VTT** control experiment samples and samples from **VTT**/**NEM** competition experiment

The samples were digested according to the SP3 protocol and analyzed via LC–MS/MS using method E.

In cellulo competition experiment with K562 cells

32 mL of K562 cells (1.1×10^6 cells / mL) in 1x Gibco RPMI 1640 Medium supplemented with 10% of heat-inactivated FCS were centrifuged for 5 min at 1,500 xg at 4 °C in a 50 mL Falcon tube, the supernatant was removed, the cells were resuspended in 25 mL of DPBS, centrifuged for 5 min at 1,500 xg at 4 °C, the supernatant was discarded, the cells were washed again with 25 mL of DPBS and the supernatant was discarded. The cell pellet was resuspended in 30 mL of DPBS and transferred to petri dishes (5 mL suspension / experiment).

Control experiment with $^2H_3$-VTT

50 µL of a 1.5 M NaN_3 solution in DPBS was added to three dishes with K562 cells in DPBS, the dishes were gently swirled and incubated for 15 min at 37 °C in 5 % CO_2 in a humidified atmosphere. Then, 50 µL of a 0.1 M **VTT** stock solution in DMSO was added to each mixture, the dishes were gently swirled and incubated for 5 min at 37 °C in 5 % CO_2 in a humidified atmosphere. Next, 50 µL of freshly prepared 0.1 M DTT stock solution in H_2O was added and the mixtures and the cells were incubated for another 15 min at 37 °C.

Afterwards, 1.3 mL of RIPA lysis buffer was added to obtain final concentrations of 1% NP-40, 0.5% SDS, 0.5% sodium deoxycholate, and Tris (pH 8.0, 50 mM), followed by addition of 63 µL of protease inhibitor

stock solution (Protease Inhibitor Mix M from Serva) in DMSO. The mixtures were mixed by pipetting and incubated at 4 °C for 30 min. Then, the mixtures were transferred to 15 mL Falcon tubes, frozen and kept at –78 °C.

For the follow-up sample preparation, 0.5 µL of 5 kU Pierce™ nuclease stock solution was added to each of the thawed samples, the mixtures were incubated at 25 °C for 15 min and filtered 5 times with HEPES (pH 7.0, 50 mM) containing 1x cOmplete protease inhibitor with 10 kDa Amicon filters. The obtained mixtures were centrifuged for 15 min at 21,000 xg at 4 °C, the supernatant was transferred to new tubes and the protein concentration was determined via BCA assay.

Competition experiment with VTT and NEM

50 µL of a 1.5 M NaN_3 solution in DPBS was added to three dishes with K562 cells in DPBS. The dishes were gently swirled and incubated for 15 min at 37 °C in 5 % CO_2 in a humidified atmosphere. Then, 50 µL of a freshly prepared DMSO solution containing 0.1 M **VTT** and 0.1 M **NEM** was added to each mixture, the dishes were gently swirled and incubated for 5 min at 37 °C in 5 % CO_2 in a humidified atmosphere. Next, 50 µL of freshly prepared 0.1 M DTT stock solution in H_2O was added and the mixtures and the cells were incubated for another 15 min at 37 °C.

Afterwards, 1.3 mL of RIPA lysis buffer was added to obtain final concentrations of 1% NP-40, 0.5% SDS, 0.5% sodium deoxycholate, and Tris (pH 8.0, 50 mM), followed by addition of 63 µL of protease inhibitor stock solution (Protease Inhibitor Mix M from Serva) in DMSO. The mixtures were mixed by pipetting and incubated at 4 °C for 30 min. Then, the mixtures were transferred to 15 mL Falcon tubes, frozen and kept at –78 °C.

For the follow-up sample preparation, 0.5 µL of 5 kU Pierce™ nuclease stock solution was added to each of the thawed samples, the mixtures were incubated at 25 °C for 15 min and filtered 5 times with HEPES (pH 7.0, 50 mM) containing 1x cOmplete protease inhibitor with 10 kDa Amicon filters. The obtained mixtures were centrifuged for 15 min at 21,000 xg at 4 °C, the supernatant was transferred to new tubes and the protein concentration was determined via BCA assay.

Copper-catalyzed azide-alkyne cycloaddition (CuAAC) reaction for biotin enrichment

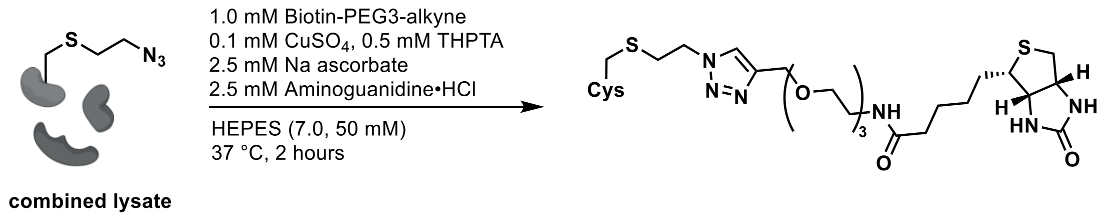

Each of the three filtered lysates from the control experiments with **$^2H_3$-VTT** (0.25 mg protein each) was combined with a filtered lysate from the competition experiment (0.25 mg protein each), respectively. HEPES (pH 7.0, 50 mM) was added to obtain the mixture volume of 470 µL in each case. Then, 13 µL of a 40 mM biotin-PEG3-alkyne stock solution in DMSO was added (c_{final} = 1.0 mM) and each mixture was vortexed for 1 s. Next, 10 µL of Cu-THPTA mixture (5 mM $CuSO_4$ and 25 mM THPTA in H_2O) premixed at 37 °C for 10 min was added ($c_{final}(CuSO_4)$ = 0.1 mM; c_{final}(THPTA) = 0.5 mM). Then, 5.0 µL of an aminoguanidine hydrochloride stock solution (0.25 M in H_2O) was added (c_{final} = 2.5 mM), followed by addition of 5.0 µL of a sodium ascorbate stock solution (0.25 M in H_2O; c_{final} = 2.5 mM). The mixtures were vortexed for 2 s, transferred into a Thermocycler pre-heated at 37 °C, and incubated at 37 °C for

2 hours at 1000 rpm.

The samples were used for protein digest and biotin enrichment.

In vitro competition experiment

The reaction between glutathione (GSH) and an excess of **VTT** was performed in deuterated buffer (D-NaP$_i$, pH 7.4, 50 mM) and the reaction mixture was analyzed with ^{1}H NMR using 3-(trimethylsilyl)propionic-2,2,3,3-d$_4$ acid sodium salt (TSP) as an internal standard:

Stock solutions of GSH (50 mM), TSP (50 mM) were prepared in D_2O. D_2O (325 µL), D-NaP$_i$ (50 µL, pH 7.4, c = 0.5 M), stock solutions of TSP (50 µL) and GSH (50 µL) were pipetted into a 1.5 ml tube and mixed by pipetting the mixture up and down. Subsequently, a stock solution of **VTT** in DMF-d$_7$ (25 µL, c = 0.5 M) was added to the tube to obtain a mixture containing GSH (5.0 mM, 1.0 equiv.), TSP (5.0 mM, 1.0 equiv.), and **VTT** (25 mM, 5.0 equiv.) and the mixture was vortexed for 1 min. The ^{1}H NMR spectrum of the mixture was acquired 5 min after addition of **VTT**:

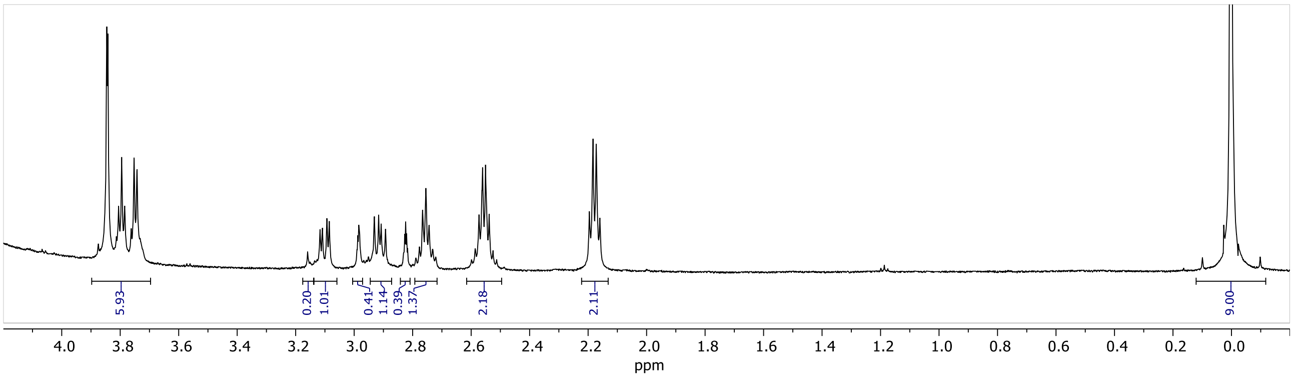

*Figure S1. ^{1}H NMR of reaction mixture containing 5 mM GSH, 25 mM **VTT**, 5 mM TSP as internal standard in D-NaP$_i$ (pH 7.4, 50 mM).*

The reaction between GSH and an excess of **NEM** was performed in deuterated buffer (D-NaP$_i$, pH 7.4, 50 mM) and the reaction mixture was analyzed with ^{1}H NMR using TSP as an internal standard:

Stock solutions of GSH (50 mM), TSP (50 mM) were prepared in D_2O. D_2O (325 µL), D-NaP$_i$ (50 µL, pH 7.4, c = 0.5 M), stock solutions of TSP (50 µL) and GSH (50 µL) were pipetted into a 1.5 mL tube and mixed by pipetting the mixture up and down. Subsequently, a stock solution of **NEM** in DMF-d$_7$ (25 µL, c = 0.5 M) was added to the tube to obtain a mixture containing GSH (5.0 mM, 1.0 equiv.), TSP (5.0 mM, 1.0 equiv.), and **NEM** (25 mM, 5.0 equiv.) and the mixture was vortexed for 1 min. The ^{1}H NMR spectrum of the mixture was acquired 5 min after addition of **NEM**:

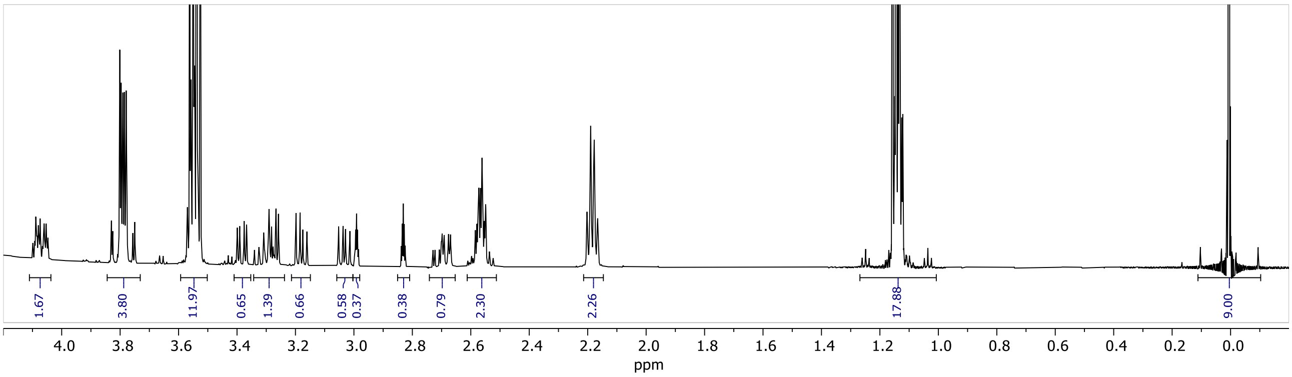

*Figure S2. ^{1}H NMR of reaction mixture containing 5 mM GSH, 25 mM **NEM**, 5 mM TSP as internal standard in D-*

NaP_i (pH 7.4, 50 mM).

The reaction between GSH and an excess of **VTT-NEM** was performed in deuterated buffer (D-NaP_i, pH 7.4, 50 mM) and the reaction mixture was analyzed with ^1H NMR using TSP as an internal standard:

Stock solutions of GSH (50 mM), TSP (50 mM) were prepared in D_2O. D_2O (325 µL), D-NaP_i (50 µL, pH 7.4, c = 0.5 M), stock solutions of TSP (50 µL) and GSH (50 µL) were pipetted into a 1.5 mL tube and mixed by pipetting the mixture up and down. Subsequently, 25 µL of a freshly prepared solution containing **NEM** (0.5 M) and **VTT** (0.5 M) in DMF-d_7 was added to the tube to obtain a mixture containing GSH (5.0 mM, 1.0 equiv.), TSP (5.0 mM, 1.0 equiv.), **VTT** (25 mM, 5.0 equiv.) and **NEM** (25 mM, 5.0 equiv.) and the mixture was vortexed for 1 min. The ^1H NMR spectrum of the mixture was acquired 5 min after addition of the mixture containing **VTT** and **NEM**:

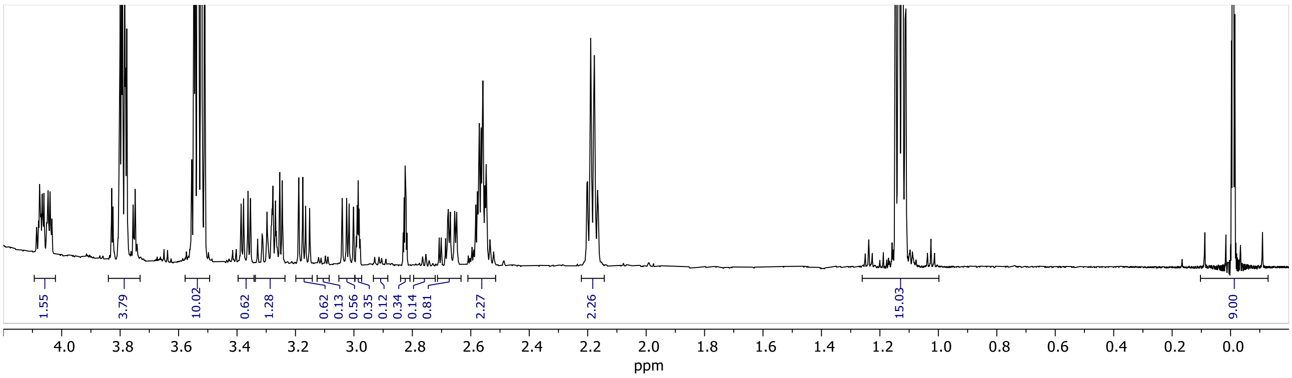

*Figure S3. ^1H NMR of reaction mixture containing 5 mM GSH, 25 mM **NEM**, 25 mM **VTT**, 5 mM TSP as internal standard in D-NaP_i (pH 7.4, 50 mM).*

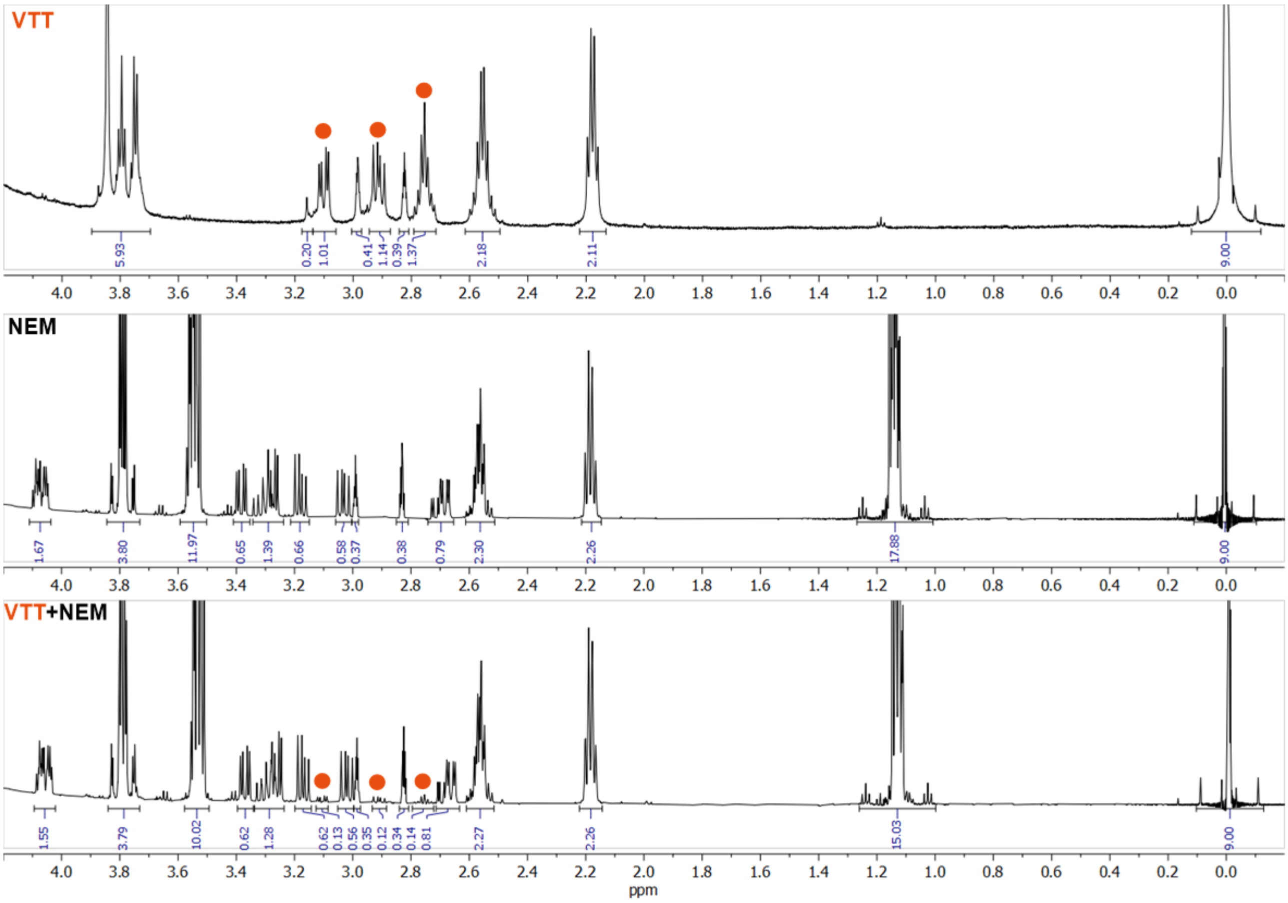

Figure S4. Combined ^1H NMR spectra from in vitro competition experiments.

The product peaks at 3.10, 2.90, and 2.75 ppm were integrated in the sample containing both **VTT** and **NEM** and referenced to the corresponding peaks in the sample with **VTT**:

$$\left(\frac{0.13}{1.01}+\frac{0.12}{1.14}+\frac{0.14}{1.37}\right)/\ 3\ =\ 0.11 \pm 0.01$$

Based on this value, the ratio between **NEM** and **VTT** products formed in the *in vitro* setup is 8:1.

Sample preparation for LC–MS/MS measurements

SP3 protocol

The LC–MS/MS sample preparation was performed via slightly modified SP3 workflow[6]. HEPES (pH 8.0, 0.5 M) was added to the lysates to obtain a HEPES concentration of 100 mM. Then, a 10% (w/w) stock solution of SDS in H_2O and a 1.0 M stock solution of TCEP in H_2O were added to the sample to obtain final concentrations of 1% (v/v) SDS and 10 mM TCEP. The mixtures were incubated for 30 min at 60 °C at 1000 rpm, allowed to cool down to 20–25 °C, and alkylated with chloroacetamide (c_{final} = 40 mM) for 1 hour at 37 °C at 1000 rpm.

For processing 20 µg of protein starting material, 4.0 µL of the magnetic beads stock (combined 1:1 Sera-Mag SpeedBeads (GE Healthcare, cat. no. 45152105050250) and Sera-Mag SpeedBeads (GE Healthcare, cat. no. 65152105050250)) were added to the reduced and alkylated lysates, the beads were resuspended by vortexing the mixtures for 1 s, followed by addition of abs. EtOH to obtain 50 % (v/v) EtOH mixture. The mixtures were incubated for 5 min at 24 °C at 1000 rpm, transferred to a magnetic rack, and the beads were washed three times with 400 µL of 80 % (v/v) EtOH in H_2O according to the protocol[6]. The on-bead digestion was performed in 100 µL of a freshly prepared 100 mM NH_4HCO_3 pH 8.0 buffer containing 0.4 µg of Trypsin/LysC mix (Promega, #V5072) at 37 °C at 1000 rpm for 18 hours. The samples were centrifuged for 3 min at 17,000 xg, the supernatants were transferred to new tubes, evaporated using SpeedVac, and submitted for LC–MS/MS measurements.

Protocol for biotin enrichment

1. Reduction and alkylation: 65 µL of HEPES (pH 8.0, 1.0 M) was added to the lysates after CuAAC reaction (each mixture contained 0.5 mg protein in 500 µL). Then, 33 µL of a 20% (w/w) stock solution of SDS in H_2O and a 13 µL of a 0.5 M stock solution of TCEP in H_2O were added to the samples. The mixtures were incubated for 30 min at 37 °C at 1000 rpm, 40 µL of a 0.66 M chloroacetamide stock solution in H_2O were added, and the mixtures were incubated for 1 hour at 37 °C at 1000 rpm.
2. SP3-digestion: 100 µL of the magnetic beads stock (combined 1:1 Sera-Mag SpeedBeads (GE Healthcare, cat. no. 45152105050250) and Sera-Mag SpeedBeads (GE Healthcare, cat. no. 65152105050250)) were added to the reduced and alkylated lysates, the beads were resuspended by vortexing the mixtures for 1 s, followed by addition of 750 µL of abs. EtOH. The mixtures were incubated for 10 min at 24 °C at 1000 rpm, transferred to a magnetic rack, and the beads were washed three times with 1000 µL of 80 % (v/v) EtOH in H_2O according to the SP3-protocol[8]. The on-bead digestion was performed in 400 µL of HEPES buffer (pH 8.0, 50 mM) containing 10 µg of Trypsin/LysC mix (Promega, #V5072) at 37 °C at 1000 rpm for 18 hours.
3. Heat treatment: The samples were centrifuged for 10 min at 21,000 xg, the supernatants were transferred to new tubes and incubated for 10 min at 95 °C at 1000 rpm to ensure the inactivation of trypsin prior to addition to NeutrAvidin beads.
4. Biotin-NeutrAvidin binding: 125 µL of NeutrAvidin beads slurry (ThermoFischer, #29200) were washed 3 times with 1 mL of PBS. Each washing step included addition of the buffer to the beads, vortexing the mixtures for 1 s, centrifugation at 1,700 xg for 2 min at 20–25 °C, and discarding the supernatant. Then, the heat-treated peptide mixtures were added to the tubes with washed NeutrAvidin beads at 20–25 °C, the mixtures were vortexed for 2 s and transferred to a

Thermocycler pre-cooled to 25 °C. The mixtures were incubated for 2 hours at 25 °C at 1000 rpm.

5. Washing step: The mixtures were centrifuged at 1,700 xg for 2 min at 20–25 °C, the supernatant was discarded. The beads were washed 5 times with 1 mL of PBS and 4 times with 1 mL of H_2O. Each washing step included addition of the buffer/water to the beads, vortexing the mixtures for 3 s, centrifugation at 1,700 xg for 2 min at 20–25 °C, and discarding the supernatant.
6. Peptide release: The washed beads were resuspended in 150 µL of the elution buffer (80% MeCN, 0.1% formic acid (v/v) in H_2O), vortexed for 3 s, and incubated for 10 min at 25 °C at 1000 rpm[7]. The mixtures were centrifuged at 1,700 xg for 2 min at 20–25 °C, the supernatant was transferred to new tubes. Then, the beads were resuspended in 150 µL of the elution buffer and incubated for 10 min at 72 °C at 1000 rpm. The mixtures were centrifuged at 1,700 xg for 2 min at 20–25 °C, the supernatant was combined with the mixtures from the first elution step.
7. Washing step: The beads were washed 2 times with 100 µL of the release buffer. Each washing step included addition of the release buffer (80% MeCN, 0.1% formic acid (v/v) in H_2O) to the beads, vortexing the mixtures for 3 s, centrifugation at 1,700 xg for 2 min at 20–25 °C, and combining the supernatant with the mixtures from step 6. The combined mixtures in the elution buffer were evaporated using SpeedVac.
8. Separation of the remaining beads: The pellets obtained in step 7 were washed 3 times with H_2O. First, the pellets were resuspended in 47 µL of H_2O, vortexed for 15 s, and centrifuged for 5 min at 21,000 xg at 20–25 °C. Then, 40 µL of the supernatant was carefully transferred to a new tube. The washing step was repeated two more times with addition and transferring 30 µL of H_2O each time. The combined mixtures without remaining beads were evaporated using SpeedVac and analyzed via LC–MS/MS using method B.

In cellulo protein modification in *E. coli* cells for SDS-PAGE analysis

20 mL of an *E. coli* cell culture in Miller-LB medium with an OD_{600} value of 0.7 were transferred into a new autoclaved Erlenmeyer flask. Next, 50 µL of a 4.0 M sodium azide stock solution in H_2O were added to the reaction mixtures according to the table below. The cultures were stirred in the presence of sodium azide for 10 min at 20–25 °C or 43 °C (see table below). Afterwards, 100 µL of DMF or a 1.0 M vinylthianthrenium stock solution in DMF was added to obtain different final concentrations of each reagent (see table below). In all cases, the final concentration of organic cosolvent was 0.5% (v/v). The cultures were shaken for 15 min at 20–25 °C or 43 °C (see table below).

Experiment #	Pre-incubation with 10 mM NaN_3	Reagent, final concentration	Incubation temperature after addition of the reagent
1	+	**VTT**, 0.5 mM	20–25 °C
2	+	**VTT**, 1.0 mM	20–25 °C
3	+	**VTT**, 5.0 mM	20–25 °C
4	+	**VTFT**, 1.0 mM	20–25 °C
5	**-**	**VTT**, 1.0 mM	20–25 °C
6	**-**	**VTFT**, 1.0 mM	20–25 °C
7	+	DMF	20–25 °C
8	+	**$^2H_3$-VTT**, 1.0 mM	43 °C
9	+	**$^{13}C_2$-VTT**, 1.0 mM	43 °C

Next, the cultures were transferred to an ice bath and centrifuged for 1 h with 4,200 xg at 4 °C. Then, the supernatant was discarded and the pellets were washed with 25 mL 25 mM HEPES (pH 8.0, 25 mM). The suspension was centrifuged for 10 min with 4,200 xg at 4 °C. Next, the supernatant was discarded and the cells were suspended in 5 mL lysis buffer (HEPES (pH 8, 50 mM), 50 mM NaCl, 5 mM EDTA, 5 mM DTT, 1% SDS, 1% Triton X-100, 1% NP-40, 1% Tween-20, 1% Deoxycholate, 1% Glycerol, 1 mM PMSF). The resuspended cells were lysed via sonication (5 × 2 min, 70 % power). Next, the lysed cells were centrifuged at 4 °C for 60 min at 20,800 xg. The supernatant was transferred into new reaction tubes and frozen in liquid nitrogen for later SDS-PAGE sample analysis.

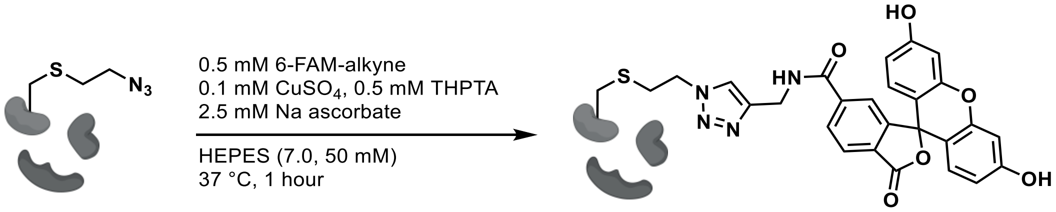

The lysates were thawed and the protein concentration was determined via BCA assay. The CuAAC reactions were performed as follows: At 20–25 °C, HEPES (pH 7.0; 50 mM) buffer was added to the lysates to obtain final volume of 94 µL. Then, 2.1 µL of a 24 mM 6-FAM alkyne stock solution in DMSO was added (c_{final} = 0.5 mM). Next, 2.0 µL of a Cu-THPTA mixture (5 mM $CuSO_4$ and 25 mM THPTA in

H_2O) premixed at 37 °C for 5 min was added ($c_{final}(CuSO_4)$ = 0.1 mM; c_{final}(THPTA) = 0.5 mM). Next, 2.5 µL of a sodium ascorbate stock solution (0.10 M in H_2O) was added (c_{final} = 2.5 mM). The mixtures were vortexed for 2 s, transferred into a Thermocycler pre-heated at 37 °C, and incubated at 37 °C for 60 min at 500 rpm.

Next, the samples were rebuffered four times in HEPES (pH 7.0, 50 mM) utilizing 3 kDa Amicon filter units. After rebuffering, the final volume was adjusted to 100 µL and the reaction mixtures were analyzed via SDS-PAGE analysis loading 2 µg protein per lane.

The pipetting scheme for SDS-PAGE analysis is as follows:

Lane	1	2	3	4	5	6	7	8	9	10
Assignment	Ladder	Exp.1	Exp.2	Exp.3	Exp.4	Exp.5	Exp.6	Exp.7	Exp.8	Exp.9

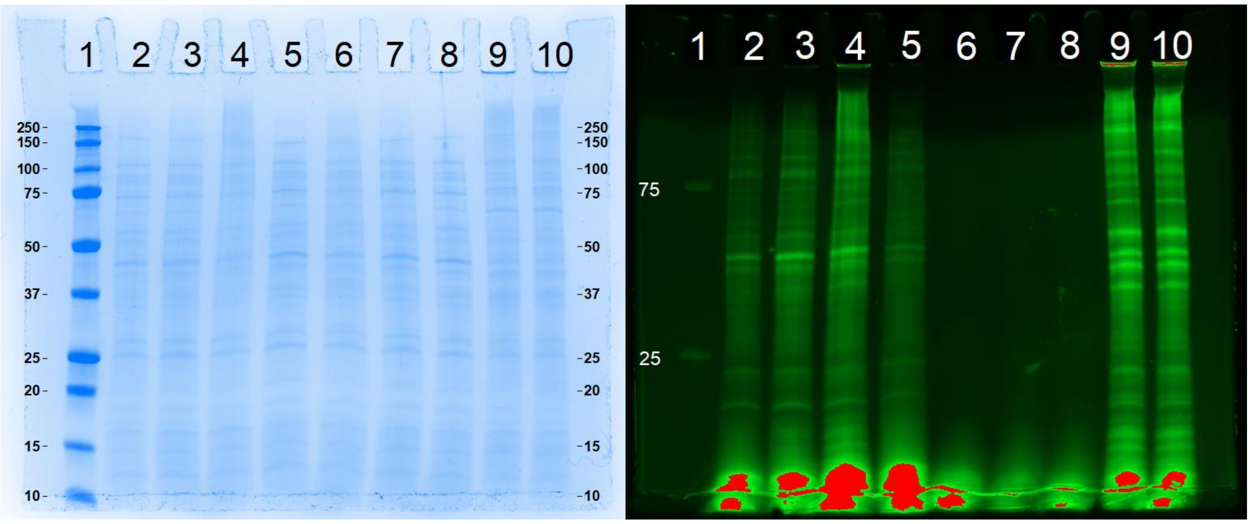

Figure S5. Protein gel after Coomassie blue staining (left) and the fluorescence image of the same gel (right).

The fluorescence intensity of each lane was quantified and normalized by the lane absorbance obtained for the Coomassie gels. Overexposed regions of the gel pictures were excluded from the analysis:

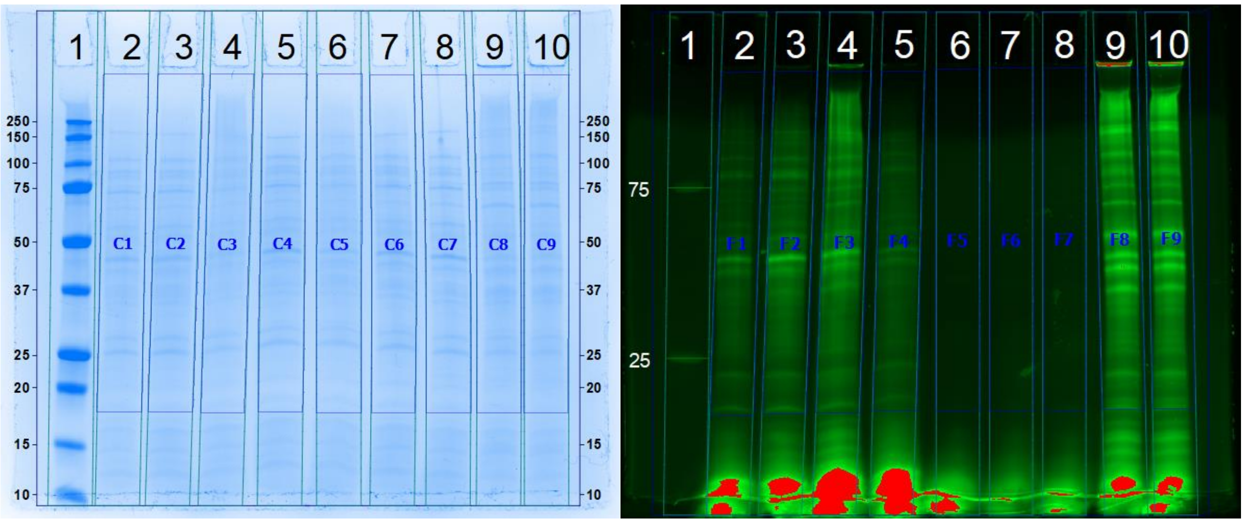

Figure S6. Protein gel after Coomassie staining (left) and the fluorescence image of the same gel (right). Volume quantification tool was used to determine the signal intensity of each lane.

Experiment #	Lane #	Coomassie signal intensity	Fluorescence signal intensity	Normalized fluorescence intensity
1	2	4.0×10^{9}	1.0×10^{9}	0.25
2	3	4.1×10^{9}	1.3×10^{9}	0.33
3	4	3.9×10^{9}	1.7×10^{9}	0.44
4	5	4.0×10^{9}	1.1×10^{9}	0.26
5	6	4.0×10^{9}	5.6×10^{8}	0.14
6	7	4.0×10^{9}	6.1×10^{8}	0.15
7	8	4.0×10^{9}	6.4×10^{8}	0.16
8	9	4.0×10^{9}	2.0×10^{9}	0.50
9	10	4.0×10^{9}	1.9×10^{9}	0.47

Apart from demonstrating incorporation of the azide in the modified proteins, the analysis of the normalized fluorescence intensities suggests that **VTFT** is less efficient than **VTT** in labeling proteins in *E. coli* cells. We therefore did not use **VTFT** for other experiments.

Membrane integrity studies

K562 cells

5 mL of K562 cells (1.2×10^6 cells / mL) in 1x Gibco RPMI 1640 Medium supplemented with 10% of heat-inactivated FCS was centrifuged for 5 min at 1,500 xg at 4 °C in a 15 mL Falcon tube, the supernatant was removed, the cells were resuspended in 5 mL of DPBS, centrifuged for 5 min at 1,500 xg at 4 °C, the supernatant was discarded, the cells were washed again with 5 mL of DPBS, and the supernatant was discarded. The cell pellet was resuspended in 30 mL of DPBS and transferred to a black / clear bottom 96-well plate (50 µL suspension / well). To each well, 50 µL of the CellTox® reagent mixture (freshly prepared by diluting the CellTox® (Promega, # G8741) stock solution 1:1000 (v/v) with DPBS) was added. Then, 1.0 µL of a DMSO stock solution containing various concentrations of **VTT** were added to the wells. In case of the control experiment with the lysis buffer, 4.0 µL of the Lysis Solution from the CellTox® kit was added to the wells. The last control experiment involved no addition of DMSO. Each experiment was performed in 4 replicates. The plate was incubated for 60 min at 37 °C in 5 % CO_2 in a humidified atmosphere and the fluorescence measurement was performed at λ_{ex} = 487 nm and λ_{em} = 535 nm.

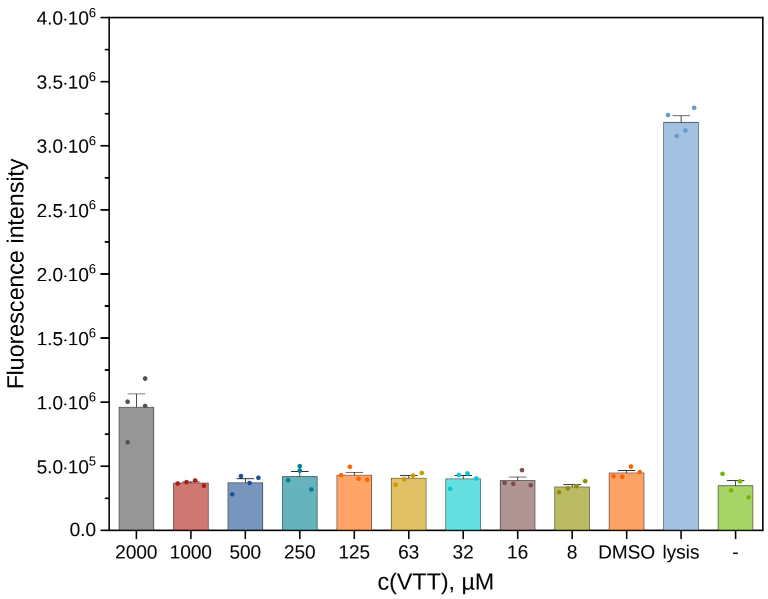

*Figure S7. Fluorescence intensities obtained after incubation of K562 cells supplemented with CellTox® reagent with solutions containing various concentrations of **VTT** in DMSO / 1% (v/v) DMSO / lysis buffer (lysis) / no DMSO (-). Individual data points are shown as dots, bars represent mean values, individual data points are shown as dots, and error bars indicate ± standard error.*

HEK cells

1.7 mL of confluent HEK cells (8.9×10^5 cells / mL) in 1x Gibco DMEM + GlutaMAX Medium supplemented with 10% of FCS was diluted and resuspended with 28.3 mL of the medium and transferred to a black / clear bottom 96-well plate (100 µL suspension / well). The well was incubated for 20 hours at 37 °C in 5 % CO_2 in a humidified atmosphere. The growth medium was removed and the cells were washed two times with 100 µL of DPBS. Then, 100 µL of the CellTox® reagent mixture (freshly prepared by diluting the CellTox® (Promega, # G8741) stock solution 1:500 (v/v) with DPBS) was added. Then, 1.0 µL of a DMSO stock solution containing various concentrations of **VTT** were added to the wells. In case of the control experiment with the lysis buffer, 4.0 µL of the Lysis Solution from the CellTox® kit was added to the wells. The last control experiment involved no addition of DMSO. Each experiment was performed in

4 replicates. The plate was incubated for 60 min at 37 °C in 5 % CO_2 in a humidified atmosphere and the fluorescence measurement was performed at λ_{ex} = 487 nm and λ_{em} = 535 nm.

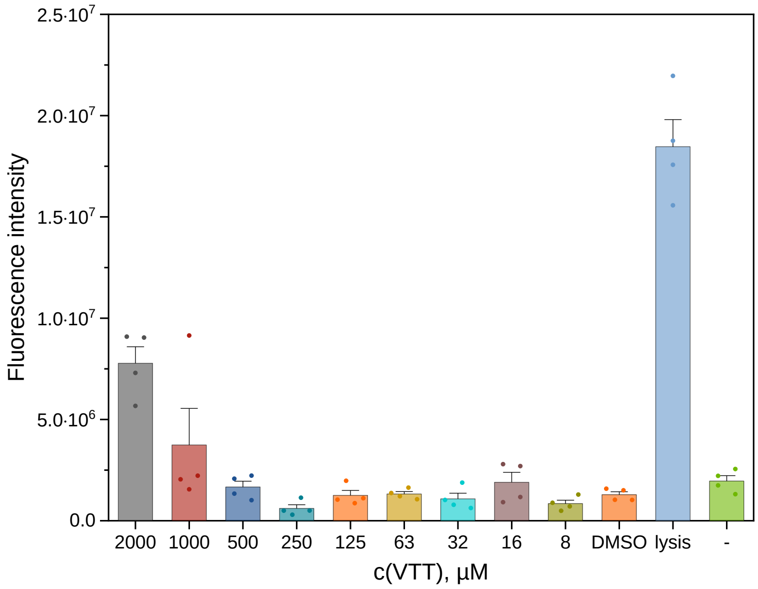

*Figure S8. Fluorescence intensities obtained after incubation of HEK cells supplemented with CellTox® reagent with solutions containing various concentrations of **VTT** in DMSO / 1% (v/v) DMSO / lysis buffer (lysis) / no DMSO (-). Individual data points are shown as dots, bars represent mean values, individual data points are shown as dots, and error bars indicate ± standard error.*

Influence of VTT concentration on protein recoveries

Since treatment with 5.0 mM **VTT** was required to achieve efficient labeling for *A. thaliana*, *S. cerevisiae*, and *N. tabacum* cells, we determined the influence of the elevated **VTT** concentration on the protein recoveries obtained after the cells were washed two times and lysed. Based on the isolated protein amounts, there is no significant loss of protein caused by the elevated **VTT** concentrations for *A. thaliana* and *S. cerevisiae*. In case of *N. tabacum*, we were able to recover at least 80 % of the protein after cell treatment with 5.0 mM **VTT**.

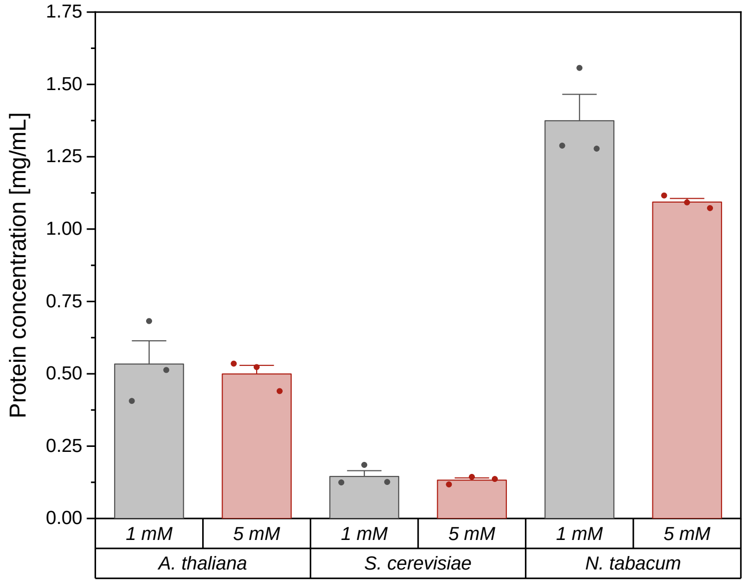

*Figure S9. Protein concentrations determined for the lysates after treatment of cells with **VTT** at different concentrations. Individual data points are shown as dots, bars represent mean values, and error bars indicate ± standard error.*

Viability studies

Assay with a delayed addition of resazurin reagent

45 mL of K562 cells (1.19×10^6 cells / mL) in 1x Gibco RPMI 1640 Medium supplemented with 10% of heat-inactivated FCS was split in two parts and centrifuged for 5 min at 1,500 xg at 4 °C in two 50 mL Falcon tubes. In each tube, the supernatants were removed, the cells were resuspended in 25 mL of DPBS, centrifuged for 5 min at 1,500 xg at 4 °C, the supernatant was discarded, the cells were washed again with 25 mL of DPBS, and the supernatant was discarded. The cell pellets were combined and resuspended in 10.8 mL of DPBS and transferred to a black / clear bottom 96-well plate (80 µL suspension / well) containing 0.8 µL of freshly prepared DMSO solutions of **VTT**, **NEM**, **IAA** alkyne, 0.8 µL of DMSO, or no DMSO, respectively, as control in the wells. Each experiment was performed in four replicates. The plate was incubated for 25 minutes at 37 °C in 5 % CO_2 in a humidified atmosphere, 20 µL of PrestoBlue™ HS reagent (Invitrogen, P50200) diluted 1:1 (v/v) with DPBS was added to each well, and the plate was incubated at 37 °C in 5 % CO_2 in a humidified atmosphere. The fluorescence measurements were performed various time points at λ_{ex} = 560 nm and λ_{em} = 610 nm.

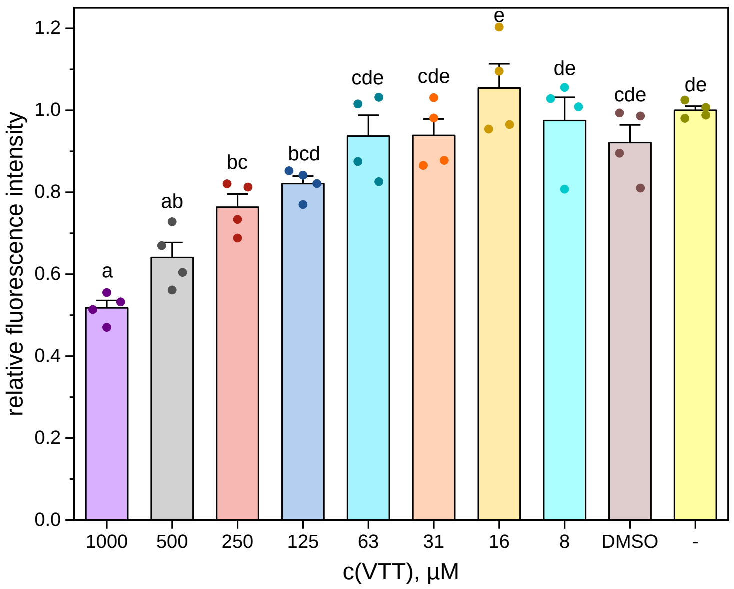

*Figure S10. Fluorescence intensities obtained after incubation of K562 cells with solutions containing various concentrations of **VTT** / 1% DMSO / no DMSO (-) and PrestoBlue™ reagent. PrestoBlue™ reagent was added 25 minutes after addition of the cells to the wells with alkylating reagents. The measurement was performed 15 minutes after addition of the PrestoBlue™ reagent to the wells. Individual data points are shown as dots, bars represent mean values, individual data points are shown as dots, and error bars indicate ± standard error. The significance of difference was determined using a one-way ANOVA, followed by post hoc Tukey test with $p < 0.05$.*

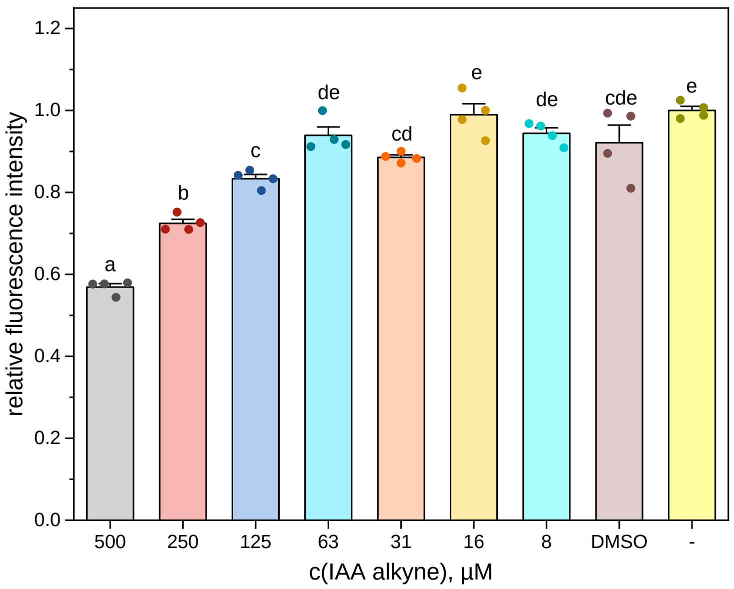

*Figure S11. Fluorescence intensities obtained after incubation of K562 cells with solutions containing various concentrations of **IAA** alkyne / 1% DMSO / no DMSO (-) and PrestoBlue™ reagent. PrestoBlue™ reagent was added 25 minutes after addition of the cells to the wells with alkylating reagents. The measurement was performed 15 minutes after addition of the PrestoBlue™ reagent to the wells. Individual data points are shown as dots, bars represent mean values, individual data points are shown as dots, and error bars indicate ± standard error. The significance of difference was determined using a one-way ANOVA, followed by post hoc Tukey test with p < 0.05.*

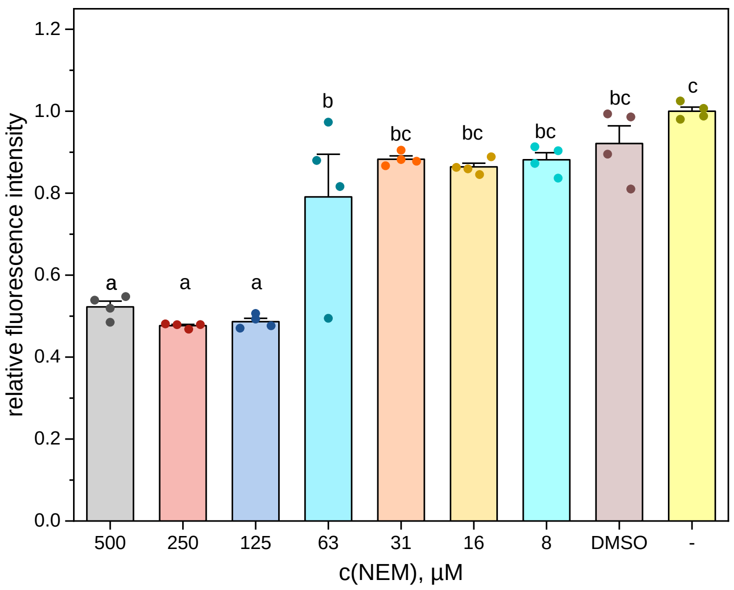

*Figure S12. Fluorescence intensities obtained after incubation of K562 cells with solutions containing various concentrations of **NEM** / 1% DMSO / no DMSO (-) and PrestoBlue™ reagent. PrestoBlue™ reagent was added 25 minutes after addition of the cells to the wells with alkylating reagents. The measurement was performed 15 minutes after addition of the PrestoBlue™ reagent to the wells. Individual data points are shown as dots, bars represent mean values, individual data points are shown as dots, and error bars indicate ± standard error. The significance of difference was determined using a one-way ANOVA, followed by post hoc Tukey test with p < 0.05.*

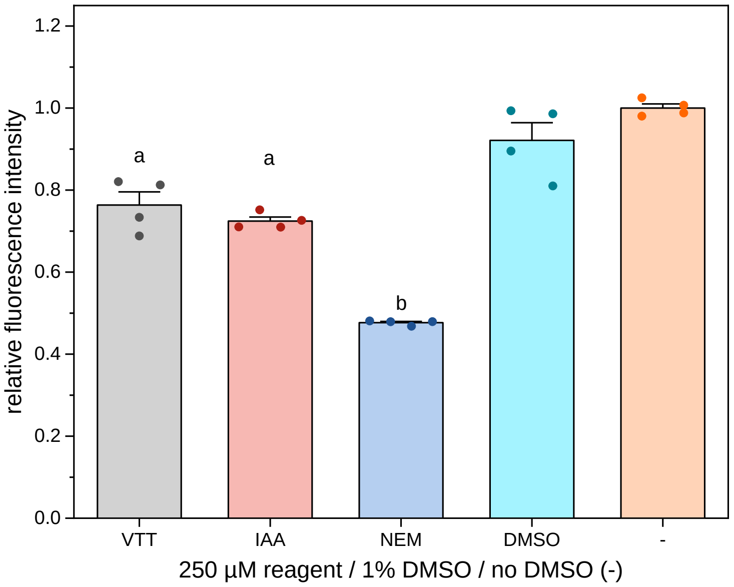

*Figure S13. Fluorescence intensities obtained after incubation of K562 cells with solutions containing 0.25 mM of **VTT** / **IAA** alkyne (**IAA**) / **NEM** / 1% DMSO / no DMSO (-) and PrestoBlue™ reagent. PrestoBlue™ reagent was added 25 minutes after addition of the cells to the wells with alkylating reagents. The measurement was performed 15 minutes after addition of the PrestoBlue™ reagent to the wells. Individual data points are shown as dots, bars represent mean values, individual data points are shown as dots, and error bars indicate ± standard error. The significance of difference was determined using a one-way ANOVA, followed by post hoc Tukey test with $p < 0.05$.*

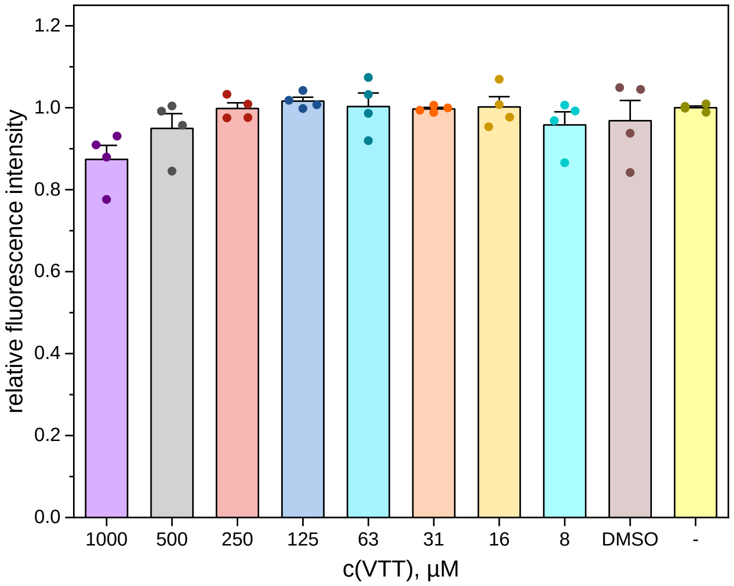

*Figure S14. Fluorescence intensities obtained after incubation of K562 cells with solutions containing various concentrations of **VTT** / 1% DMSO / no DMSO (-) and PrestoBlue™ reagent. PrestoBlue™ reagent was added 25 minutes after addition of the cells to the wells with alkylating reagents. The measurement was performed 4 hours after addition of the PrestoBlue™ reagent to the wells. Individual data points are shown as dots, bars represent mean values, individual data points are shown as dots, and error bars indicate ± standard error.*

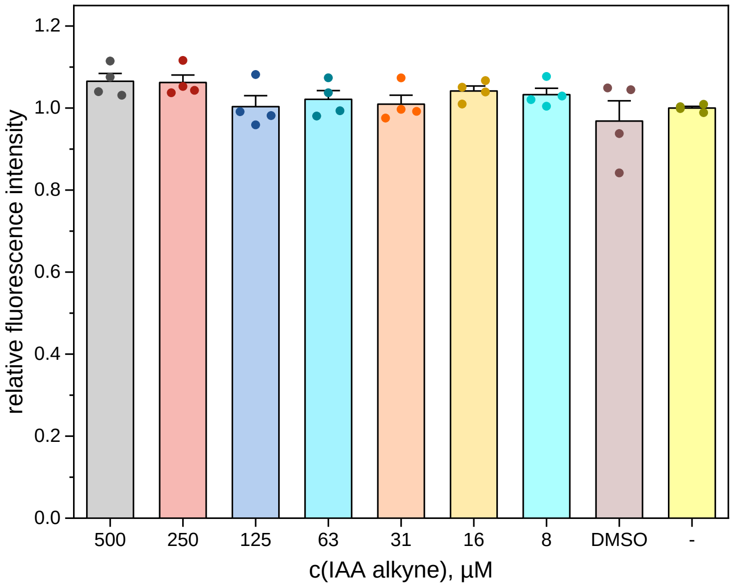

Figure S15. Fluorescence intensities obtained after incubation of K562 cells with solutions containing various concentrations of ***IAA*** *alkyne / 1% DMSO / no DMSO (-) and PrestoBlue™ reagent. PrestoBlue™ reagent was added 25 minutes after addition of the cells to the wells with alkylating reagents. The measurement was performed 4 hours after addition of the PrestoBlue™ reagent to the wells. Individual data points are shown as dots, bars represent mean values, individual data points are shown as dots, and error bars indicate ± standard error.*

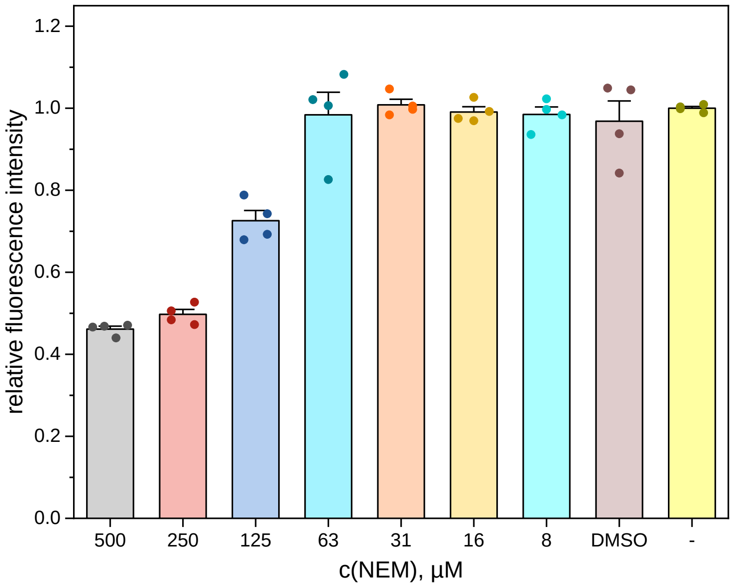

Figure S16. Fluorescence intensities obtained after incubation of K562 cells with solutions containing various concentrations of ***NEM*** */ 1% DMSO / no DMSO (-) and PrestoBlue™ reagent. PrestoBlue™ reagent was added 25 minutes after addition of the cells to the wells with alkylating reagents. The measurement was performed 4 hours after addition of the PrestoBlue™ reagent to the wells. Individual data points are shown as dots, bars represent mean values, individual data points are shown as dots, and error bars indicate ± standard error.*

Assay with direct addition of resazurin reagent

48 mL of K562 cells (1.12×10^6 cells / mL) in 1x Gibco RPMI 1640 Medium supplemented with 10% of heat-inactivated FCS was split in two parts and centrifuged for 5 min at 1,500 xg at 4 °C in two 50 mL Falcon tubes. In each tube, the supernatants were removed, the cells were resuspended in 25 mL of DPBS, centrifuged for 5 min at 1,500 xg at 4 °C, the supernatant was discarded, the cells were washed again with 25 mL of DPBS, and the supernatant was discarded. The cell pellets were combined and resuspended in 10.8 mL of DPBS and transferred to a black / clear bottom 96-well plate (80 µL suspension / well) containing 1.0 µL of freshly prepared DMSO solutions of **VTT**, **NEM**, **IAA** alkyne, 1.0 µL of DMSO, or no DMSO, respectively, as control in the wells. 20 µL of PrestoBlue™ HS reagent (Invitrogen, P50200) diluted 1:1 (v/v) with DPBS was added immediately after addition of the cell suspension to the wells. Each experiment was performed in four replicates. The plate was incubated at 37 °C in 5 % CO_2 in a humidified atmosphere and the fluorescence measurements were performed at various time points λ_{ex} = 560 nm and λ_{em} = 610 nm.

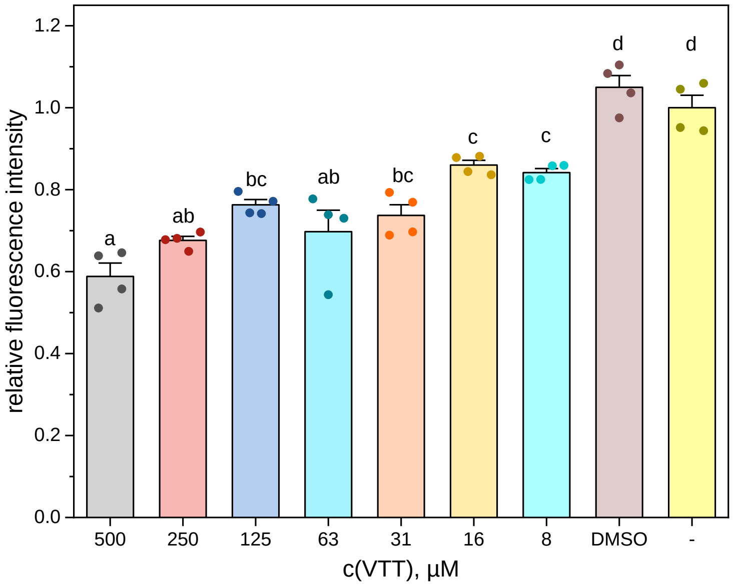

*Figure S17. Normalized fluorescence intensities obtained after incubation of K562 cells with solutions containing various concentrations of **VTT** / 1% DMSO / no DMSO (-) and PrestoBlue™ reagent. The measurement was performed 15 minutes after addition of the PrestoBlue™ reagent to the wells. Individual data points are shown as dots, bars represent mean values, individual data points are shown as dots, and error bars indicate ± standard error. The significance of difference was determined using a one-way ANOVA, followed by post hoc Tukey test with $p <$ 0.05.*

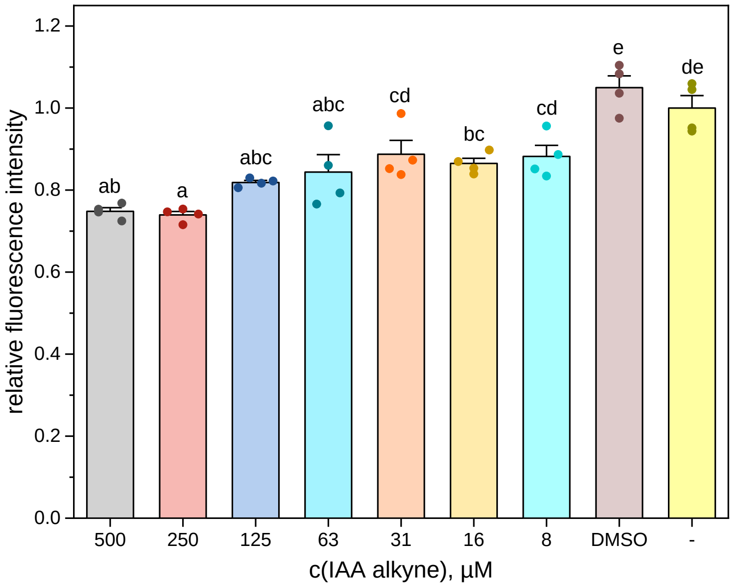

*Figure S18. Normalized fluorescence intensities obtained after incubation of K562 cells with solutions containing various concentrations of **IAA** alkyne / 1% DMSO / no DMSO (-) and PrestoBlue™ reagent. The measurement was performed 15 minutes after addition of the PrestoBlue™ reagent to the wells. Individual data points are shown as dots, bars represent mean values, individual data points are shown as dots, and error bars indicate ± standard error. The significance of difference was determined using a one-way ANOVA, followed by post hoc Tukey test with $p < 0.05$.*

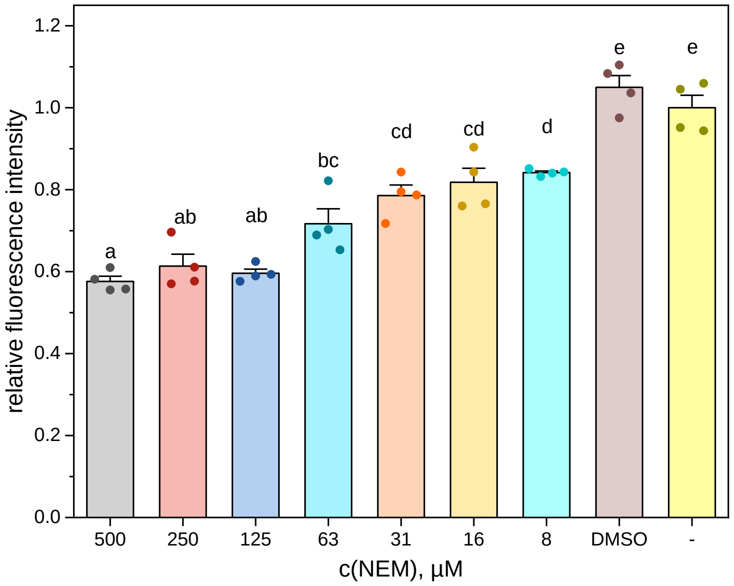

*Figure S19. Normalized fluorescence intensities obtained after incubation of K562 cells with solutions containing various concentrations of **NEM** / 1% DMSO / no DMSO (-) and PrestoBlue™ reagent. The measurement was performed 15 minutes after addition of the PrestoBlue™ reagent to the wells. Individual data points are shown as dots, bars represent mean values, individual data points are shown as dots, and error bars indicate ± standard error. The significance of difference was determined using a one-way ANOVA, followed by post hoc Tukey test with $p < 0.05$.*

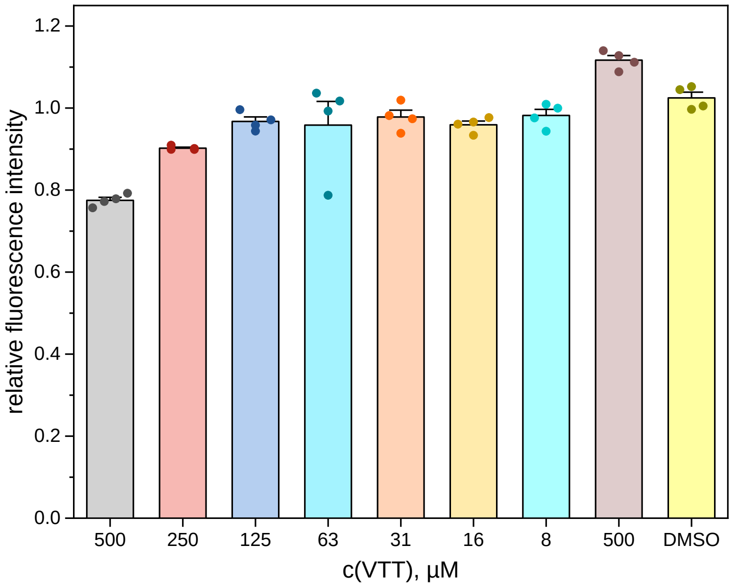

*Figure S20. Normalized fluorescence intensities obtained after incubation of K562 cells with solutions containing various concentrations of **VTT** / 1% DMSO / no DMSO (-) and PrestoBlue™ reagent. The measurement was performed 4 hours after addition of the PrestoBlue™ reagent to the wells. Individual data points are shown as dots, bars represent mean values, individual data points are shown as dots, and error bars indicate ± standard error.*

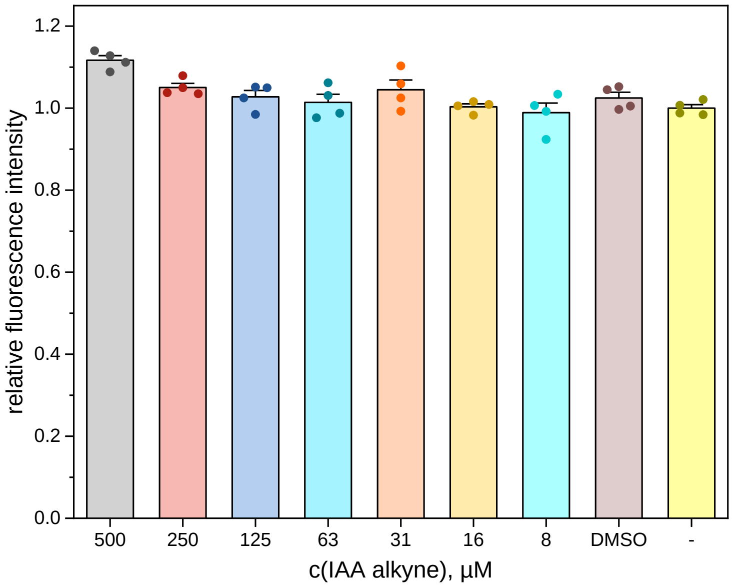

*Figure S21. Normalized fluorescence intensities obtained after incubation of K562 cells with solutions containing various concentrations of **IAA** alkyne / 1% DMSO / no DMSO (-) and PrestoBlue™ reagent. The measurement was performed 4 hours after addition of the PrestoBlue™ reagent to the wells. Individual data points are shown as dots, bars represent mean values, individual data points are shown as dots, and error bars indicate ± standard error.*

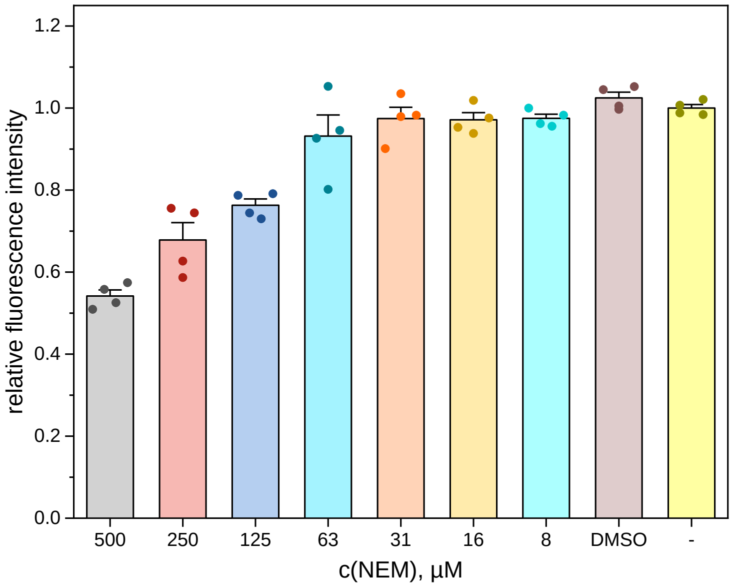

Figure S22. Normalized fluorescence intensities obtained after incubation of K562 cells with solutions containing various concentrations of ***NEM*** */ 1% DMSO / no DMSO (-) and PrestoBlue™ reagent. The measurement was performed 4 hours after addition of the PrestoBlue™ reagent to the wells. Individual data points are shown as dots, bars represent mean values, individual data points are shown as dots, and error bars indicate ± standard error.*

Resazurin was added either immediately or 25 minutes after addition of the alkylating reagents to assess the acute effects of the alkylating reagents on cellular redox homeostasis within the depicted timeframe used for cell labelling experiments with **VTT**. The observed variations in fluorescence intensities between measurements in the two experimental setups – despite equal incubation times – indicate that the obtained values represent cellular stress response rather than actual cell viability. To determine cellular viability, we recommend adding resazurin at later time points and perform measurements until the observed viability values remain constant.

In cellulo crosslinking of GrxC5

Plasmid generation and amplification

The coding sequence of *A. thaliana* GRXC5 (AT4G28730), excluding the chloroplast transit peptide, was amplified from leaf cDNA using gene-specific primers. The resulting PCR product was cloned into the pEXP-NT-TOPO vector (Invitrogen) via thymidine-adenosine (TA) cloning and verified by sequencing[8].

Forward: 5'-ACTTCTTCTTCTTCGGCTGCATC-3'

Reverse: 5'-AACTCTGACCGTTTTTACCGTTG-3'

The plasmid was transformed into One Shot™ TOP10 Chemically Competent *E. coli* (Invitrogen) by a heat shock protocol and grown over night on agar plates supplemented with kanamycin (50 µg/ml). A single colony was transferred to 20 mL of Miller-LB medium (50 µg/ml kanamycin) and incubated at 37 °C overnight. Plasmid purification was performed by NucleoSpin Plasmid kit (Macherey-Nagel) following the manufacturer's instructions.

In cellulo crosslinking with VTT and protein purification

The experiment was performed as 3 biological replicates.

E. coli NiCo21 (DE3) cells were transformed with the plasmid DNA (GrxC5 in pEXP5-NT/TOPO). A 50 mL overnight culture in Miller-LB medium supplemented with 100 µg/mL ampicillin was incubated at 37 °C with shaking (120 rpm). On the next day, 100 mL of fresh LB medium containing 100 µg/mL ampicillin was inoculated with 5 mL of the overnight culture and incubated at 37 °C until the culture reached an OD_{600} of 0.6. Protein expression was induced by the addition of 40 µL of 1.0 M IPTG stock solution in H_2O, followed by an incubation at 19 °C overnight under continuous shaking.
On the following day, the OD_{600} was determined (2.54), and 1 mL of a 0.10 M **VTT** stock solution in DMSO was added to the culture, followed by an incubation for 15 min at 37 °C with gentle shaking. Cells were harvested by centrifugation at 4,600 xg for 30 min at 4 °C. The supernatant was discarded, and the pellet was washed once with HEPES buffer (pH 7.0, 50 mM), followed by a second centrifugation step (10 min; 4,600 xg, 4 °C). The supernatant was discarded and the pellet was frozen in liquid nitrogen.
For lysis, the pellet was resuspended in 9.3 mL of NEBExpress Cell Lysis Reagent (NEB) and incubated for 30 min at room temperature. The suspension was then supplemented with 150 µL lysozyme solution (10 mg/mL) and further incubated for 30 min at 20-25 °C under gentle shaking. Subsequently, the mixture was subjected to sonication (1 × 3 min, 70 % amplitude) and centrifuged at 20,830 xg for 90 min at 4 °C. The clear supernatant was incubated with 1.5 mL Ni-NTA agarose beads (ROTI®Garose-His/Ni-NTA, Carl Roth), which were pre-equilibrated with lysis buffer (Tris-HCl (pH 8.0, 50 mM), 0.30 M NaCl, 5 mM imidazole). The mixture was incubated for 1 hour at 4 °C under gentle rotation.
Afterwards, the lysate-bead mixture was loaded onto a gravity-flow column. The beads were washed with four column volumes of wash buffer I (Tris-HCl (pH 8.0, 50 mM), 0.30 M NaCl and 10 mM imidazole), followed by one column volume of wash buffer II (Tris-HCl (pH 8.0, 50 mM), 0.30 M NaCl and 20 mM imidazole) and two additional column volumes of wash buffer I. Bound proteins were eluted by incubating the beads twice with 1.5 mL of elution buffer (Tris-HCl (pH 8.0, 50 mM), 0.30 M NaCl, 0.25 M imidazole) for 10 min each prior to elution. The eluates were combined (3 mL total) and dialyzed against 2 L of HEPES buffer (pH 8.0, 50 mM) at 4 °C. After 3 hours, the buffer was replaced with 2 L of HEPES buffer (pH 8.0, 50 mM), and dialysis was continued overnight. On the following day, the dialysis was stopped,

and the purified protein was aliquoted into reaction tubes and stored at –78 °C for subsequent LC-MS/MS sample preparation.

In-gel protein digestion

The protein concentration of each sample was determined via BCA assay, 15 µg protein was applied for SDS-PAGE separation. The gel was stained with Coomassie for 13 hours and washed with water for 3 hours. The bands corresponding to the overexpressed protein were cut into small (~1 mm^3) cubes and transferred to 1.5 mL tubes. The gel pieces were washed twice with 0.5 mL of H_2O, destained 2 times by addition of 100 µL 50% MeCN in 50 mM NH_4HCO_3, incubation for 15 min at 25 °C. The washing solutions were discarded. Then, 0.5 mL of MeCN was added and the samples were incubated for 10 min at 25 °C.

The solvent was discarded, 100 µL of a freshly prepared 10 mM TCEP solution in 100 mM NH_4HCO_3 was added to each sample, the samples were transferred to a thermocycler preheated to 56 °C, and the samples were incubated for 30 min at 1000 rpm. Then, the solution was discarded and the gel pieces were dehydrated with 0.5 mL MeCN for 10 min at 25 °C. After removal of the liquid, 100 µL of a freshly prepared 55 mM chloroacetamide solution in 100 mM NH_4HCO_3 was added to each sample, and the samples were incubated at 25 °C for 60 min at 1000 rpm. Then, the solution was discarded and the gel pieces were dehydrated with 0.5 mL MeCN for 10 min at 25 °C. After removal of the liquid, 100 µL of a freshly prepared trypsin (Promega; #V5111) solution (2.5 ng/µL) in 100 mM NH_4HCO_3 was added to each sample and the samples were incubated for 90 min at 4 °C. Then, the samples were transferred to an incubator preheated at 37 °C and incubated for 19 hours.

0.2 mL of a 1:2 (v/v) 5% formic acid in H_2O : MeCN solution was added to each sample and the samples were incubated for 15 min at 37 °C at 500 rpm. The solutions were transferred to new tubes, the samples were evaporated using SpeedVac and analyzed via LC–MS/MS using method C.

DATA ANALYSIS

Quantification of labeling efficiency

Modification search

Processing of raw data was performed using MaxQuant 2.6.3.0[9] *on the HPC system Raven at the Max Planck Computing and Data Facility*. MS^2 spectra were assigned to the reference proteomes from Uniprot (*H. sapiens*: UP000005640; *A. thaliana*: UP000006548; *B. subtilis*: UP000001570; *E. coli*: UP000000625; *B. vulgaris*: UP000035740; *S. cerevisiae*: UP000002311; *N. tabacum*: UP000084051). Trypsin specificity was required and a maximum of two missed cleavages was allowed. Carbamidomethylation of cysteine, oxidation of methionine, and protein N-terminal acetylation were set as variable modifications. The **VTT**-based modifications were defined as a variable modifications on Cys with the following formulas: C_2H_4O for the episulfonium hydrolysis product formed with **VTT**; $^{13}C_2H_4O$ for the episulfonium hydrolysis product formed with **$^{13}C_2$-VTT**; $C_2H_3N_3$ for the azidated product; $C_{21}H_{34}N_6O_5S$ for the biotin-enriched product formed with **VTT** after CuAAC reaction; $C_{21}H_{31}D_3N_6O_5S$ for the biotin-enriched product formed with **$^2H_3$-VTT** after CuAAC reaction:

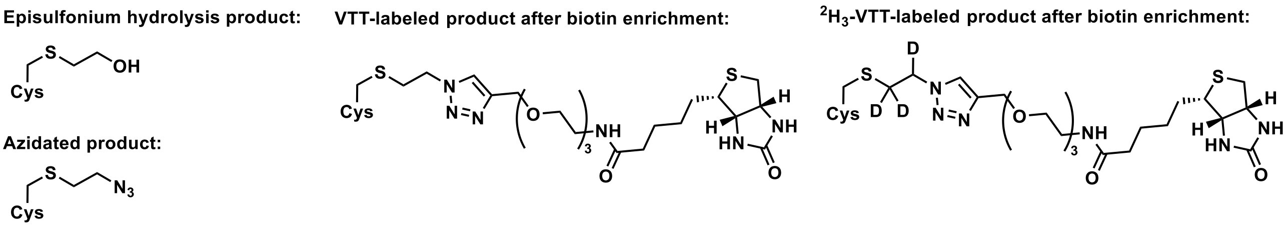

A false discovery rate of 1% for peptide spectrum matches and proteins was applied.

The total number of peptides of interest was extracted after filtering off the decoy peptides and potential contaminants from the output table.

Labeling efficiencies

The labeling efficiencies were calculated by normalizing the number of the identified Cys-modified peptides by the number of Cys-containing peptides detected in each run.

Cell type	c(VTT), mM	# Replicate	# Cys peptides	# Cys-C_2H_4OH peptides	Labeling efficiency
H. sapiens, K562	0.25	1	1198	314	26%
		2	1201	298	25%
		3	1154	355	31%
H. sapiens, HEK	0.25	1	1244	319	26%
		2	1015	245	24%
		3	1231	276	22%
A. thaliana	1.0	1	661	44	7%
		2	426	20	5%
		3	751	44	6%
A. thaliana	5.0	1	612	205	33%
		2	1150	188	16%
		3	1223	222	18%
B. subtilis	1.0	1	579	150	25.9%

		2	668	174	26.0%
		3	642	165	25.7%
B. vulgaris	1.0	1	260	79	30%
		2	195	58	30%
		3	282	69	24%
S. cerevisiae	1.0	1	546	50	9%
		2	427	31	7%
		3	278	16	6%
S. cerevisiae	5.0	1	325	64	19.7%
		2	315	57	18.1%
		3	217	40	18.4%
N. tabacum	1.0	1	584	69	12%
		2	842	50	6%
		3	804	49	6%
N. tabacum	5.0	1	730	154	21%
		2	631	137	22%
		3	742	121	16%
E. coli	1.0	1	722	177	25%
		2	673	190	28%
		3	756	175	23%

Gene ontology analysis

GO annotations were obtained for the proteins that had modified peptides detected in at least two biological replicates. The annotations exported from QuickGO[10] after restricting the search to Swiss-Prot entries in the canonical reference proteome. Only manually curated annotations backed by primary literature were considered for the analysis: direct or high-throughput experimental evidence (ECO:0000269 EXP, 0314 IDA, 0315 IMP, 0316 IGI, 0270 IEP, 0353 IPI, as well as the high-throughput counterparts 0007005 / 7001 / 7003 / 7007 / 6056), traceable author statements (ECO:0000304 TAS) and curator inferences (ECO:0000305 IC). Annotations based on sequence transfer or fully automatic pipelines (ECO:0000250, 0247, 0266, 0255, 0501) and entries lacking supporting data (ECO:0000303 NAS, 0000307 ND) were excluded, as well as all negation (`NOT|`…) qualifiers. The filtered GO terms were then mapped to the goslim_generic subset and QuickGO's "Use these terms as an exact match" option was used.

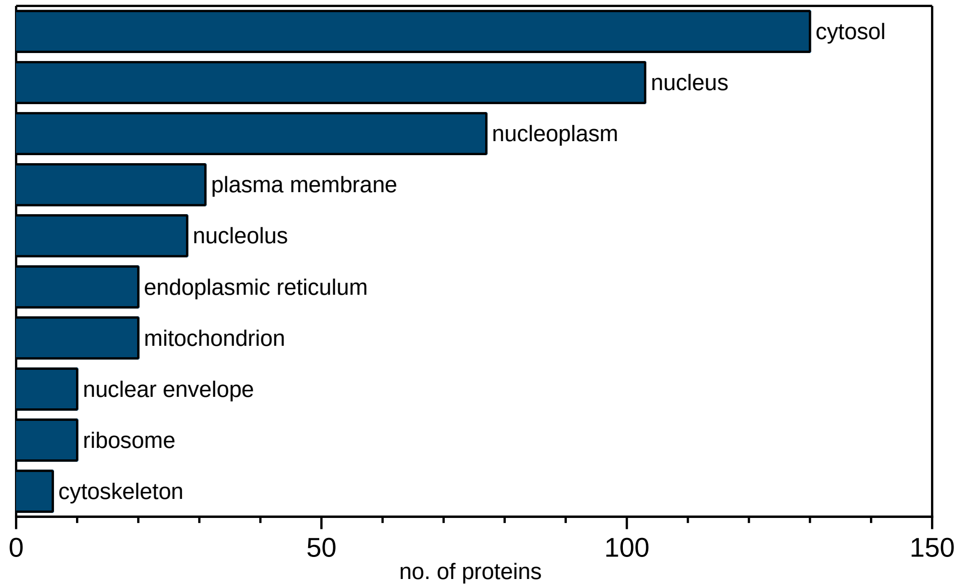

Figure S23. Gene ontology annotation (top 10) of intracellular proteins that were labeled in K562 cells.

Quantitative LC–MS/MS analysis

Processing of raw data was performed using MaxQuant 2.6.3.0[9]. MS^2 spectra were assigned to the reference proteomes from Uniprot (*H. sapiens*: UP000005640; *E. coli*: UP000000625). Trypsin specificity was required and a maximum of two missed cleavages was allowed. Oxidation of methionine and protein N-terminal acetylation were set as variable modifications. Modifications introduced by the light and heavy reagents were defined as the corresponding labels on Cys with the following formulas: $C_{21}H_{34}N_6O_5S$ as the light label and $C_{21}H_{31}D_3N_6O_5S$ as the heavy label for the samples with biotin-enriched samples from experiments with K562 cells; C_2H_4O as the light label and $^{13}C_2H_4O$ as the heavy label for the full lysate samples from experiments with *E. coli* cells. A false discovery rate of 1% for peptide spectrum matches and proteins was applied.

After filtering off the decoy peptides and potential contaminants from the output table, the lists with H/L ratios for the quantified peptide pairs were extracted for the analysis.

Competition experiments with *E. coli* cells

Competition between VTT and IAA alkyne

The label swap strategy was utilized to increase the reliability of the obtained results. Heavy-labeled **$^{13}C_2$-VTT** was used as the reference for the quantification of the peptides modified with **VTT** in the presence of **IAA** alkyne, whereas **VTT** was used as the reference for the quantification of the peptides modified with **$^{13}C_2$-VTT** in the presence of **IAA** alkyne. The initial modification search has revealed that similar number of **VTT**- and **$^{13}C_2$-VTT**-modified peptides was identified in both cases, suggesting no influence of equimolar **IAA** alkyne on the labeling efficiency of **VTT**:

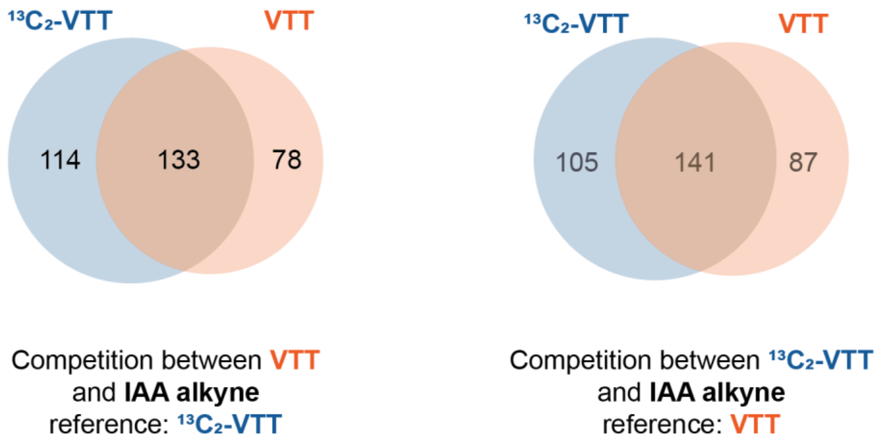

*Figure S24. Venn diagrams showing the number of **VTT**-modified peptides detected across three biological replicates for the competition experiments between **VTT** and **IAA** alkyne and between **$^{13}C_2$-VTT** and **IAA** alkyne.*

Furthermore, twice as many **VTT**-modified peptides relative to **IAA** alkyne were detected in both cases:

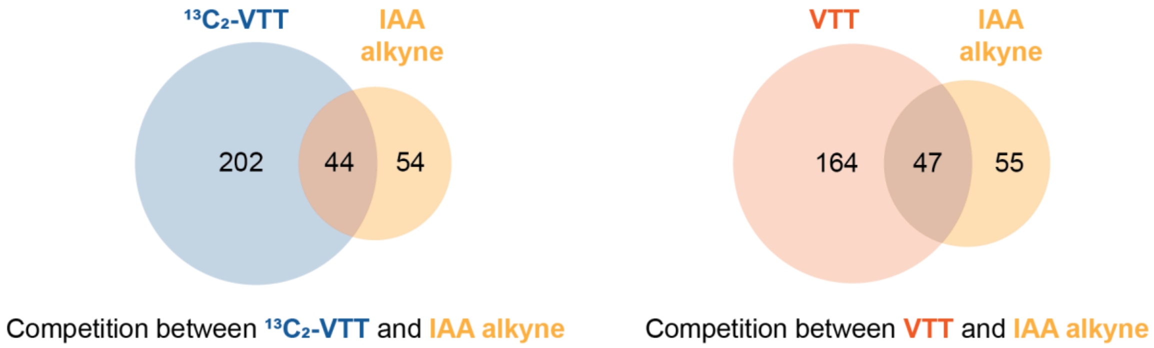

Figure S25. Venn diagrams showing the number of ***$^{13}C_2$-VTT****-,* ***VTT****- and* ***IAA*** *alkyne-modified peptides detected across three biological replicates for the competition experiments between* ***$^{13}C_2$-VTT*** *and* ***IAA*** *alkyne and between* ***VTT*** *and* ***IAA*** *alkyne.*

For quantification of the intensity ratios, a database search using the quantification feature of MaxQuant[7] was performed. The mean of the median H/L ratios obtained for three biological replicates is 1.11±0.04 for the experiment with **$^{13}C_2$-VTT** as the reference and 1.1±0.05 for the experiment with **VTT** as the reference:

Reference	Reagent mix	Replicate	# quantified peptide pairs	Median of H/L ratios
**$^{13}C_2$-VTT**	**VTT + IAA** alkyne	1	21	1.2
		2	23	1.1
		3	38	1.1
**VTT**	**$^{13}C_2$-VTT + IAA** alkyne	1	25	1.1
		2	30	1.1
		3	24	1.0

Competition between VTT and NEM

For quantification of the intensity ratios, a database search using the quantification feature of MaxQuant[9] was performed. The mean of the median H/L ratios obtained for three biological replicates is 1.9±0.3:

Reference	Reagent mix	Replicate	# quantified peptide pairs	Median of H/L ratios
**$^{13}C_2$-VTT**	**VTT + NEM**	1	13	2.4
		2	17	1.4
		3	13	2.1

Competition experiments with K562 cells

For quantification of the intensity ratios, a database search using the quantification feature of MaxQuant[9] was performed. The obtained ratios of MS^1 intensities for each of the quantified **$^2H_3$-VTT** / **VTT** peptide pair were plotted to visualize the data distribution for each biological replicate:

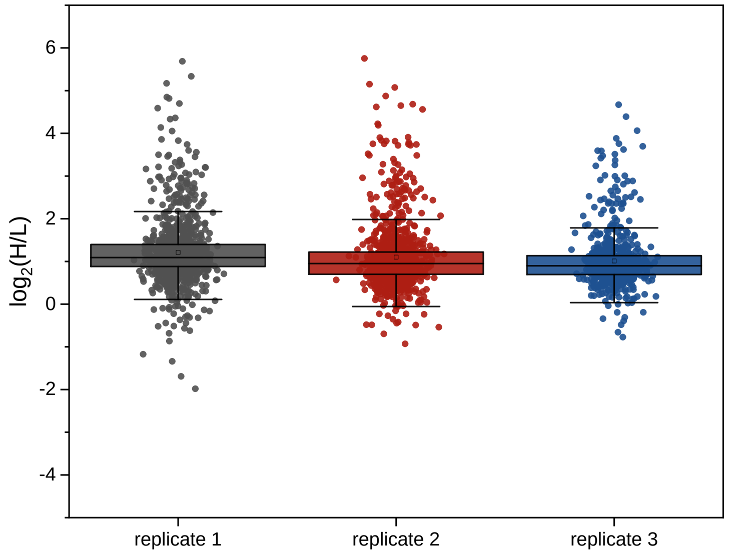

*Figure S26. Box plots showing the log_2-ratios of MS^1 intensities between heavy-labeled **$^2H_3$-VTT** (H) and unlabeled **VTT** (L) across biological replicates. The box spans from the first quartile (the 25th percentile) to the third quartile (the 75th percentile), to capture the interquartile range, which contains the middle 50% of the data. Horizontal line indicates median, whiskers extend to 1.5 × interquartile range from box boundaries.*

The mean of the median H/L ratios obtained for three biological replicates is 2.0±0.1:

Replicate	# quantified peptide pairs	Median of H/L ratios
1	1078	2.13
2	1007	1.93
3	1057	1.88

Intersection analysis of identified peptides

To compare peptide identities between several sample groups, the lists of detected peptides with zero missed cleavages that were not decoys or potential contaminants were extracted from the output table after database search with MaxQuant and matches between unique peptides from both groups were identified. The overlaps between peptides detected in all biological replicates were visualized using Venn diagram. A detailed analysis for overlaps between all identified peptides in each biological replicate were obtained using UpSet plots[11].

Labeling of K562 cells and K562 lysates

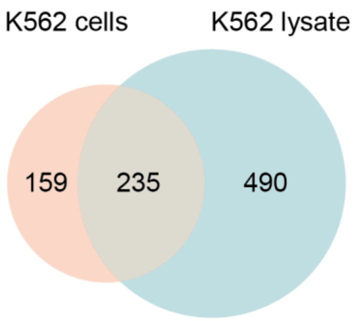

*Figure S27. Venn diagram with the numbers of unique modified peptides identified in all biological replicates after protein modification in K562 cells or lysates with **VTT**.*

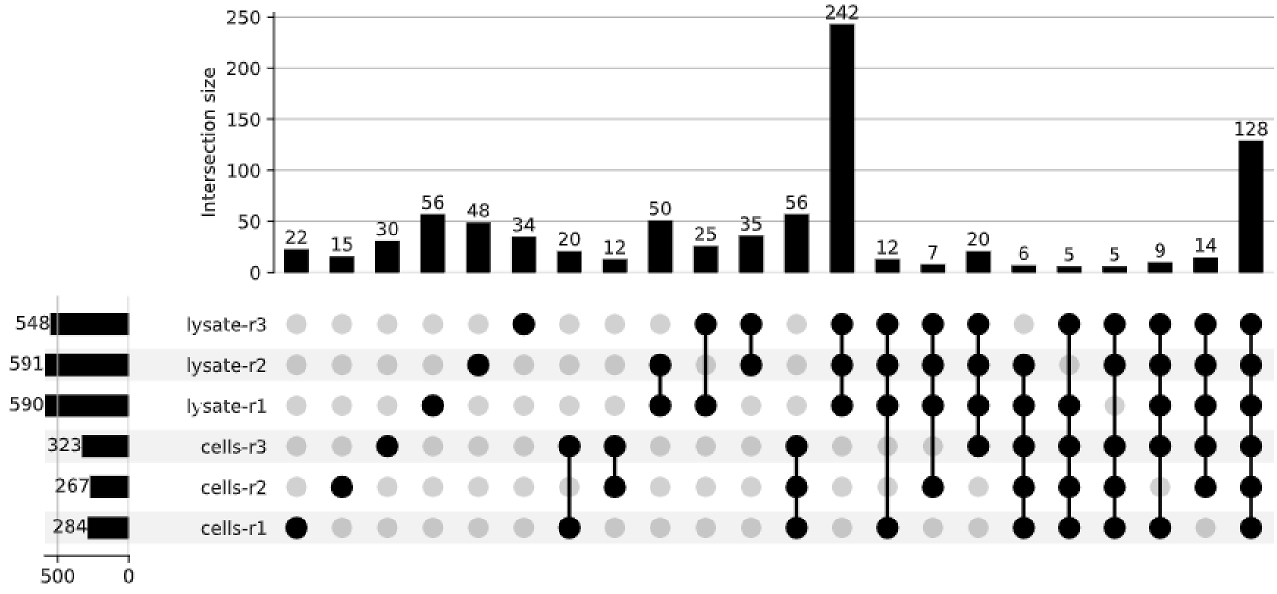

Figure S28. Intersection analysis of modified peptides identified in the biological replicates (r1, r2, r3) after protein modification in cellulo (cells) or in vitro (lysate). Each vertical bar represents a unique combination of input sets (indicated by filled dots in the matrix below the x-axis); the bar height equals the number of elements shared exclusively by that combination. Horizontal bars to the left show the total size of each individual set. Intersections containing fewer than five elements are omitted for clarity.

Labeling of *E. coli* cells with and without sodium azide

The modification search was performed by specifying Cys-C_2H_4OH as a variable modification for samples that were treated with **VTT** without pre-incubation with NaN_3. For samples that were obtained after pre-treating cells with NaN_3 prior to addition of **VTT**, Cys-$C_2H_4N_3$ was defined as a variable modification.

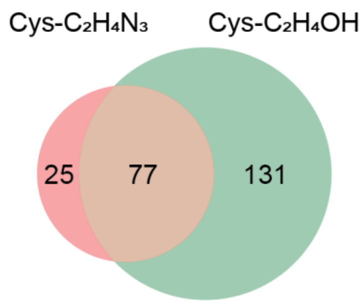

*Figure S29. Venn diagram with the numbers of unique modified peptides identified in all biological replicates after protein modification in E. coli cells without pre-incubation of the cells with sodium azide prior to addition of **VTT** (Cys-C_2H_4OH) or obtained from experiments where the cells were pre-incubated with sodium azide prior to addition of **VTT** (Cys-$C_2H_4N_3$).*

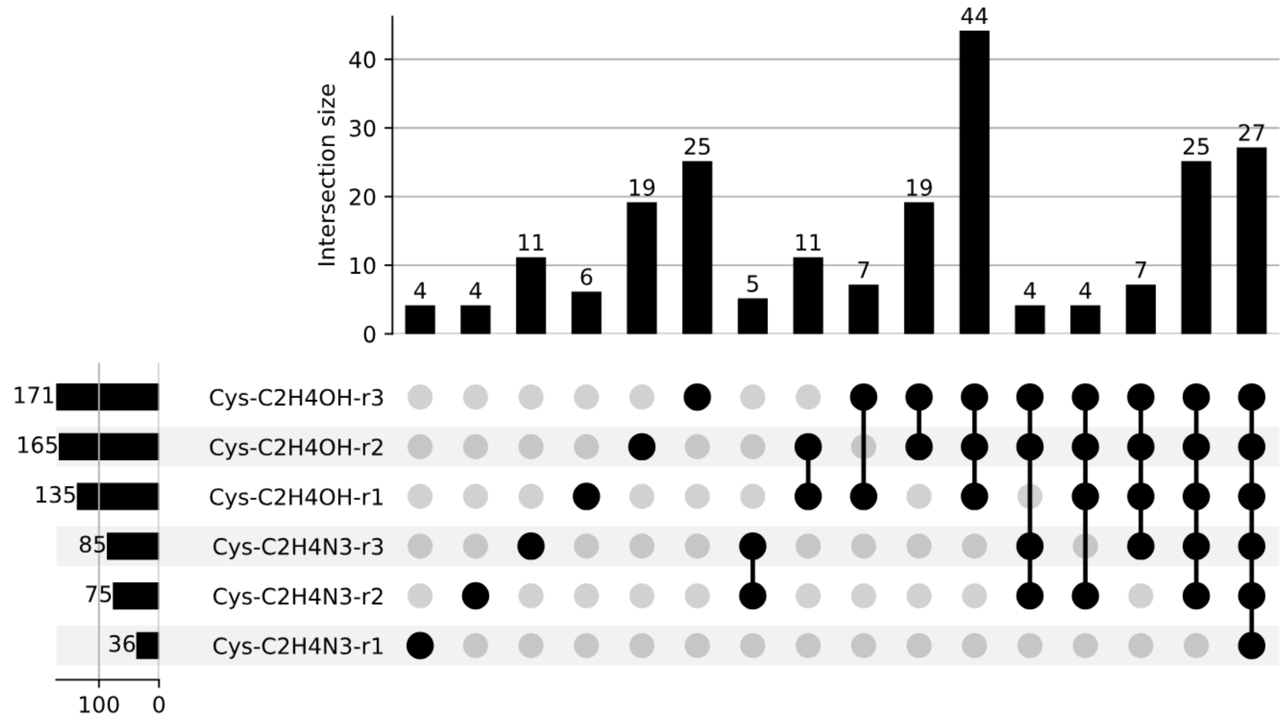

*Figure S30. Intersection analysis of modified peptides identified in the biological replicates (r1, r2, r3) after protein modification in E. coli cells without pre-incubation of the cells with sodium azide prior to addition of **VTT** (Cys-C_2H_4OH) or obtained from experiments with cells that were pre-incubated with sodium azide prior to addition of **VTT** (Cys-$C_2H_4N_3$). Each vertical bar represents a unique combination of input sets (indicated by filled dots in the matrix below the x-axis); the bar height equals the number of elements shared exclusively by that combination. Horizontal bars to the left show the total size of each individual set. Intersections containing fewer than three elements are omitted for clarity.*

Crosslink searches

In cellulo crosslinking with K562 cells

Raw data were processed using MSConvert 3.0.24[12] to convert the raw files to mzML format for the subsequent mass recalibration[13-15]. An open modification search with MSFragger 4.1[16] was then performed to recalibrate precursor masses, and the obtained files were converted to mgf format with MSConvert 3.0.24[12]. The mgf spectra were split for parallel database search *on the HPC system Raven at the Max Planck Computing and Data Facility*.

Each of the peakfiles was searched separately against the corresponding proteome (*H. sapiens*: UP000005640) using xiSEARCH 1.8.7[17] with the following settings: MS^1 tolerance: 3 ppm; MS^2 tolerance: 5 ppm[15], allowing up to two missing monoisotopic peaks and three missed tryptic cleavages. Cysteine carbamidomethylation and oxidation of methionine were defined as variable modifications. $–H_2O/–NH_3$ were defined as losses.

The **VTT** crosslinker was defined as follows: the linkage mass was set to 26.01565 Da and the specificity was set to C and one of the following residues: C, H, K, E, D, S, T, R, Y. The crosslink search for each of the considered crosslink combinations was performed separately. Variable modifications to account for the hydrolysis of episulfonium with water (18.0105647 Da), or formation of a crosslink within a peptide (26.01565 Da) were also defined. Non-covalent crosslink with a mass of zero was included in all searches to flag spectra potentially arising from gas-phase associated peptides[18].

The output files obtained for all the crosslink combinations of the same biological replicate were combined together. The matches obtained for the non-covalent crosslink were removed prior to false-discovery-rate (FDR) estimation, which was performed using xiFDR 2.3.5[17]. The input file was filtered to obtain peptide pairs with at least two matched fragment ions in each crosslinked peptide. The ambiguity on the residue pair and protein group levels was set to 1 and the results were filtered on residue pair level (1%) with the boosting feature enabled. The obtained files were uploaded to xiView.org[19] and the list of the residues in non-ambiguous crosslinks was obtained for each replicate:

Crosslinked AA	no. of identified crosslinks, replicate 1	no. of identified crosslinks, replicate 2	no. of identified crosslinks, replicate 3	relative AA frequency in the genome[20]	mean crosslink frequency, %	ratio between mean crosslink frequency and normalized AA frequency
Glu	101	102	102	15.9	20.4	1.3
Asp	84	85	84	10.6	16.9	1.6
Thr	87	82	79	12.1	16.6	1.4
Ser	79	87	78	18.7	16.3	0.9
Cys	66	67	56	5.2	12.7	2.4
Tyr	33	40	35	6.0	7.2	1.2
His	38	37	32	5.9	7.2	1.2
Lys	8	11	12	12.9	2.1	0.2
Arg	3	0	5	12.7	0.5	0.0

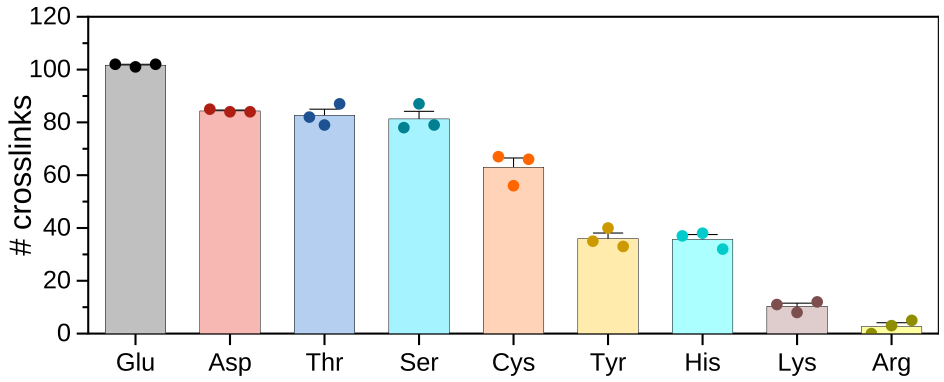

Figure S31. Number of the detected crosslinks from Cys to each of the searched amino acids. Data are means ± SE of n=3 independent experiments.

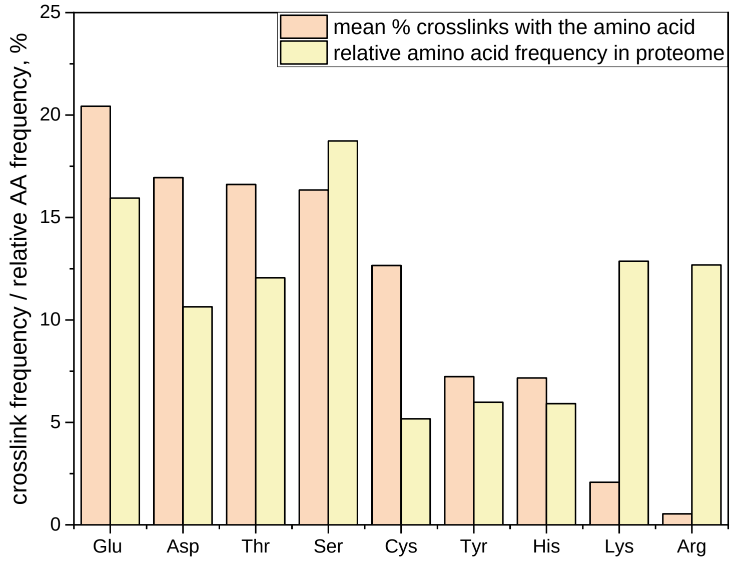

Figure S32. Mean relative frequencies of amino acids in the detected crosslinks and relative frequencies of the corresponding amino acids in the proteome.

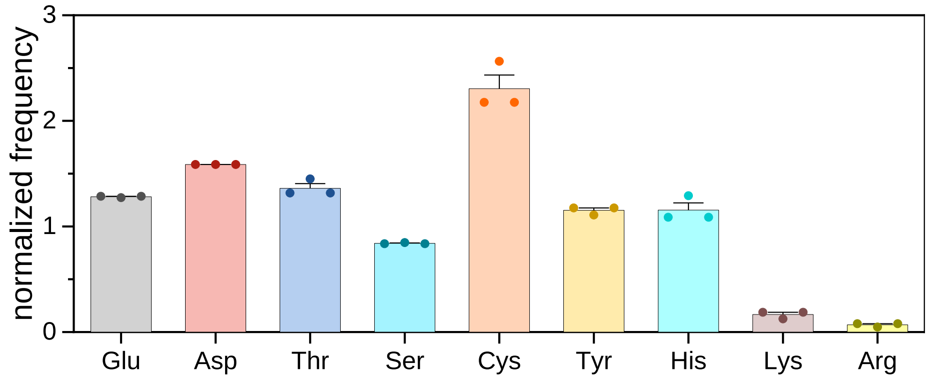

Figure S33. Normalized frequency of the identified crosslinks from Cys to each of the searched amino acids. Each Data are means ± SE of n=3 independent experiments.

Representative MS^2-spectra

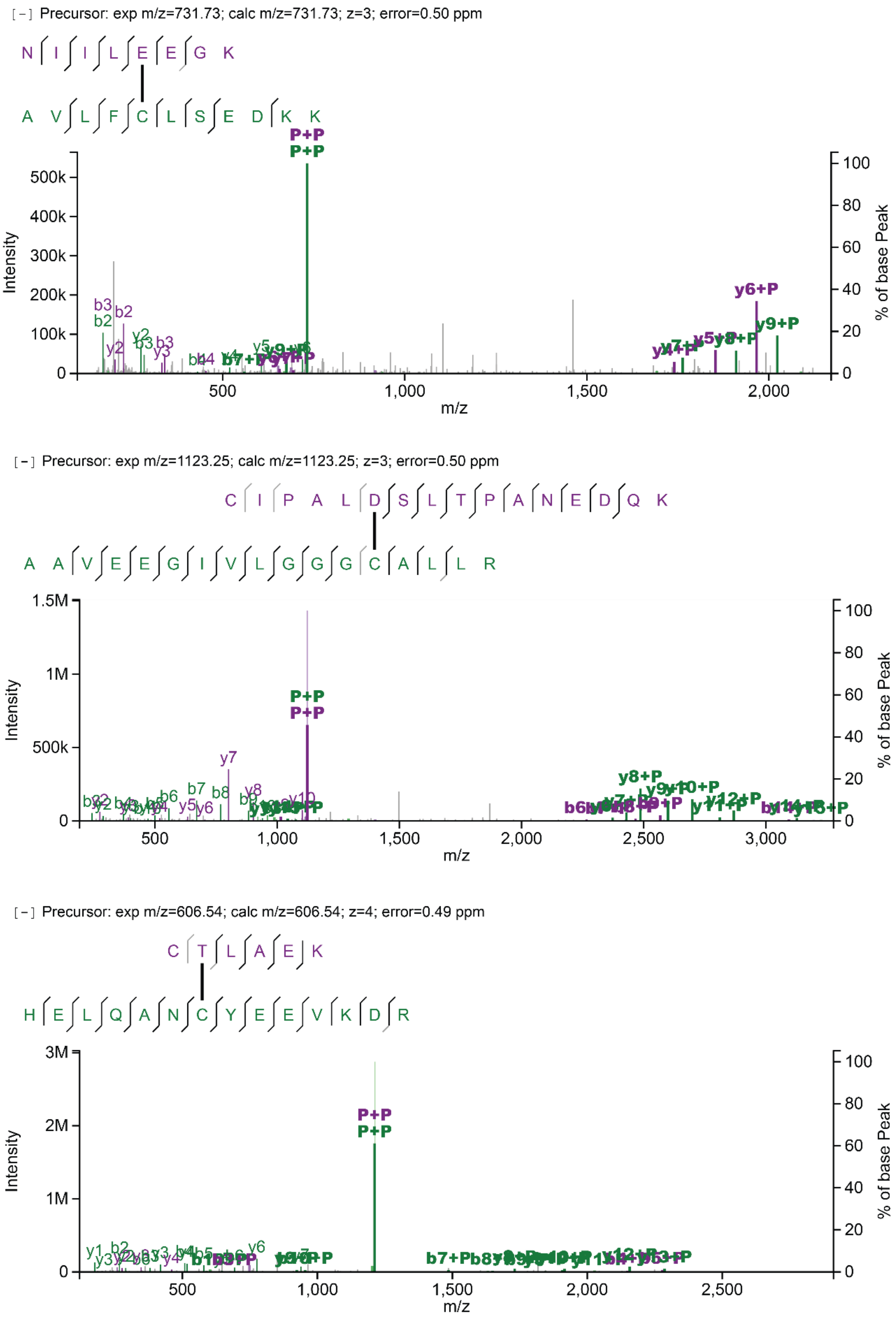

Figure S34. Representative MS^2-spectra of detected crosslinks between Cys and Glu, Asp, Thr.

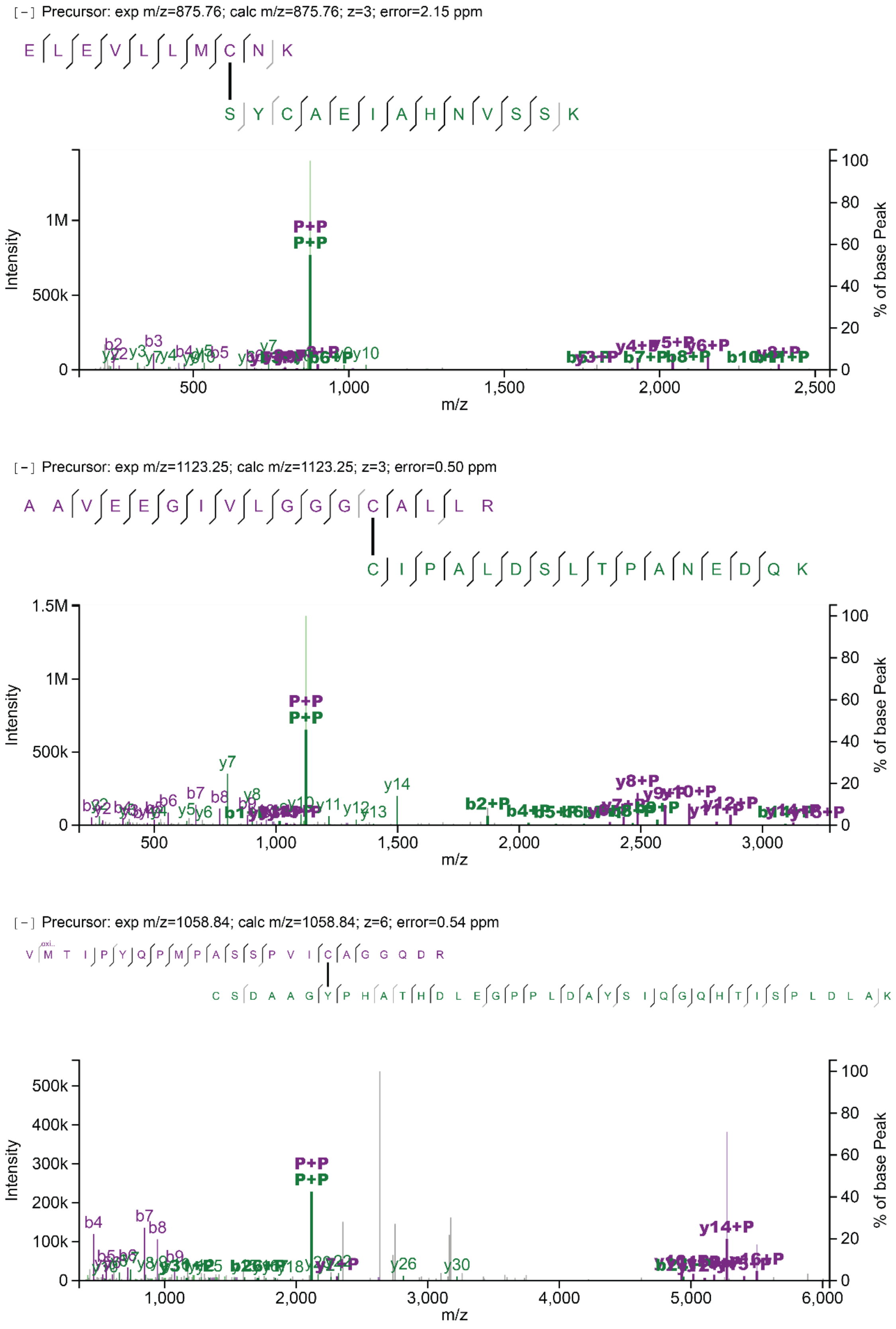

Figure S35. Representative MS^2-spectra of detected crosslinks between Cys and Ser, Cys, Tyr.

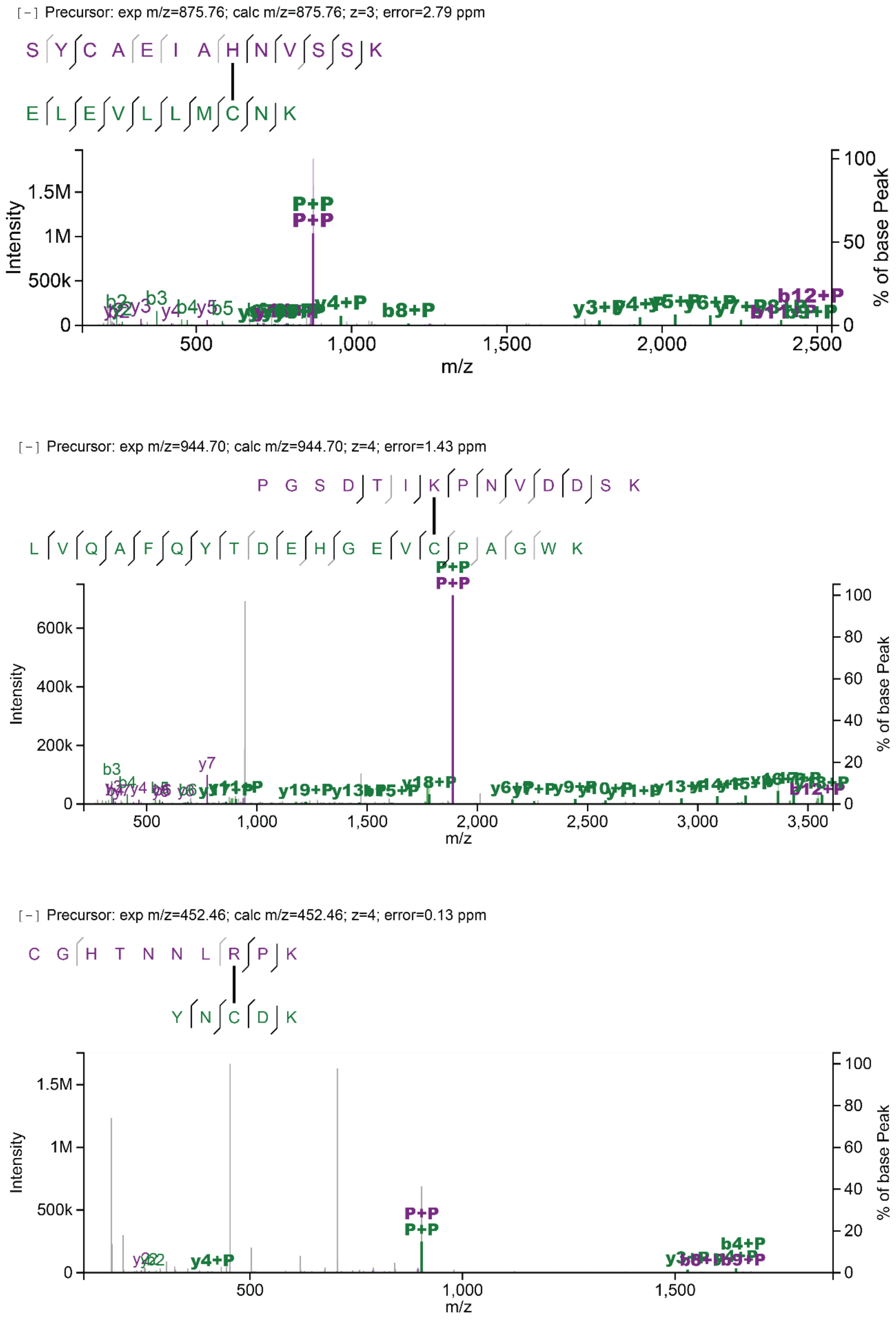

Figure S36. Representative MS²-spectra of detected crosslinks between Cys and His, Lys, Arg.

Crosslink distance distribution

For the analysis of the intraprotein crosslink distances, the crosslink pairs were filtered to obtain the list of the crosslinks that were detected in at least two biological replicates. Proteins that are known to form homooligomers were excluded from the analysis. High resolution X-ray structures with full sequence

coverage were used to obtain the Cα–Cα distances for the detected crosslinks. In cases where no such structure was available, AlphaFold[21]-predicted protein structures with predicted local distance difference test (pLDDT) score ≥ 80 were used for crosslink distance mapping.

The assignment of the secondary structure for the crosslinked residues was performed using PyMOL. The obtained assignments listed below as H (helix), S (sheet), and L (loop / coil):

Protein	Seq. Pos. 1	Seq. Pos. 2	Res. Name 1	Res. Name 2	Distance (Å)	Sec. Struct. 1	Sec. Struct. 2	Protein structure source
O00299	178	233	CYS	TYR	21.5	H	H	rcsb.org/structure/1K0M
O00299	24	59	CYS	CYS	18.3	L	L	rcsb.org/structure/1K0M
O00299	27	59	SER	CYS	15.9	H	L	rcsb.org/structure/1K0M
O00299	177	223	ASP	CYS	13.6	H	L	rcsb.org/structure/1K0M
O00299	178	221	CYS	SER	13.0	H	H	rcsb.org/structure/1K0M
O00299	178	223	CYS	CYS	11.5	H	L	rcsb.org/structure/1K0M
O00299	178	222	CYS	THR	10.6	H	H	rcsb.org/structure/1K0M
O00299	53	59	GLU	CYS	10.1	H	L	rcsb.org/structure/1K0M
O00299	178	218	CYS	GLU	9.3	H	H	rcsb.org/structure/1K0M
P00558	98	108	ASP	CYS	12.0	L	H	alphafold.com/entry/P00558
P00558	99	108	CYS	CYS	10.3	L	H	alphafold.com/entry/P00558
P00558	103	108	GLU	CYS	8.8	H	H	alphafold.com/entry/P00558
P07355	262	272	CYS	ASP	11.1	H	H	rcsb.org/structure/1W7B
P07355	262	269	CYS	TYR	5.9	H	H	rcsb.org/structure/1W7B
P08708	27	35	ASP	CYS	12.3	L	H	alphafold.com/entry/P08708
P08708	29	35	HIS	CYS	10.0	H	H	alphafold.com/entry/P08708
P08708	30	35	THR	CYS	8.7	H	H	alphafold.com/entry/P08708
P13639	388	462	CYS	GLU	19.6	L	L	alphafold.com/entry/P13639
P13639	370	388	GLU	CYS	13.5	H	L	alphafold.com/entry/P13639
P13639	373	388	TYR	CYS	13.5	L	L	alphafold.com/entry/P13639
P13639	369	389	CYS	ASP	12.6	H	L	alphafold.com/entry/P13639
P13639	369	388	CYS	CYS	9.8	H	L	alphafold.com/entry/P13639
P13639	387	466	SER	CYS	9.8	H	L	alphafold.com/entry/P13639
P13639	369	387	CYS	SER	9.5	H	H	alphafold.com/entry/P13639
P13639	388	466	CYS	CYS	6.0	L	L	alphafold.com/entry/P13639
P15880	176	188	LYS	CYS	30.7	L	S	alphafold.com/entry/P15880
P15880	178	229	HIS	CYS	28.9	L	L	alphafold.com/entry/P15880
P15880	178	188	HIS	CYS	25.6	L	S	alphafold.com/entry/P15880
P15880	221	229	ASP	CYS	24.5	L	L	alphafold.com/entry/P15880
P15880	182	229	CYS	CYS	24.5	L	L	alphafold.com/entry/P15880
P15880	182	230	CYS	THR	22.8	L	L	alphafold.com/entry/P15880
P15880	182	190	CYS	SER	22.6	L	L	alphafold.com/entry/P15880

P15880	179	188	THR	CYS	22.5	L	S	alphafold.com/entry/P15880
P15880	222	229	CYS	CYS	21.1	L	L	alphafold.com/entry/P15880
P15880	182	188	CYS	CYS	19.7	L	S	alphafold.com/entry/P15880
P15880	223	229	TYR	CYS	18.9	S	L	alphafold.com/entry/P15880
P15880	222	230	CYS	THR	18.2	L	L	alphafold.com/entry/P15880
P15880	225	229	SER	CYS	12.7	S	L	alphafold.com/entry/P15880
P15880	188	230	CYS	THR	10.5	S	L	alphafold.com/entry/P15880
P15880	188	229	CYS	CYS	10.3	S	L	alphafold.com/entry/P15880
P15880	190	229	SER	CYS	6.4	L	L	alphafold.com/entry/P15880
P17987	131	147	TYR	CYS	21.9	H	H	alphafold.com/entry/P17987
P17987	147	155	CYS	SER	12.5	H	H	alphafold.com/entry/P17987
P17987	68	76	GLU	CYS	11.1	L	H	alphafold.com/entry/P17987
P17987	69	76	HIS	CYS	10.8	L	H	alphafold.com/entry/P17987
P17987	147	154	CYS	THR	10.5	H	H	alphafold.com/entry/P17987
P17987	141	147	ASP	CYS	8.7	H	H	alphafold.com/entry/P17987
P17987	142	147	GLU	CYS	8.6	H	H	alphafold.com/entry/P17987
P17987	140	147	THR	CYS	6.4	H	H	alphafold.com/entry/P17987
P18077	37	47	ASP	CYS	19.2	H	S	alphafold.com/entry/P18077
P18077	40	47	GLU	CYS	14.8	H	S	alphafold.com/entry/P18077
P18077	39	47	THR	CYS	13.9	H	S	alphafold.com/entry/P18077
P18077	42	47	TYR	CYS	9.7	H	S	alphafold.com/entry/P18077
P21964	83	94	CYS	ASP	9.9	H	H	alphafold.com/entry/P21964
P21964	83	87	CYS	GLU	5.0	H	L	alphafold.com/entry/P21964
P23396	134	160	CYS	SER	15.4	S	L	alphafold.com/entry/P23396
P23396	134	159	CYS	HIS	11.9	S	L	alphafold.com/entry/P23396
P23396	129	134	SER	CYS	11.0	H	S	alphafold.com/entry/P23396
P23396	128	134	GLU	CYS	10.9	H	S	alphafold.com/entry/P23396
P23396	134	170	CYS	THR	9.9	S	S	alphafold.com/entry/P23396
P23396	134	154	CYS	ASP	5.5	S	S	alphafold.com/entry/P23396
P23528	39	66	CYS	ASP	23.0	S	L	rcsb.org/structure/5L6W
P23528	39	68	CYS	TYR	18.7	S	H	rcsb.org/structure/5L6W
P23528	39	70	CYS	THR	17.8	S	H	rcsb.org/structure/5L6W
P23528	139	147	CYS	CYS	15.4	L	H	rcsb.org/structure/5L6W
P23528	133	147	HIS	CYS	15.0	S	H	rcsb.org/structure/5L6W
P23528	140	147	TYR	CYS	14.0	H	H	rcsb.org/structure/5L6W
P23528	139	148	CYS	THR	13.4	L	H	rcsb.org/structure/5L6W
P23528	141	147	GLU	CYS	11.5	H	H	rcsb.org/structure/5L6W
P23528	144	147	LYS	CYS	8.8	H	H	rcsb.org/structure/5L6W
P23528	39	50	CYS	GLU	5.7	S	L	rcsb.org/structure/5L6W

P23528	145	147	ASP	CYS	5.5	H	H	rcsb.org/structure/5L6W
P31948	26	62	CYS	CYS	19.9	H	H	alphafold.com/entry/P31948
P31948	458	471	ASP	CYS	18.5	L	H	alphafold.com/entry/P31948
P31948	460	471	SER	CYS	18.0	L	H	alphafold.com/entry/P31948
P31948	26	59	CYS	GLU	17.8	H	H	alphafold.com/entry/P31948
P31948	27	62	TYR	CYS	16.2	H	H	alphafold.com/entry/P31948
P31948	28	62	SER	CYS	16.0	H	H	alphafold.com/entry/P31948
P31948	461	471	CYS	CYS	15.6	L	H	alphafold.com/entry/P31948
P31948	26	60	CYS	ASP	14.8	H	H	alphafold.com/entry/P31948
P31948	329	339	GLU	CYS	14.4	H	H	alphafold.com/entry/P31948
P31948	330	339	HIS	CYS	13.6	L	H	alphafold.com/entry/P31948
P31948	326	339	SER	CYS	10.0	H	H	alphafold.com/entry/P31948
P36578	245	250	HIS	CYS	14.1	L	S	alphafold.com/entry/P36578
P39023	313	336	SER	CYS	20.5	L	L	alphafold.com/entry/P39023
P39023	54	336	THR	CYS	9.9	S	L	alphafold.com/entry/P39023
P40939	322	333	CYS	THR	16.8	H	L	alphafold.com/entry/P40939
P40939	322	329	CYS	GLU	10.5	H	H	alphafold.com/entry/P40939
P43487	132	136	CYS	GLU	11.8	L	S	alphafold.com/entry/P43487
P46776	68	144	SER	CYS	22.3	H	S	alphafold.com/entry/P46776
P46776	70	144	CYS	CYS	17.4	L	S	alphafold.com/entry/P46776
P46776	76	144	ASP	CYS	15.5	H	S	alphafold.com/entry/P46776
P46776	72	144	THR	CYS	15.1	S	S	alphafold.com/entry/P46776
P49368	366	373	CYS	THR	11.8	L	S	alphafold.com/entry/P49368
P49368	367	372	LYS	CYS	11.8	L	S	alphafold.com/entry/P49368
P49368	365	372	ASP	CYS	10.4	L	S	alphafold.com/entry/P49368
P49368	366	372	CYS	CYS	9.4	L	S	alphafold.com/entry/P49368
P49368	364	372	THR	CYS	9.0	S	S	alphafold.com/entry/P49368
P50990	244	261	CYS	THR	32.4	L	L	alphafold.com/entry/P50990
P50990	244	263	CYS	GLU	31.5	L	H	alphafold.com/entry/P50990
P50991	252	267	CYS	SER	28.6	L	L	alphafold.com/entry/P50991
P50991	252	269	CYS	TYR	27.7	L	H	alphafold.com/entry/P50991
P50991	252	261	CYS	ASP	18.2	L	L	alphafold.com/entry/P50991
P50991	252	258	CYS	THR	13.4	L	L	alphafold.com/entry/P50991
P55769	93	103	CYS	SER	10.1	H	S	rcsb.org/structure/2OZB
P55769	93	102	CYS	CYS	8.2	H	S	rcsb.org/structure/2OZB
P60520	7	15	GLU	CYS	14.0	H	H	rcsb.org/structure/7LK3
P60520	8	15	ASP	CYS	13.4	H	H	rcsb.org/structure/7LK3
P60520	9	15	HIS	CYS	9.9	L	H	rcsb.org/structure/7LK3
P60520	10	15	SER	CYS	8.6	L	H	rcsb.org/structure/7LK3

P60981	133	147	HIS	CYS	12.7	S	H	alphafold.com/entry/P60981
P60981	134	147	GLU	CYS	12.3	S	H	alphafold.com/entry/P60981
P60981	135	147	CYS	CYS	9.5	S	H	alphafold.com/entry/P60981
P61247	89	96	GLU	CYS	7.7	S	S	alphafold.com/entry/P61247
P61247	90	96	ASP	CYS	6.1	S	S	alphafold.com/entry/P61247
P62241	160	174	SER	CYS	19.2	H	S	alphafold.com/entry/P62241
P62241	164	174	GLU	CYS	17.9	H	S	alphafold.com/entry/P62241
P62280	116	130	CYS	GLU	17.9	L	S	alphafold.com/entry/P62280
P62280	116	131	CYS	CYS	17.3	L	L	alphafold.com/entry/P62280
P62280	116	124	CYS	ASP	15.5	L	S	alphafold.com/entry/P62280
P62280	116	127	CYS	THR	14.3	L	S	alphafold.com/entry/P62280
P62280	114	131	SER	CYS	13.1	L	L	alphafold.com/entry/P62280
P62280	112	131	HIS	CYS	9.8	S	L	alphafold.com/entry/P62280
P62495	115	127	GLU	CYS	20.3	L	S	alphafold.com/entry/P62495
P62495	113	127	ASP	CYS	15.9	S	S	alphafold.com/entry/P62495
P62753	12	19	CYS	ASP	22.9	S	L	alphafold.com/entry/P62753
P62753	12	17	CYS	GLU	17.0	S	S	alphafold.com/entry/P62753
P62857	27	36	CYS	ASP	25.5	S	L	alphafold.com/entry/P62857
P62857	27	38	CYS	THR	24.3	S	L	alphafold.com/entry/P62857
P62857	27	33	CYS	GLU	18.7	S	S	alphafold.com/entry/P62857
P62888	85	107	CYS	SER	21.9	H	L	alphafold.com/entry/P62888
P62888	85	98	CYS	ASP	14.6	H	S	alphafold.com/entry/P62888
P62888	52	62	CYS	TYR	14.5	L	H	alphafold.com/entry/P62888
P62888	75	92	SER	CYS	11.5	L	L	alphafold.com/entry/P62888
P62888	52	59	CYS	GLU	11.1	L	H	alphafold.com/entry/P62888
P62888	85	92	CYS	CYS	11.0	H	L	alphafold.com/entry/P62888
P62888	85	93	CYS	THR	10.8	H	S	alphafold.com/entry/P62888
P62888	73	92	HIS	CYS	10.6	S	L	alphafold.com/entry/P62888
P62888	80	92	GLU	CYS	9.5	H	L	alphafold.com/entry/P62888
P62888	74	92	TYR	CYS	9.5	S	L	alphafold.com/entry/P62888
P62888	52	58	CYS	SER	9.5	L	H	alphafold.com/entry/P62888
P62888	52	93	CYS	THR	7.6	L	S	alphafold.com/entry/P62888
P62888	52	92	CYS	CYS	5.7	L	L	alphafold.com/entry/P62888
P62910	91	101	CYS	HIS	22.7	L	L	alphafold.com/entry/P62910
P62910	91	98	CYS	GLU	14.4	L	S	alphafold.com/entry/P62910
P62910	91	96	CYS	CYS	8.8	L	S	alphafold.com/entry/P62910
P62910	91	95	CYS	TYR	6.0	L	S	alphafold.com/entry/P62910
P62910	91	94	CYS	SER	5.4	L	H	alphafold.com/entry/P62910
P62913	72	80	CYS	GLU	14.4	S	H	alphafold.com/entry/P62913

P62987	91	115	CYS	CYS	22.3	H	L	alphafold.com/entry/P62987
P62987	91	117	CYS	HIS	21.9	H	L	alphafold.com/entry/P62987
P62987	92	115	ASP	CYS	21.5	L	L	alphafold.com/entry/P62987
P62987	91	118	CYS	THR	21.1	H	L	alphafold.com/entry/P62987
P63220	18	56	SER	CYS	8.4	L	L	alphafold.com/entry/P63220
P63220	17	56	CYS	CYS	5.4	L	L	alphafold.com/entry/P63220
P83731	11	36	TYR	CYS	10.2	S	H	alphafold.com/entry/P83731
P83731	6	37	CYS	GLU	9.7	S	H	alphafold.com/entry/P83731
P83731	7	36	SER	CYS	8.4	L	H	alphafold.com/entry/P83731
P83731	6	36	CYS	CYS	8.0	S	H	alphafold.com/entry/P83731
Q02543	88	109	SER	CYS	20.9	L	H	alphafold.com/entry/Q02543
Q02543	90	109	THR	CYS	17.1	S	H	alphafold.com/entry/Q02543
Q02543	91	109	HIS	CYS	15.2	S	H	alphafold.com/entry/Q02543
Q13347	76	82	CYS	GLU	18.7	S	L	alphafold.com/entry/Q13347
Q13347	75	81	SER	CYS	18.2	S	L	alphafold.com/entry/Q13347
Q13347	76	83	CYS	THR	18.2	S	L	alphafold.com/entry/Q13347
Q13347	76	81	CYS	CYS	15.2	S	L	alphafold.com/entry/Q13347
Q13347	76	80	CYS	ASP	13.8	S	S	alphafold.com/entry/Q13347
Q15185	40	57	CYS	HIS	24.7	S	L	alphafold.com/entry/Q15185
Q15185	40	58	CYS	CYS	23.9	S	L	alphafold.com/entry/Q15185
Q15185	40	60	CYS	ASP	22.6	S	L	alphafold.com/entry/Q15185
Q15185	39	58	SER	CYS	20.6	S	L	alphafold.com/entry/Q15185
Q15185	37	58	THR	CYS	14.1	S	L	alphafold.com/entry/Q15185
Q15185	40	52	CYS	GLU	12.4	S	S	alphafold.com/entry/Q15185
Q16658	57	89	SER	CYS	13.4	L	H	alphafold.com/entry/Q16658
Q16658	61	88	CYS	ASP	9.4	S	H	alphafold.com/entry/Q16658
Q16658	61	89	CYS	CYS	5.7	S	H	alphafold.com/entry/Q16658
Q8N1F7	391	397	TYR	CYS	7.7	H	L	alphafold.com/entry/Q8N1F7
Q8N1F7	392	398	CYS	ASP	6.3	H	L	alphafold.com/entry/Q8N1F7
Q8N1F7	392	397	CYS	CYS	4.9	H	L	alphafold.com/entry/Q8N1F7
Q99832	370	385	CYS	THR	16.9	S	H	alphafold.com/entry/Q99832
Q99832	370	386	CYS	GLU	14.4	S	H	alphafold.com/entry/Q99832
Q99832	349	364	GLU	CYS	13.1	S	L	alphafold.com/entry/Q99832
Q99832	345	362	CYS	THR	6.2	L	S	alphafold.com/entry/Q99832
Q99832	345	364	CYS	CYS	4.5	L	L	alphafold.com/entry/Q99832
Q9BZZ5	234	241	CYS	CYS	10.7	H	H	alphafold.com/entry/Q9BZZ5
Q9BZZ5	236	241	ASP	CYS	8.4	H	H	alphafold.com/entry/Q9BZZ5
Q9NTK5	68	75	ASP	CYS	10.9	L	H	alphafold.com/entry/Q9NTK5
Q9NTK5	69	75	GLU	CYS	9.7	H	H	alphafold.com/entry/Q9NTK5

Q9UIA9	42	47	ASP	CYS	8.6	L	H	alphafold.com/entry/Q9UIA9
Q9UIA9	43	47	CYS	CYS	6.0	H	H	alphafold.com/entry/Q9UIA9
Q9UIA9	45	47	SER	CYS	5.5	H	H	alphafold.com/entry/Q9UIA9
Q9UQ80	49	58	CYS	GLU	14.3	H	H	alphafold.com/entry/Q9UQ80
Q9UQ80	49	53	CYS	ASP	6.2	H	H	alphafold.com/entry/Q9UQ80
Q9Y2R5	96	100	THR	CYS	9.8	L	L	alphafold.com/entry/Q9Y2R5
Q9Y2R5	93	100	ASP	CYS	6.3	L	L	alphafold.com/entry/Q9Y2R5
Q9Y696	234	246	CYS	ASP	20.8	L	H	alphafold.com/entry/Q9Y696
Q9Y696	234	245	CYS	SER	18.2	L	H	alphafold.com/entry/Q9Y696
Q9Y696	234	244	CYS	TYR	15.5	L	H	alphafold.com/entry/Q9Y696
Q9Y696	234	239	CYS	GLU	9.9	L	H	alphafold.com/entry/Q9Y696

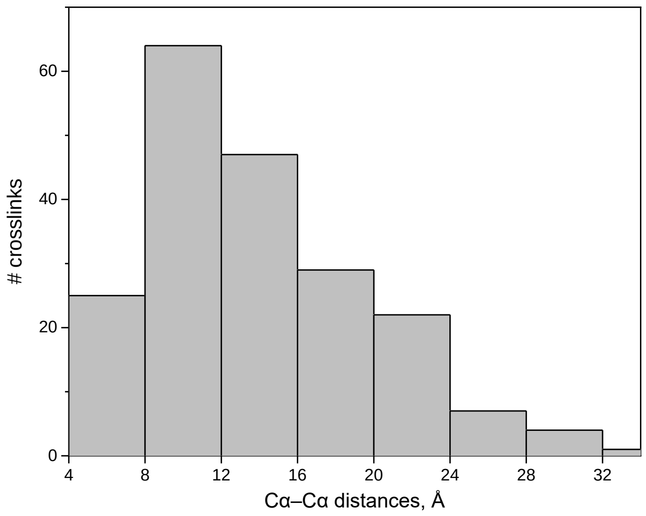

Figure S37. Distribution of the mapped crosslink distances.

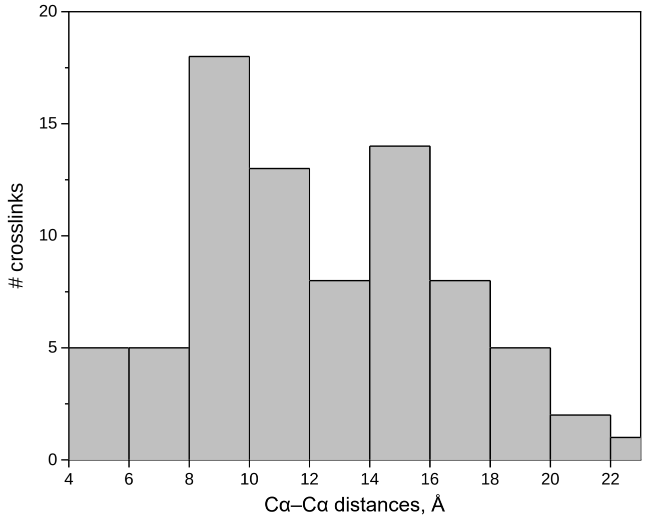

Figure S38. Distribution of the mapped crosslink distances after excluding crosslinks between residues assigned to non-regular secondary structure elements (loops/coils).

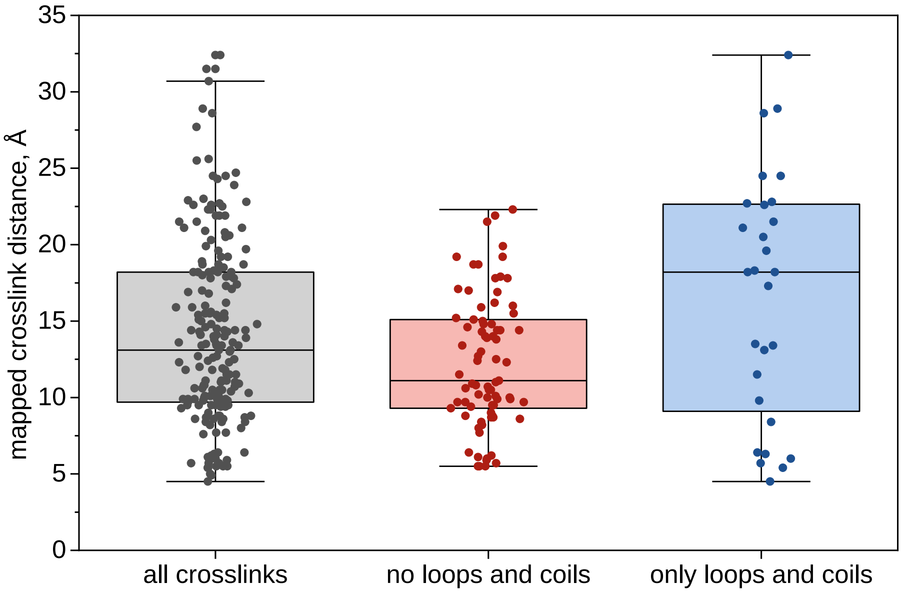

Figure S39. Distribution of the mapped crosslink distances (all crosslinks), distances obtained after excluding crosslinks to residues assigned to non-regular secondary structure elements (no loops and coils), and distances obtained for crosslinks between residues assigned to non-regular secondary structure elements (only loops and coils).

***In cellulo* crosslinking with *S. cerevisiae* cells**

Raw data were processed using MSConvert 3.0.24[12] to convert the raw files to mzML format for the subsequent mass recalibration[13-15]. An open modification search with MSFragger 4.1[16] was then performed to recalibrate precursor masses, and the obtained files were converted to mgf format with MSConvert 3.0.24[12]. The database search was performed *on the HPC system Raven at the Max Planck Computing and Data Facility*.

Each of the peak files was searched separately against the corresponding proteome (*S. cerevisiae:* UP000002311) using xiSEARCH 1.8.7[17] with the following settings: MS^1 tolerance: 3 ppm; MS^2 tolerance: 5 ppm[15], allowing up to two missing monoisotopic peaks and three missed tryptic cleavages. Cysteine carbamidomethylation and oxidation of methionine were defined as variable modifications. $–H_2O/–NH_3$ were defined as losses.

The **VTT** crosslinker was defined as follows: the linkage mass was set to 26.01565 Da and the specificity was set to C and one of the following residues: C, H, K, E, D, S, T, R, Y, C-terminus, N-terminus. Variable modifications to account for the hydrolysis of episulfonium with water (18.0105647 Da), or formation of a crosslink within a peptide (26.01565 Da) were also defined. Non-covalent crosslink with a mass of zero was included in all searches to flag spectra potentially arising from gas-phase associated peptides[18].

The matches obtained for the non-covalent crosslink were removed prior to the FDR estimation, which was performed using xiFDR 2.3.5[17]. The input file was filtered to obtain peptide pairs with at least two matched fragment ions in each crosslinked peptide. The ambiguity on the residue pair and protein group levels was set to 1 and the results were filtered on residue pair level (1%) with the boosting feature enabled.

The obtained files were uploaded to xiView.org[19] and the data were filtered to obtain the list of the crosslinks that were detected in at least two biological replicates. The filtered data for protein with the

highest number of identified crosslinks were used as an input for protein structure prediction with AlphaLink2[22] using ColabFold with the following parameters: model weights: 2.2 (trained on 10 Å Cα-Cα); use templates: True; msa mode: MMseqs2; max recycling iters: 3; num ensembles: 1; manual seed: 42; times: 1.

The obtained structure with the detected crosslinks was visualized using ChimeraX 1.9[23].

In cellulo crosslinking with HEK cells

Raw data were processed using MSConvert 3.0.24[12] to convert the raw files to mzML format for the subsequent mass recalibration[13-15]. An open modification search with MSFragger 4.1[16] was then performed to recalibrate precursor masses, and the obtained files were converted to mgf format with MSConvert 3.0.24[12]. The mgf spectra were split for parallel database search *on the HPC system Raven at the Max Planck Computing and Data Facility*.

Each of the peak files was searched separately against the corresponding proteome (*H. sapiens*: UP000005640) using xiSEARCH 1.8.7[17] with the following settings: MS^1 tolerance: 3 ppm; MS^2 tolerance: 5 ppm[15], allowing up to two missing monoisotopic peaks and three missed tryptic cleavages. Cysteine carbamidomethylation and oxidation of methionine were defined as variable modifications. $–H_2O/–NH_3$ were defined as losses.

The **VTT** crosslinker was defined as follows: the linkage mass was set to 26.01565 Da and the specificity was set to C and one of the following residues: C, H, K, E, D, S, T, R, Y, C-terminus, N-terminus. The crosslink search for each of the considered crosslink combinations was performed separately. Variable modifications to account for the hydrolysis of episulfonium with water (18.0105647 Da), or formation of a crosslink within a peptide (26.01565 Da) were also defined. Non-covalent crosslink with a mass of zero was included in all searches to flag spectra potentially arising from gas-phase associated peptides[18].

The output files obtained for all the crosslink combinations of the same biological replicate were combined together. The matches obtained for the non-covalent crosslink were removed prior to the FDR estimation, which was performed using xiFDR 2.3.5[17]. The input file was filtered to obtain peptide pairs with at least two matched fragment ions in each crosslinked peptide. The ambiguity on the residue pair and protein group levels was set to 1 and the results were filtered on residue pair level (1%) with the boosting feature enabled.

The obtained files were uploaded to xiView.org[19] and the data were filtered to obtain the list of the crosslinks that were detected in at least two biological replicates. The filtered data for protein with the highest number of identified crosslinks were used as an input for protein structure prediction with AlphaLink2[22] using ColabFold with the following parameters: model weights: 2.2 (trained on 10 Å Cα-Cα); use templates: True; msa mode: MMseqs2; max recycling iters: 3; num ensembles: 1; manual seed: 42; times: 1.

The obtained structure with the detected crosslinks was visualized using ChimeraX 1.9[23].

In cellulo crosslinking with *A. thaliana* cells

Raw data were processed using MSConvert 3.0.24[12] to convert the raw files to mzML format for the subsequent mass recalibration[13-15]. An open modification search with MSFragger 4.1[16] was then performed to recalibrate precursor masses, and the obtained files were converted to mgf format with

MSConvert 3.0.24[12]. The mgf spectra were split for parallel database search *on the HPC system Raven at the Max Planck Computing and Data Facility*.

Each of the peak files was searched separately against the corresponding proteome (*A. thaliana:* UP000006548) using xiSEARCH 1.8.7[17] with the following settings: MS^1 tolerance: 3 ppm; MS^2 tolerance: 5 ppm[15], allowing up to two missing monoisotopic peaks and three missed tryptic cleavages. Cysteine carbamidomethylation and oxidation of methionine were defined as variable modifications. $–H_2O/–NH_3$ were defined as losses.

The **VTT** crosslinker was defined as follows: the linkage mass was set to 26.01565 Da and the specificity was set to C and one of the following residues: C, H, K, E, D, S, T, R, Y, C-terminus, N-terminus. The crosslink search for each of the considered crosslink combinations was performed separately. Variable modifications to account for the hydrolysis of episulfonium with water (18.0105647 Da), or formation of a crosslink within a peptide (26.01565 Da) were also defined. Non-covalent crosslink with a mass of zero was included in all searches to flag spectra potentially arising from gas-phase associated peptides[18].

The output files obtained for all the crosslink combinations of the same biological replicate were combined together. The matches obtained for the non-covalent crosslink were removed prior to the FDR estimation, which was performed using xiFDR 2.3.5[17]. The input file was filtered to obtain peptide pairs with at least three matched fragment ions in each crosslinked peptide. The ambiguity on the residue pair and protein group levels was set to 1 and the results were filtered on residue pair level (1%) with the boosting feature enabled.

The obtained files were uploaded to xiView.org[19] and the data were filtered to obtain the list of the crosslinks that were detected in at least two biological replicates. The filtered data for protein with the highest number of identified crosslinks were used as an input for protein structure prediction with AlphaLink2[22] using ColabFold with the following parameters: model weights: 2.2 (trained on 10 Å Cα-Cα); use templates: True; msa mode: MMseqs2; max recycling iters: 3; num ensembles: 1; manual seed: 42; times: 1.

The obtained structure with the detected crosslinks was visualized using ChimeraX 1.9[23].

In cellulo crosslinking with *B. subtilis* cells

Raw data were processed using MSConvert 3.0.24[12] to convert the raw files to mzML format for the subsequent mass recalibration[13-15]. An open modification search with MSFragger 4.1[16] was then performed to recalibrate precursor masses, and the obtained files were converted to mgf format with MSConvert 3.0.24[12]. The database search was performed *on the HPC system Raven at the Max Planck Computing and Data Facility*.

Each of the peak files was searched separately against the corresponding proteome (*B. subtilis:* UP000001570) using xiSEARCH 1.8.7[17] with the following settings: MS^1 tolerance: 3 ppm; MS^2 tolerance: 5 ppm[15], allowing up to two missing monoisotopic peaks and three missed tryptic cleavages. Cysteine carbamidomethylation and oxidation of methionine were defined as variable modifications. $–H_2O/–NH_3$ were defined as losses.

The **VTT** crosslinker was defined as follows: the linkage mass was set to 26.01565 Da and the specificity was set to C and one of the following residues: C, H, K, E, D, S, T, R, Y, C-terminus, N-terminus. Variable

modifications to account for the hydrolysis of episulfonium with water (18.0105647 Da), or formation of a crosslink within a peptide (26.01565 Da) were also defined. Non-covalent crosslink with a mass of zero was included in all searches to flag spectra potentially arising from gas-phase associated peptides[18].

The matches obtained for the non-covalent crosslink were removed prior to the FDR estimation, which was performed using xiFDR 2.3.5[17]. The input file was filtered to obtain peptide pairs with at least three matched fragment ions in each crosslinked peptide. The ambiguity on the residue pair and protein group levels was set to 1 and the results were filtered on residue pair level (1%) with the boosting feature enabled.

The obtained files were uploaded to xiView.org[19] and the data were filtered to obtain the list of the crosslinks that were detected in at least two biological replicates. The filtered data for protein with the highest number of identified crosslinks were used as an input for protein structure prediction with AlphaLink2[22] using ColabFold with the following parameters: model weights: 2.2 (trained on 10 Å Cα-Cα); use templates: True; msa mode: MMseqs2; max recycling iters: 3; num ensembles: 1; manual seed: 42; times: 1.

The obtained structure with the detected crosslinks was visualized using ChimeraX 1.9[23].

In cellulo crosslinked GrxC5

Raw data were converted to mgf format with MSConvert 3.0.24[12].

Each of the peak files was searched separately against the protein FASTA sequence using xiSEARCH 1.8.7[17] with the following settings: MS^1 tolerance: 6 ppm; MS^2 tolerance: 20 ppm[15], allowing up to two missing monoisotopic peaks and three missed tryptic cleavages. Cysteine carbamidomethylation and oxidation of methionine were defined as variable modifications. $–H_2O/–NH_3$ were defined as losses.

The **VTT** crosslinker was defined as follows: the linkage mass was set to 26.01565 Da and the specificity was set to C and one of the following residues: C, H, K, E, D, S, T, R, Y, C-terminus, N-terminus. Variable modifications to account for the hydrolysis of episulfonium with water (18.0105647 Da), or formation of a crosslink within a peptide (26.01565 Da) were also defined. Non-covalent crosslink with a mass of zero was included in all searches to flag spectra potentially arising from gas-phase associated peptides[18].

The matches obtained for the non-covalent crosslink were removed prior to the FDR estimation, which was performed using xiFDR 2.3.5[17]. The input file was filtered to obtain peptide pairs with at least two matched fragment ions in each crosslinked peptide. The ambiguity on the residue pair level was set to 1 and the results were filtered on residue pair level (1%) with the boosting feature enabled.

The obtained files were uploaded to xiView.org[19] and the crosslink pairs found in at least two of the three biological replicates were used as an input for protein structure prediction with AlphaLink2[22] using ColabFold with the following parameters: model weights: 2.2 (trained on 10 Å Cα-Cα); use templates: True; msa mode: MMseqs2; max recycling iters: 3; num ensembles: 1; manual seed: 42; times: 1.

The obtained structure with the detected crosslinks was visualized using ChimeraX 1.9[23].

Crosslinker comparison

Crosslinking reagents used for comparison

Commercially available Cys-reactive crosslinking reagents were considered for the analysis. Reagents with PEG linkers or sulfo-derivatives were not included in the analysis due to the low cell permeability of these compounds.

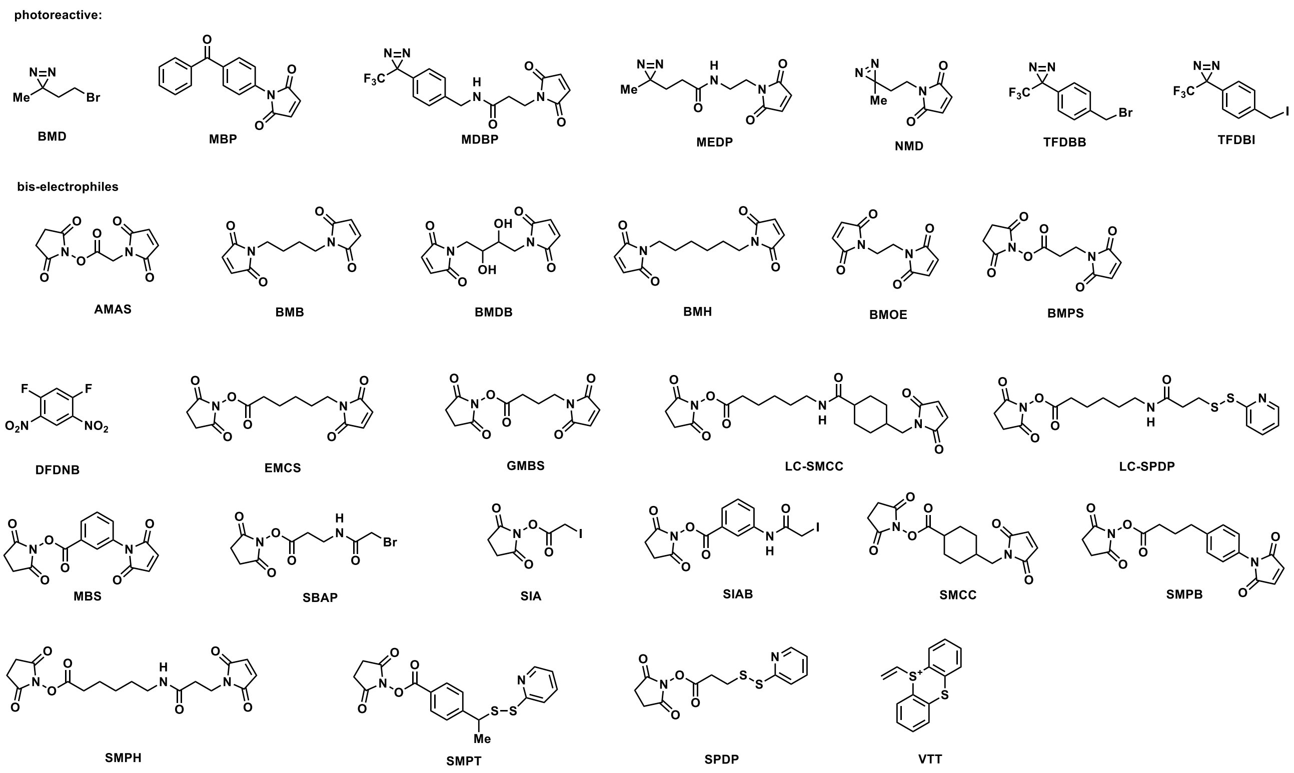

Figure S40. Structures of the Cys-reactive crosslinking reagents used for the analysis.

Calculated TPSA and logP

The TPSA and XlogP values for **VTT** and other Cys-selective crosslinking reagents were calculated using SwissADME[24]. The listed linker distances were previously reported[25] or calculated using Avogadro[26].

Abbreviation	Name	Spacer arm, Å^2	TPSA,	XLOGP3
BMD	3-(2-Bromoethyl)-3-methyl-3H-diazirine	2.6	24.72	1.63
MBP	4-(N-Maleimido)benzophenone	8.1	51.21	3
MDBP	3-(Maleimido)-N-(4-(3-(trifluoromethyl)-3H-diazirin-3-yl)benzyl)propanamide	12.3	91.2	1.22
MEDP	N-(2-(Maleimido)ethyl)-3-(3-methyl-3H-diazirin-3-yl)propanamide	10.8	91.2	-0.63
NMD	N-maleimido-dizirine	6	62.1	0.12
TFDBB	4-[3-(Trifluoromethyl)-3H-diazirin-3-yl]benzyl Bromide	5.9	24.72	3.48
TFDBI	4-[3-(Trifluoromethyl)-3H-diazirin-3-yl]benzyl Iodide	5.9	24.72	3.93
AMAS	N -(α-Maleimidoacetoxy)-succinimide ester	4.4	101.06	-1.48
BMB	1,4-Bis-Maleimidobutane	10.9	74.76	-0.55
BMDB	1,4-Bis-Maleimmidyl-2,3-dihydroxy-butane	10.2	115.22	-2.5
BMH	Bis-Maleimidohexane	16.1	74.76	0.16
BMOE	Bis-Maleimidoethane	8	74.76	-1.26
BMPS	N -(β-Maleimidopropyloxy)succinimide ester	5.9	101.06	-1.58

DFDNB	1-5-Difluoro-2,4-dinitrobenzene	3	91.64	1.78
EMCS	N-(ε-Maleimidocaproyloxy)succinimide ester	11.8	101.06	-0.51
GMBS	N-(γ-Maleimidobutyryloxy)succinimide ester	7.3	101.06	-1.22
LC-SMCC	Succinimidyl 4-(N-maleimidomethyl) cyclohexane-1-carboxy-(6-amidocaproate)	16.2	130.16	0.23
LC-SPDP	Succinimidyl 6-(3'-[2-pyridyl-dithio]propionamido)hexanoate	15.7	156.27	0.99
MBS	m-Maleimidobenzoyl-N-hydroxysuccinimide ester	7.3	101.06	0.01
SBAP	Succinimdyl 3-(bromoacetamido)propionate	6.2	92.78	-0.73
SIA	N-succinimidyl iodoacetate	1.5	63.68	-0.09
SIAB	N-Succinimidyl(4-iodoacetyl)aminobenzoate	10.6	92.78	0.75
SMCC	Succinimidyl 4-(N-maleimido-methyl)cyclohexane-1-carboxylate	8.3	101.06	-0.09
SMPB	Succinimidyl 4-(p-maleimido-phenyl)butyrate	11.6	101.06	0.59
SMPH	Succinimidyl-6-(β-maleimidopropionamido)hexanoate	14.2	130.16	-1.26
SMPT	4-Succinimidyloxycarbonyl-methyl-α-(2-pyridyldithio)toluene	20	127.17	2.59
SPDP	N-Succinimidyl 3-(2-pyridyldithio)propionate	6.8	127.17	0.67
VTT	Vinylthianthrenium tetrafluoroborate	1.5	50.6	4.64

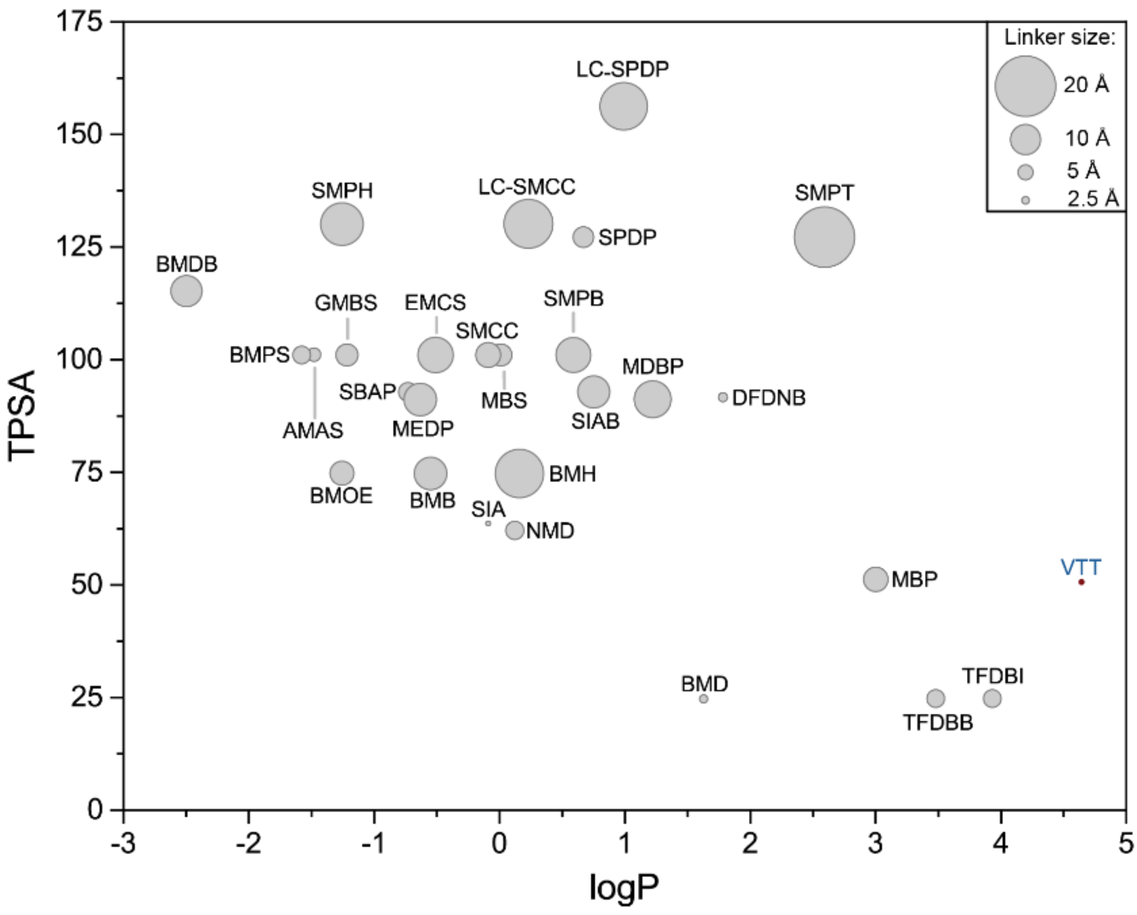

*Figure S41. Comparison of **VTT** with other crosslinking reagents based on the calculated topological polar surface area (TPSA), the octanol-water partition coefficient (P), and liker size. High TPSA values correlate with lower cell permeability and low logP values indicate higher affinity towards water[27, 28].*

Avogardo outputs

Geometry optimization was performed using the following settings: Force field: MMFF94; Number of steps: 25000.

TFDBB and **TFDBI**-derived crosslinks. Distance between C7 and C8: 5.9 Å

C	-1.00159	2.68253	-0.42266
C	-0.62351	4.00200	-0.13536
C	-0.04230	1.81543	-0.96607
C	0.67709	4.44429	-0.39183
C	1.25800	2.25832	-1.22252
C	1.62917	3.57994	-0.94614
H	-1.33310	4.70516	0.29699
H	-0.29348	0.78113	-1.19259
H	1.98426	1.56061	-1.63444
H	0.94464	5.47069	-0.14890
C	3.03380	4.05556	-1.21513
C	-2.40843	2.17423	-0.14559
C	-2.71602	2.20309	1.35485
H	-1.96068	1.64610	1.92057
H	-3.69001	1.74539	1.55882
H	-2.74007	3.22531	1.74794
C	-3.48668	2.91571	-0.93693
F	-3.60628	4.20935	-0.56482
F	-4.69636	2.33317	-0.74654
F	-3.24317	2.88571	-2.26660
H	-2.45842	1.12294	-0.45940
S	4.12203	3.77290	0.21422
H	3.02201	5.12461	-1.45591
H	3.43883	3.53235	-2.08875
C	5.64842	4.45175	-0.47504
H	6.44769	4.36634	0.26591
H	5.51749	5.50800	-0.72413
H	5.94314	3.89610	-1.36906

MBP-derived crosslink. Distance between C7 and C17: 8.1 Å

C	-0.87668	2.88839	-0.11585
C	-0.50924	4.16599	0.33422
C	0.13652	2.12152	-0.71003
C	0.79980	4.65425	0.19588
C	1.44645	2.60638	-0.85223
C	1.80477	3.88156	-0.39554
H	-1.21516	4.82845	0.82681
H	-0.04721	1.12556	-1.10146
H	2.18682	1.96261	-1.32488
H	1.02145	5.65566	0.56228
C	3.23480	4.40948	-0.57066
C	3.41065	4.83331	-2.03961
H	3.30705	3.99266	-2.73477
H	4.38983	5.29975	-2.20263
H	2.66603	5.58930	-2.31982
O	3.39807	5.62425	0.18540
H	4.33122	5.66802	0.45476
C	4.29200	3.42412	-0.05188
C	5.27964	2.85291	-0.87126
C	4.28703	3.07142	1.31374
C	5.22930	2.18422	1.83667
C	6.19821	1.63129	1.00569
C	6.22333	1.96474	-0.34586
H	5.33637	3.08059	-1.93242
H	3.53271	3.49053	1.97937
H	5.20211	1.92362	2.89157

H	6.93130	0.93831	1.41002
H	6.97902	1.53030	-0.99590
C	-6.45098	-0.31594	0.58729
S	-4.66628	-0.45741	0.84919
H	-6.96865	-0.73027	1.42708
H	-6.72479	-0.84920	-0.29903
H	-6.71493	0.71532	0.47894
C	-4.48326	2.29653	0.63157
C	-4.11610	1.01496	-0.07288
O	-1.92119	0.12529	-0.51084
C	-2.60977	1.08820	-0.19637
H	-4.59632	0.91852	-1.02419
O	-3.38199	4.39084	0.50075
C	-3.29780	3.17531	0.38433
H	-4.62626	2.13108	1.67898
H	-5.40032	2.72067	0.27940
N	-2.21589	2.39565	0.03351

BMD-derived crosslink. Distance between C2 and C5: 2.6 Å

C	-2.04756	-0.21479	1.63221
C	-0.64957	-0.83007	1.73467
H	-2.19179	0.57149	2.38108
H	-2.82020	-0.97558	1.78614
H	-2.20777	0.22563	0.64240
C	0.41760	0.24819	1.46063
C	-0.47596	-1.48975	3.10358
H	-0.56852	-1.60865	0.96553
H	-1.28676	-2.20039	3.29754
H	-0.48062	-0.74506	3.90665
H	0.46302	-2.04836	3.16093
C	1.83777	-0.31597	1.40186
H	0.35702	1.02575	2.23364
H	0.18067	0.73154	0.50325
S	3.01043	0.99777	0.93356
H	1.89438	-1.12075	0.66195
H	2.13852	-0.71005	2.37668
C	4.53148	0.01969	0.94978
H	5.37530	0.66145	0.68291
H	4.46922	-0.79318	0.22163
H	4.70927	-0.39134	1.94690

MEDP-derived crosslink. Distance between C2 and C11: 10.8 Å

C	-2.05883	-0.17403	1.65572
C	-0.67609	-0.82689	1.72434
H	-2.18144	0.58133	2.43944
H	-2.84929	-0.92203	1.77888
H	-2.21142	0.31384	0.68716
C	0.41511	0.23519	1.49798
C	-0.51410	-1.55241	3.06091
H	-0.61617	-1.57089	0.91994
H	-1.33894	-2.25464	3.22378
H	-0.50273	-0.84556	3.89747
H	0.41366	-2.13154	3.09048
C	1.82288	-0.35274	1.39108
H	0.38839	0.97664	2.30715
H	0.19061	0.78306	0.57315
C	2.85990	0.70115	1.04480
H	1.84399	-1.12469	0.61304
H	2.12115	-0.80901	2.34107
O	2.60967	1.89502	0.93338
N	4.12608	0.17056	0.88633

C	5.22393	1.00887	0.43503
H	4.18886	-0.82683	0.72376
C	6.58986	0.40307	0.76599
H	5.13126	2.00341	0.88691
H	5.10770	1.13463	-0.64772
N	7.67946	1.19773	0.23097
H	6.65792	-0.60666	0.34375
H	6.70418	0.31687	1.85268
C	8.12959	1.08902	-1.04983
C	8.31934	2.17968	0.93200
C	9.29030	2.88242	0.00709
C	9.29196	2.01600	-1.24526
H	10.20362	1.41042	-1.29349
H	9.18167	2.57064	-2.18104
O	8.13807	2.45377	2.10973
O	7.70397	0.32562	-1.90369
S	8.69647	4.58055	-0.32132
H	10.27828	2.91122	0.47731
C	10.18514	5.26241	-1.09132
H	10.01163	6.31330	-1.33776
H	10.42388	4.72646	-2.01315
H	11.03100	5.20392	-0.40144

MDBP-derived crosslink. Distance between C8 and C17: 12.3 Å

C	0.17593	2.90383	-0.53111
C	0.60757	4.09483	0.07077
C	0.81750	2.47555	-1.70287
C	1.64862	4.84060	-0.48811
C	1.85801	3.22217	-2.26174
C	2.27617	4.41745	-1.66541
H	0.13939	4.46179	0.98267
H	0.51330	1.55484	-2.19740
H	2.33879	2.86538	-3.17070
H	1.96665	5.75948	0.00142
C	3.41540	5.20736	-2.24928
C	-0.95637	2.07423	0.05673
C	-0.58839	1.55100	1.44882
H	0.35611	0.99604	1.42200
H	-1.36276	0.87335	1.82423
H	-0.47716	2.36335	2.17523
C	-2.29422	2.81493	0.08954
F	-2.28131	3.86877	0.93523
F	-3.28313	1.98428	0.50234
F	-2.64722	3.26660	-1.13466
H	-1.10139	1.19411	-0.58396
N	4.67306	4.79497	-1.67303
H	3.27634	6.28095	-2.07464
H	3.46978	5.06736	-3.33550
C	5.84268	5.44283	-1.99393
H	4.70188	3.92752	-1.15259
O	5.88884	6.42447	-2.72637
C	7.05967	4.82517	-1.34595
C	8.33076	5.55432	-1.76555
H	7.11369	3.76943	-1.63677
H	6.93832	4.86919	-0.25728
N	9.51086	4.95202	-1.16697
H	8.28212	6.60683	-1.46239
H	8.44205	5.52314	-2.85593
C	10.15223	3.86693	-1.69555
C	10.02893	5.33041	0.03540
C	11.27840	4.54373	0.29826
C	11.23057	3.42312	-0.73051

O	9.87896	3.31656	-2.75320
O	9.59183	6.19419	0.78025
H	11.30501	4.20454	1.33735
H	12.13563	5.20081	0.11459
S	10.75283	1.81865	0.01168
H	12.17934	3.32551	-1.26715
C	12.33466	1.37194	0.76886
H	12.23691	0.39690	1.25352
H	12.62253	2.10617	1.52513
H	13.11488	1.30272	0.00649

NMD-derived crosslink. Distance between C2 and C14: 6.0 Å

C	-4.76959	-0.26244	0.41492
C	-3.88612	0.31704	-0.69253
H	-5.80680	-0.35717	0.07625
H	-4.76077	0.37571	1.30523
H	-4.42333	-1.25910	0.70828
C	-2.42538	0.38502	-0.21247
C	-1.44790	0.84812	-1.29664
H	-2.11963	-0.60974	0.13807
H	-2.34864	1.05065	0.65718
N	-0.07274	0.76210	-0.83722
H	-1.64708	1.88937	-1.57041
H	-1.56073	0.22794	-2.19278
C	0.54260	1.73868	-0.11357
C	2.04957	-0.09778	-0.34350
C	1.95947	1.33456	0.16365
H	2.62209	1.99009	-0.41155
H	2.17771	1.45217	1.22899
O	0.05671	2.80503	0.23438
O	0.43154	-1.32469	-1.68518
C	0.72654	-0.32710	-1.04134
S	2.25495	-1.30833	1.01105
H	2.85754	-0.21201	-1.07349
C	4.00637	-1.03484	1.37507
H	4.31826	-1.71775	2.16977
H	4.17501	-0.00989	1.71441
H	4.61480	-1.23432	0.48908
C	-4.42506	1.68251	-1.12122
H	-3.94116	-0.35950	-1.55481
H	-4.32862	2.41830	-0.31574
H	-5.48447	1.61430	-1.39145
H	-3.89267	2.06362	-1.99787

VTT-derived crosslink. Distance between C1 and C2: 1.5 Å

C	1.40116	0.38796	0.90559
C	2.82325	0.53934	1.43876
S	3.47802	2.18683	1.00250
H	3.48180	-0.21992	1.00326
H	2.83942	0.43952	2.52944
C	5.11431	2.03179	1.75803
H	5.67525	2.95581	1.59488
H	5.66661	1.20512	1.30374
H	5.02242	1.86649	2.83461
S	0.74640	-1.25954	1.34182
H	1.38500	0.48779	-0.18510
H	0.74260	1.14721	1.34109
C	-0.88977	-1.10462	0.58601
H	-1.45071	-2.02865	0.74915
H	-0.79770	-0.93941	-0.49057
H	-1.44218	-0.27793	1.04014

Data availability

The python scripts used for the data processing, raw data from biological assays, raw NMR data, and SDS PAGE data can be accessed via Zenodo (record #15382541, zenodo.org/records/15382541).

The mass spectrometry proteomics data have been deposited to the ProteomeXchange Consortium via the PRIDE[29] partner repository with the dataset identifier PXD063857: ebi.ac.uk/pride/archive/projects/PXD063857.

The crosslinking mass spectrometry proteomics data have been deposited to the ProteomeXchange Consortium via the PRIDE[29] partner repository with the dataset identifier PXD063839 (10.6019/PXD063839): ebi.ac.uk/pride/archive/projects/PXD063839.

REFERENCES

1. Juliá, F.; Yan, J.; Paulus, F.; Ritter, T., Vinyl Thianthrenium Tetrafluoroborate: A Practical and Versatile Vinylating Reagent Made from Ethylene. *Journal of the American Chemical Society* **2021,** *143* (33), 12992-12998.

2. Hartmann, P.; Bohdan, K.; Hommrich, M.; Juliá, F.; Vogelsang, L.; Eirich, J.; Zangl, R.; Farès, C.; Jacobs, J. B.; Mukhopadhyay, D.; Mengeler, J. M.; Vetere, A.; Sterling, M. S.; Hinrichs, H.; Becker, S.; Morgner, N.; Schrader, W.; Finkemeier, I.; Dietz, K.-J.; Griesinger, C.; Ritter, T., Chemoselective Umpolung of Thiols to Episulfoniums for Cysteine Bioconjugation. *Nature Chemistry* **2024,** *16* (3), 380-388.

3. Matzinger, M.; Vasiu, A.; Madalinski, M.; Müller, F.; Stanek, F.; Mechtler, K., Mimicked synthetic ribosomal protein complex for benchmarking crosslinking mass spectrometry workflows. *Nature Communications* **2022,** *13* (1), 3975.

4. Bio-Rad: A Guide to Polyacrylamide Gel Electrophoresis and Detection. https://www.bio-rad.com/webroot/web/pdf/lsr/literature/Bulletin_6040.pdf.

5. Yamamoto, H.; Fujiwara, T.; Funatsu, T.; Tsunoda, M., Quantification of Intracellular Thiols by HPLC-Fluorescence Detection. *Molecules* **2021,** *26* (8), 2365.

6. Hughes, C. S.; Moggridge, S.; Müller, T.; Sorensen, P. H.; Morin, G. B.; Krijgsveld, J., Single-pot, Solid-phase-enhanced Sample Preparation for Proteomics Experiments. *Nature Protocols* **2019,** *14* (1), 68-85.

7. Yan, T.; Desai, H. S.; Boatner, L. M.; Yen, S. L.; Cao, J.; Palafox, M. F.; Jami-Alahmadi, Y.; Backus, K. M., SP3-FAIMS Chemoproteomics for High-Coverage Profiling of the Human Cysteinome. *ChemBioChem* **2021,** *22* (10), 1841-1851.

8. Knieper, M.; Vogelsang, L.; Guntelmann, T.; Sproß, J.; Gröger, H.; Viehhauser, A.; Dietz, K.-J., OPDAylation of Thiols of the Redox Regulatory Network In Vitro. *Antioxidants* **2022,** *11* (5), 855.

9. Cox, J.; Mann, M., MaxQuant Enables High Peptide Identification Rates, Individualized p.p.b.-range Mass Accuracies And Proteome-Wide Protein Quantification. *Nature Biotechnology* **2008,** *26* (12), 1367-1372.

10. Binns, D.; Dimmer, E.; Huntley, R.; Barrell, D.; O'Donovan, C.; Apweiler, R., QuickGO: a web-based tool for Gene Ontology searching. *Bioinformatics* **2009,** *25* (22), 3045-3046.

11. Lex, A.; Gehlenborg, N.; Strobelt, H.; Vuillemot, R.; Pfister, H., UpSet: Visualization of Intersecting Sets. *IEEE Transactions on Visualization and Computer Graphics* **2014,** *20* (12), 1983-1992.

12. Chambers, M. C.; Maclean, B.; Burke, R.; Amodei, D.; Ruderman, D. L.; Neumann, S.; Gatto, L.; Fischer, B.; Pratt, B.; Egertson, J.; Hoff, K.; Kessner, D.; Tasman, N.; Shulman, N.; Frewen, B.; Baker, T. A.; Brusniak, M.-Y.; Paulse, C.; Creasy, D.; Flashner, L.; Kani, K.; Moulding, C.; Seymour, S. L.; Nuwaysir, L. M.; Lefebvre, B.; Kuhlmann, F.; Roark, J.; Rainer, P.; Detlev, S.; Hemenway, T.; Huhmer, A.; Langridge, J.; Connolly, B.; Chadick, T.; Holly, K.; Eckels, J.; Deutsch, E. W.; Moritz, R. L.; Katz, J. E.; Agus, D. B.; MacCoss, M.; Tabb, D. L.; Mallick, P., A Cross-platform Toolkit for Mass Spectrometry and Proteomics. *Nature Biotechnology* **2012,** *30* (10), 918-920.

13. MSFragger: Converting raw files to mzML. https://fragpipe.nesvilab.org/docs/tutorial_convert.html.

14. Lenz, S.; Sinn, L. R.; O'Reilly, F. J.; Fischer, L.; Wegner, F.; Rappsilber, J., Reliable identification of protein-protein interactions by crosslinking mass spectrometry. *Nature Communications* **2021,** *12* (1), 3564.

15. Github: XiSearch. https://github.com/Rappsilber-Laboratory/XiSearch.

16. Kong, A. T.; Leprevost, F. V.; Avtonomov, D. M.; Mellacheruvu, D.; Nesvizhskii, A. I., MSFragger: ultrafast and comprehensive peptide identification in mass spectrometry–based proteomics. *Nature Methods* **2017,** *14* (5), 513-520.

17. Mendes, M. L.; Fischer, L.; Chen, Z. A.; Barbon, M.; O'Reilly, F. J.; Giese, S. H.; Bohlke‐Schneider, M.; Belsom, A.; Dau, T.; Combe, C. W.; Graham, M.; Eisele, M. R.; Baumeister, W.; Speck, C.; Rappsilber, J., An Integrated Workflow for Crosslinking Mass Spectrometry. *Molecular Systems Biology* **2019,** *15* (9), e8994.

18. Giese, S. H.; Belsom, A.; Sinn, L.; Fischer, L.; Rappsilber, J., Noncovalently Associated Peptides Observed during Liquid Chromatography-Mass Spectrometry and Their Effect on Cross-Link Analyses. *Analytical Chemistry* **2019,** *91* (4), 2678-2685.

19. Combe, C. W.; Graham, M.; Kolbowski, L.; Fischer, L.; Rappsilber, J., xiVIEW: Visualisation of Crosslinking Mass Spectrometry Data. *Journal of Molecular Biology* **2024,** *436* (17), 168656.

20. Kozlowski, L. P., Proteome-pI: proteome isoelectric point database. *Nucleic Acids Research* **2016,** *45* (D1), D1112-D1116.

21. Jumper, J.; Evans, R.; Pritzel, A.; Green, T.; Figurnov, M.; Ronneberger, O.; Tunyasuvunakool, K.; Bates, R.; Žídek, A.; Potapenko, A.; Bridgland, A.; Meyer, C.; Kohl, S. A. A.; Ballard, A. J.; Cowie, A.; Romera-Paredes, B.; Nikolov, S.; Jain, R.; Adler, J.; Back, T.; Petersen, S.; Reiman, D.; Clancy, E.; Zielinski, M.; Steinegger, M.; Pacholska, M.; Berghammer, T.; Bodenstein, S.; Silver, D.; Vinyals, O.; Senior, A. W.; Kavukcuoglu, K.; Kohli, P.; Hassabis, D., Highly Accurate Protein Structure Prediction With AlphaFold. *Nature* **2021,** *596* (7873), 583-589.

22. Stahl, K.; Warneke, R.; Demann, L.; Bremenkamp, R.; Hormes, B.; Brock, O.; Stülke, J.; Rappsilber, J., Modelling Protein Complexes With Crosslinking Mass Spectrometry and Deep Learning. *Nature Communications* **2024,** *15* (1), 7866.

23. Pettersen, E. F.; Goddard, T. D.; Huang, C. C.; Couch, G. S.; Greenblatt, D. M.; Meng, E. C.; Ferrin, T. E., UCSF Chimera—A Visualization System for Exploratory Research and Analysis. *Journal of Computational Chemistry* **2004,** *25* (13), 1605-1612.

24. Daina, A.; Michielin, O.; Zoete, V., SwissADME: a free web tool to evaluate pharmacokinetics, drug-likeness and medicinal chemistry friendliness of small molecules. *Scientific Reports* **2017,** *7* (1), 42717.

25. Scientific, T. Crosslinking Technical Handbook. https://tools.thermofisher.com/content/sfs/brochures/1602163-Crosslinking-Reagents-Handbook.pdf.

26. Hanwell, M. D.; Curtis, D. E.; Lonie, D. C.; Vandermeersch, T.; Zurek, E.; Hutchison, G. R.,

Avogadro: an advanced semantic chemical editor, visualization, and analysis platform. *Journal of Cheminformatics* **2012,** *4* (1), 17.

27. Pajouhesh, H.; Lenz, G. R., Medicinal chemical properties of successful central nervous system drugs. *NeuroRX* **2005,** *2* (4), 541-553.

28. Benet, L. Z.; Hosey, C. M.; Ursu, O.; Oprea, T. I., BDDCS, the Rule of 5 and drugability. *Advanced Drug Delivery Reviews* **2016,** *101*, 89-98.

29. Perez-Riverol, Y.; Bandla, C.; Kundu, Deepti J.; Kamatchinathan, S.; Bai, J.; Hewapathirana, S.; John, Nithu S.; Prakash, A.; Walzer, M.; Wang, S.; Vizcaíno, Juan A., The PRIDE database at 20 years: 2025 update. *Nucleic Acids Research* **2024,** *53* (D1), D543-D553.
